# Supplementary material for: Predicting ligand-dependent tumors from multi-dimensional signaling features
Source: NPJ Syst Biol Appl. 2017 Sep 20;3:27. doi: 10.1038/s41540-017-0030-3 (PMC5607260; doi:10.1038/s41540-017-0030-3)
Supplement: Supplementary file 1 — Supplementary Figure and Text [file 41540_2017_30_MOESM1_ESM.pdf]

Predicting ligand-dependent tumors from multi-dimensional signaling features – Supplementary Materials

August 21, 2017

Contents

1 Supplementary Methods **11**
1.1 Classification via machine learning 11
1.2 RTK hetero-dimerization model 11
1.3 Experimental time-course data and chosen cell lines 12
1.4 Model description 12
1.5 Observables 32
1.6 Dynamic parameters 32
1.7 Experimental data and model fits 36
1.8 Cell line H322M 37
1.9 Cell line BxPc-3 39
1.10 Cell line A431 41
1.11 Cell line BT-20 43
1.12 Cell line IGROV-1 44
1.13 Cell line ADRr 45
1.14 Cell line ACHN 48
1.15 Cell line BT474 51
1.16 Cell line MDA-MB-231 53
1.17 Estimated parameters 54
1.17.1 Condition dependent parameter changes 70

Supplementary References **77**

Figures and Tables

Suppl. Figure1	In vitro viability screen on cell lines of five different indications.	3
Suppl. Figure2	Model predictions with random receptor surface levels.	3
Suppl. Figure3	Model comparison for basal data obatined from (1).	4
Suppl. Figure4	Model response to BTC stimulation.	4
Suppl. Figure5	Event classification by bagged decision tree algorithm.	5
Suppl. Figure6	BDT prediction efficiency for various inputs.	6
Suppl. Figure7	Difference of machine learning guided proliferation prediction to random assessment.	6
Suppl. Figure8	Percentage of true positive predictions for different BDT setups.	7
Suppl. Figure9	Correlation of ligand expression and growth prediction for breast, colorectal, lung and ovarian cancer in TCGA data set.	8
Suppl. Figure10	Correlation between mRNA measurements from the CCLE database and receptor/surface measurements acquired via qFACS.	9
Suppl. Table S1	Listing of capture and detection antibodies used in the ELISA assays	10
Suppl. Table S2	Model observables and error models	32
Suppl. Table S3	Estimated dynamic parameter values	32
Suppl. Figure11	Model trajectories for cell line H322M after EGF stimulation.	37
Suppl. Figure12	Model trajectories for cell line H322M after HRG stimulation.	37
Suppl. Figure13	Model trajectories for cell line H322M after IGF-1 stimulation.	38
Suppl. Figure14	Model trajectories for cell line H322M after ligand co-stimulations.	38
Suppl. Figure15	Model trajectories for cell line BxPc-3 after EGF stimulation.	39
Suppl. Figure16	Model trajectories for cell line BxPc-3 after HRG stimulation.	39
Suppl. Figure17	Model trajectories for cell line BxPc-3 after IGF-1 stimulation.	40
Suppl. Figure18	Model trajectories for cell line BxPc-3 after ligand co-stimulations.	40
Suppl. Figure19	Model trajectories for cell line A4431 after EGF stimulation.	41
Suppl. Figure20	Model trajectories for cell line A4431 after HRG stimulation.	41
Suppl. Figure21	Model trajectories for cell line A4431 after IGF-1 stimulation.	42
Suppl. Figure22	Model trajectories for cell line BT-20 after EGF stimulation.	43
Suppl. Figure23	Model trajectories for cell line BT-20 after IGF-1 stimulation.	43
Suppl. Figure24	Model trajectories for cell line IGROV-1 after EGF stimulation.	44
Suppl. Figure25	Model trajectories for cell line IGROV-1 after HRG stimulation.	44
Suppl. Figure26	Model trajectories for cell line ADRr after EGF stimulation.	45
Suppl. Figure27	Model trajectories for cell line ADRr after HRG stimulation.	45
Suppl. Figure28	Model trajectories for cell line ADRr after HRG stimulation.	46
Suppl. Figure29	Model trajectories for cell line ADRr after IGF-1 stimulation.	46
Suppl. Figure30	Model trajectories for cell line ADRr after BTC stimulation.	47
Suppl. Figure31	Model trajectories for cell line ACHN after HGF stimulation.	48
Suppl. Figure32	Model trajectories for cell line ACHN after HGF stimulation.	48
Suppl. Figure33	Model trajectories for cell line ACHN after HGF stimulation.	49
Suppl. Figure34	Model trajectories for cell line ACHN after HGF+EGF co-stimulation.	49
Suppl. Figure35	Model trajectories for cell line ACHN after EGF stimulation.	50
Suppl. Figure36	Model trajectories for cell line ACHN after IGF-1 stimulation.	50
Suppl. Figure37	Model trajectories for cell line BT474 after EGF stimulation.	51
Suppl. Figure38	Model trajectories for cell line BT474 after HRG stimulation.	51
Suppl. Figure39	Model trajectories for cell line BT474 after IGF-1 stimulation.	52
Suppl. Figure40	Model trajectories for cell line MDA-MB-231 after EGF stimulation.	53
Suppl. Table S4	Estimated parameter values	54
Suppl. Figure41	Receptor surface levels of the various cell lines used for the mechanistic model.	70

Supplemental Figures and Tables

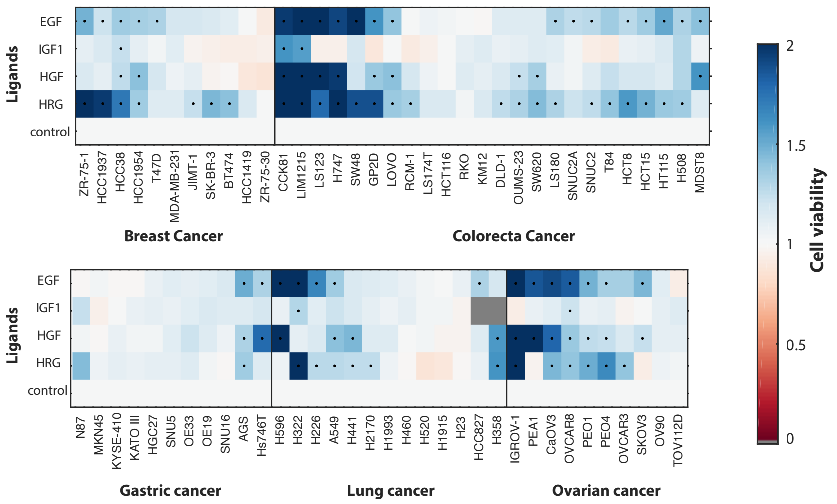

Suppl. Figure 1: In vitro viability screen on cell lines of five different indications. Growth upon ligand stimulation with HRG, HGF, IGF-1 or EGF, respectively, is shown in blue shades with a dot if the growth exceeds 20 % with respect to the control and is significant with confidence level =0.05 in quadruplicate measurements. This is a subset of the data shown in Fig. 1.

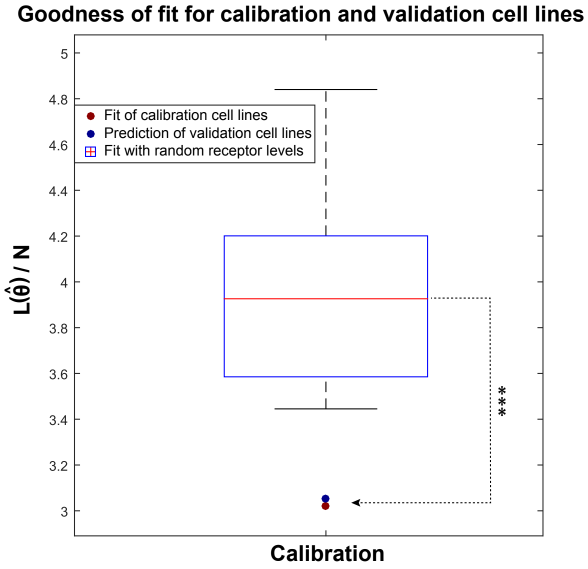

Suppl. Figure 2: Model predictions with random receptor surface levels. Model fits for calibration and validation cell lines are shown as red and blue dots, respectively, with a boxplot indicating the change in goodness of fit after assignment of random receptor surface levels taken from a uniform distribution of the receptor levels of all available cell lines.

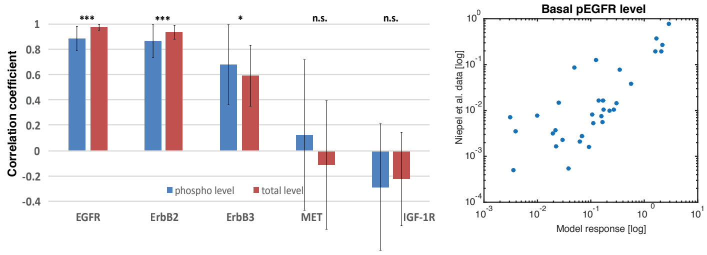

Suppl. Figure 3: Model comparison for basal data obatined from (1). Basal phospho- and total receptor levels were calculated from the model's analytic steady state equations for cell lines with available RNAseq values from the CCLE database. Spearman correlations were computed between model response and ELISA measurements, with uncertainties as bars. The ErbB receptor family shows good correlation for both measures, emphasizing the ability of the model to transform single receptor levels into cell response. Yet, c-Met and IGF-1R do not show significant correlation, which on the one hand might be caused by problems in the calculation of receptor surface levels. On the other hand, an imprecise receptor signaling network for c-Met and IGF-1R due to insufficient data and experimental conditions can reduce the correlation. Exemplary correlation are shown for basal levels of phosphorylated EGFR on the right.

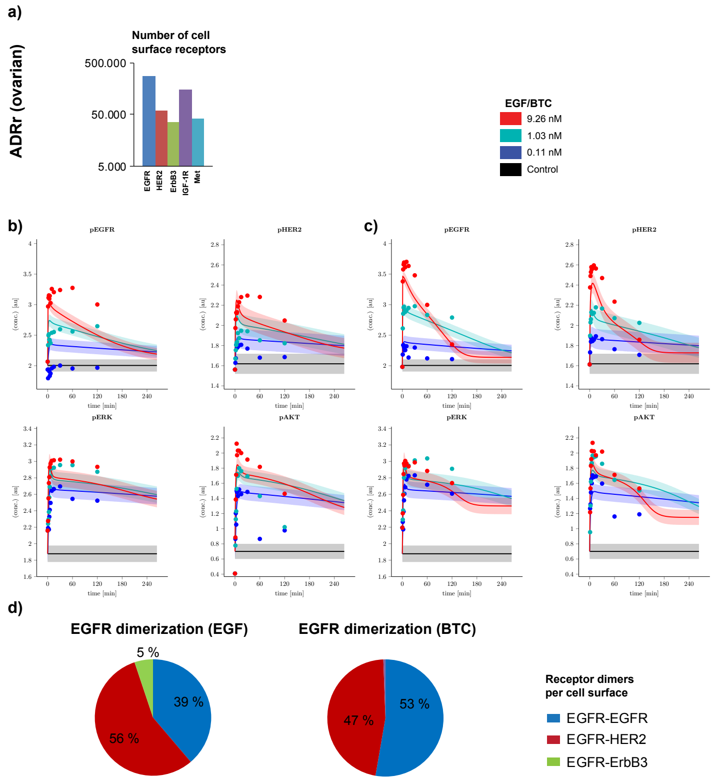

Suppl. Figure 4: Model response to BTC stimulation. a: Receptor surface levels of ADRr cell line and ligand concentrations of EGF and BTC. **b:** Model response and experimental data after EGF stimulation. Data is shown as dots, model time-courses in colors with respective uncertainties as shaded areas. **c:** Model response and data after BTC stimulation. **d:** Differences of EGFR activation pattern for EGF and BTC stimulation, with BTC showing less hetero-dimerization.

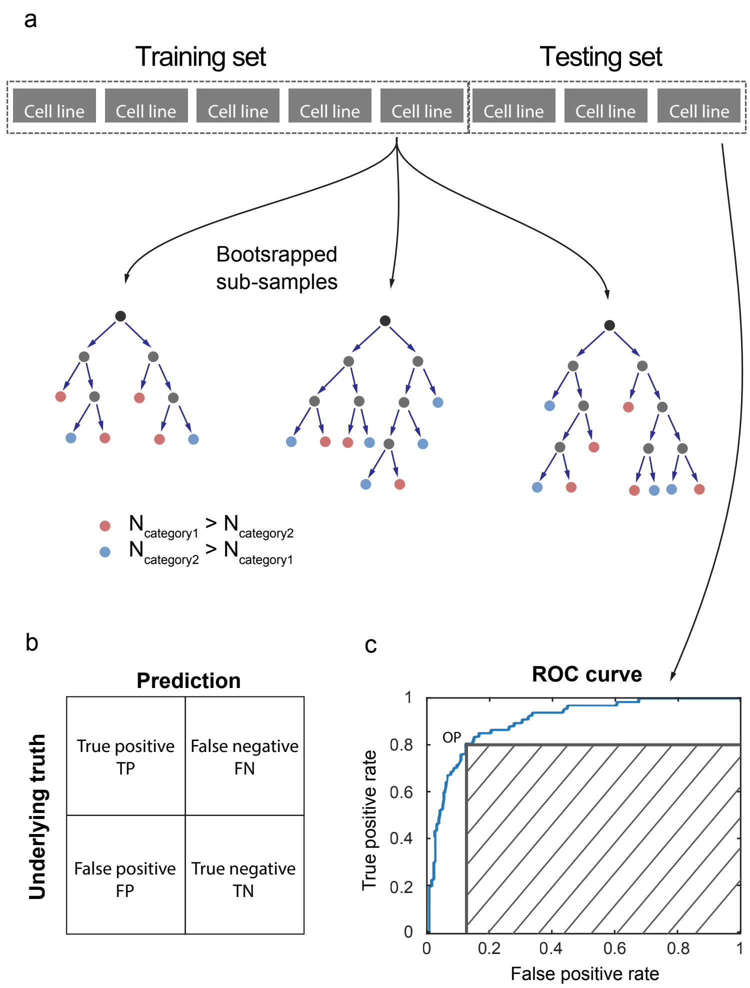

Suppl. Figure 5: Event classification by bagged decision tree algorithm. a: Scheme of cell lines divided into training and testing set, with bootstrapped sub-samples of the former being fed into decision trees that split the data according to specified features. **b:** Allocation of BDT predictions to true/false positive/negative events, depending on the prediction and underlying truth. **c:** Receiver Operator Characteristics (ROC) curve showing the amount of events ending up as true and false positive depending on the threshold of the BDT output between classifications with set working point maximizing the squared area (WP).

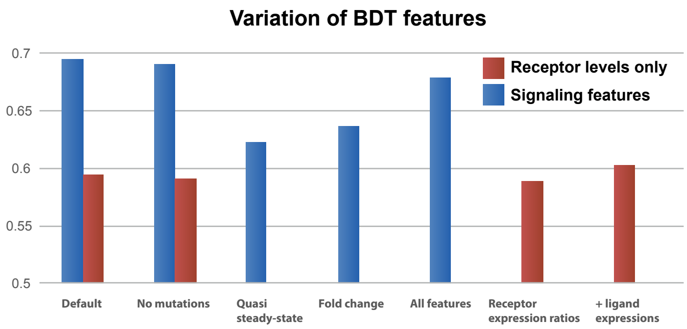

Suppl. Figure 6: BDT prediction efficiency for various inputs. The percentage of true predictions of the trained BDT (random prediction equals 0.5) is shown for several model-derived and RNA based features. Quasi steady-state refers to the final concentrations of model components after ligand stimulation, fold-change to the maximal induced relative change in model components. Regarding receptor levels, the ratios of the distinct receptor expressions were taken into account, and the RNAseq measurements of ligand expression within the samples.

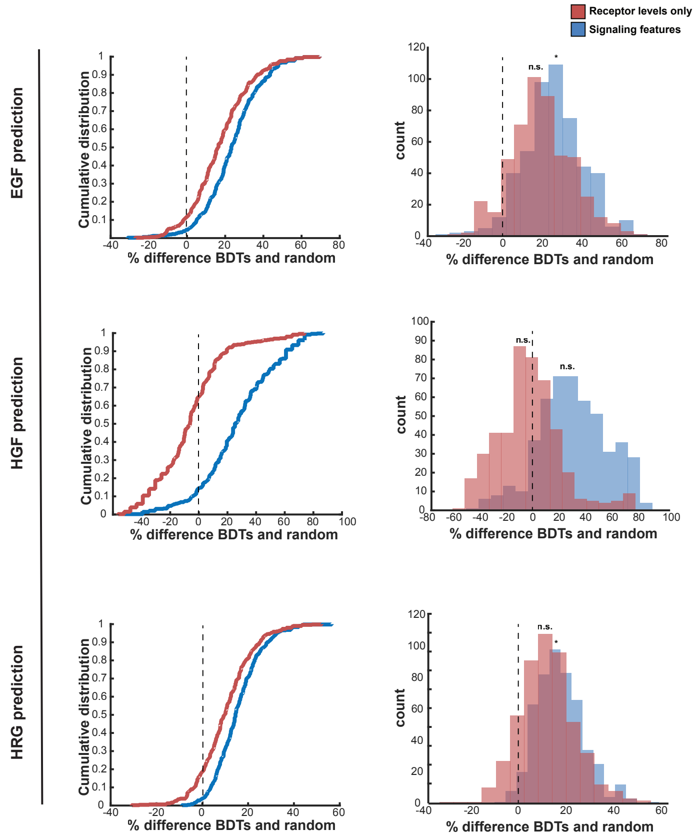

Suppl. Figure 7: Difference of machine learning guided proliferation prediction to random assessment. For EGF, HGF and HRG, difference of the mean of true and false predictive values for cellular growth are shown for both model-derived features and receptor expression levels. These are compared to random prediction of cell proliferation (50 % correct), for n=500 random splits between training and testing cell lines and as histogram (left panels) with statistical significance or as cumulative distributions (right panels).

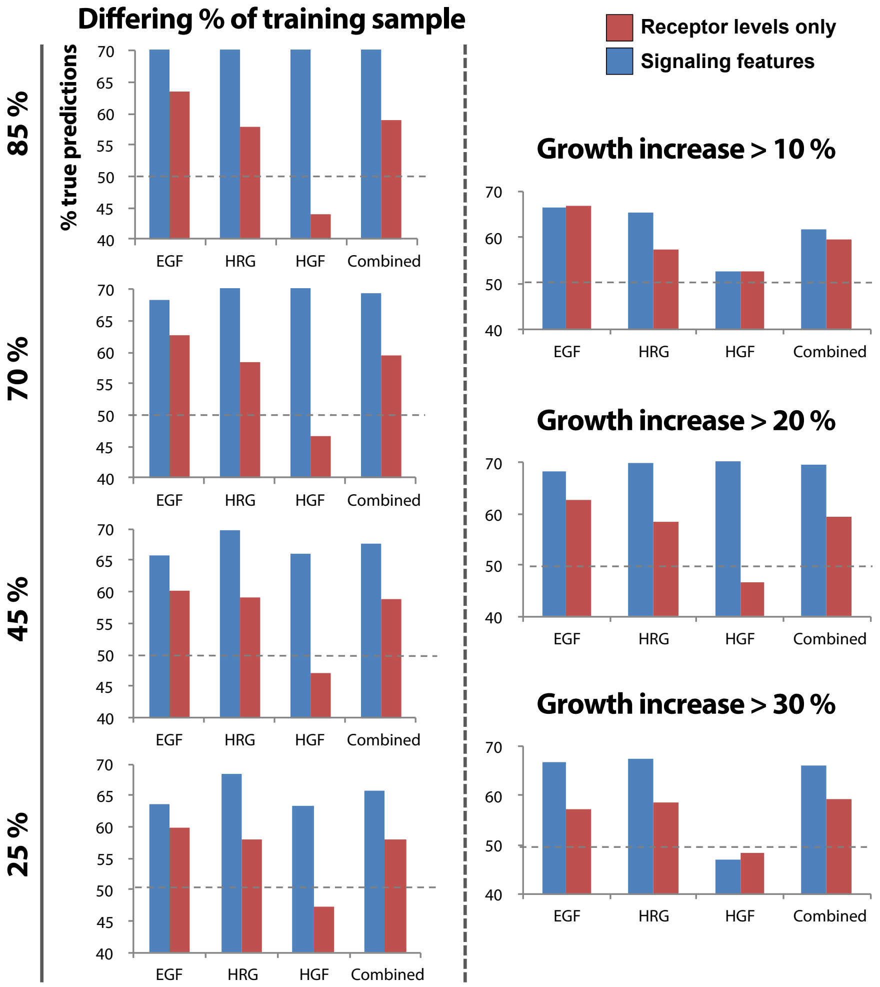

Suppl. Figure 8: Percentage of true positive predictions for different BDT setups. Left: Fraction of training cell lines ranging from 85 % down to 25 % with respective true growth predictions for both model response and receptor surface levels as training features, shown for each ligand separately and combined. **Right:** BDT training with growth specification for either > 10 %, > 20 % or > 30 % cell growth increase compared to control growth.

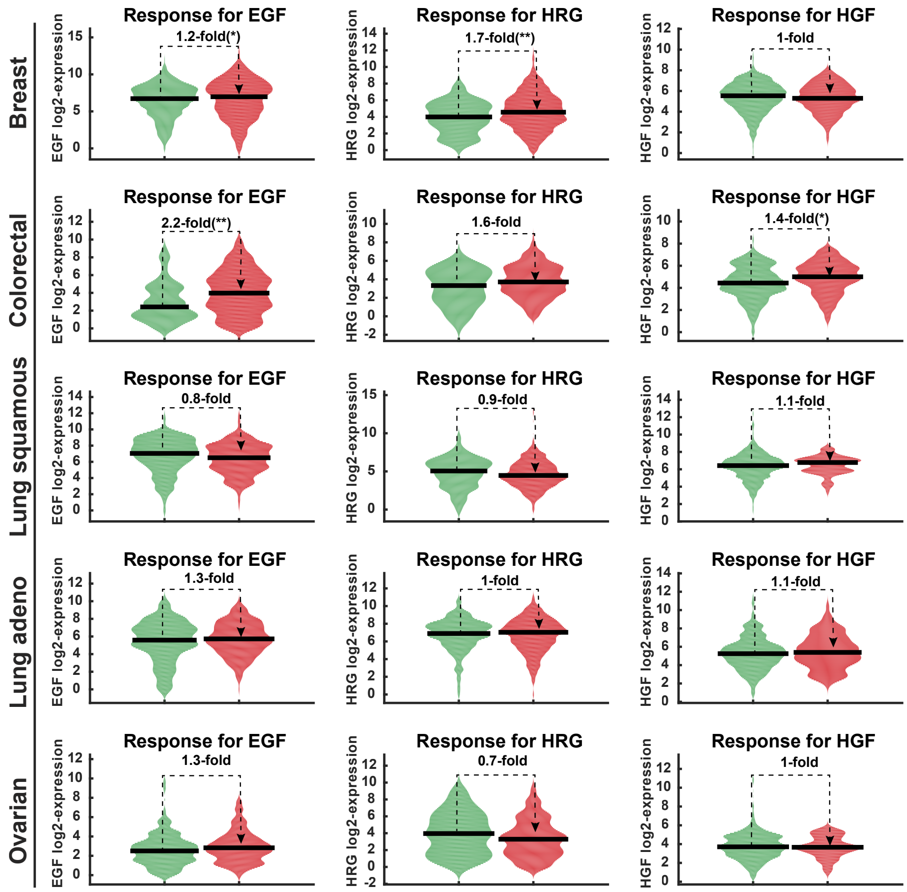

Suppl. Figure 9: Correlation of ligand expression and growth prediction for breast, colorectal, lung and ovarian cancer in TCGA data set. The measured RNA expression of the respective ligands is plotted for the responders and non-responders with stars indicating significance of difference.

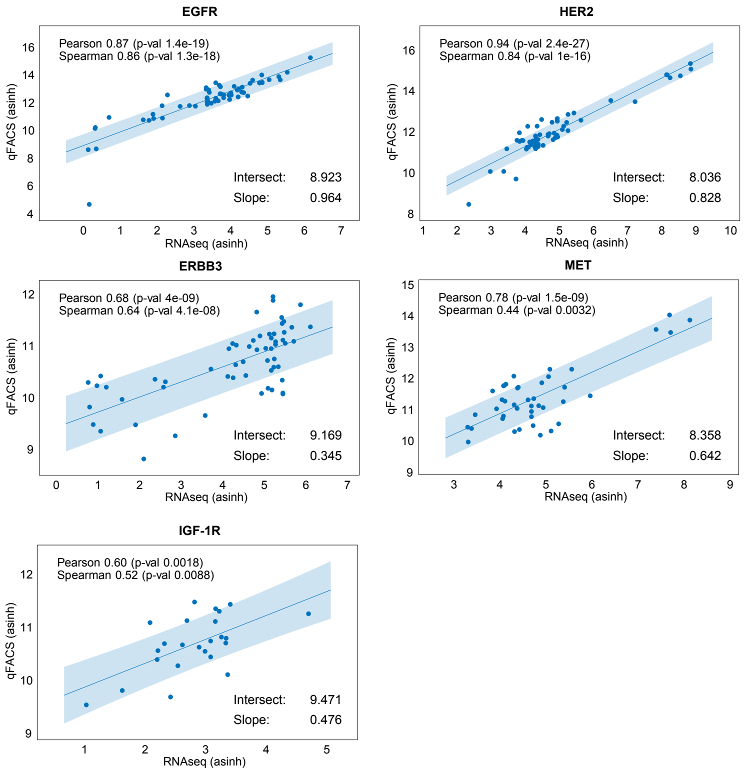

Suppl. Figure 10: Correlation between mRNA measurements from the CCLE database and receptor/surface measurements acquired via qFACS.

Suppl. Table 1: Listing of capture and detection antibodies used in the ELISA assays.

	Capture Ab		Detection Ab		Secondary detection	
Assay	Supplier	Catalog #	Supplier	Catalog #	Supplier	Catalog #
Total EGFR	R&D Systems	AF231	R&D Systems	BAF231	R&D Systems	DY998
Total ErbB2	R&D Systems	MAB1129	R&D Systems	BAF1129	R&D Systems	DY998
Total ErbB3	R&D Systems	MAB3481	R&D Systems	BAM348	R&D Systems	DY998
Total IGF-1R	R&D Systems	DYC305	R&D Systems	DYC305	R&D Systems	DY998
phospho EGFR	R&D Systems	AF231	Millipore	16-452	R&D Systems	DY998
phospho ErbB2	R&D Systems	MAB1129	Millipore	16-452	R&D Systems	DY998
phospho ErbB3	R&D Systems	MAB3481	Millipore	16-452	R&D Systems	DY998
phospho IGF-1R	R&D Systems	DYC305	Millipore	16-452	R&D Systems	DY998

1 Supplementary Methods

1.1 Classification via machine learning

Supervised learning was conducted by bootstrap aggregating (bagged) decision trees (BDTs), a widely-used machine learning algorithm based on random forests used for data classification (2; 3; 4). Therein, all available data is divided into training sets for parameter estimation and testing sets to assess the efficiency of the classification. Concerning the training set, multiple features can be utilized by the algorithm to classify the events in distinct categories that are in this work specified as 0 (no growth) and 1 (growth) and known by the algorithm. Parameter estimation is performed through a large sample N_{trees} of single trees, each of them focusing on a bootstrapped subset of all training events. On each layer of a single tree, the algorithm iterates through all given features and chooses the one with highest capability of separating the events by their respective classification. In addition, a cost function is calculated to prevent the tree from overfitting, in which single events would be categorized in a detail that is not reproducible in independent events. Every branch of a tree ends in a leaf that gets categorized depending on most events being assorted to it (see Suppl. Fig. S5, A). Succeeding the training, all testing events pass through the multitude of trees and get a final score $S(x)$ by weighted addition of all classifications of the leaves they fall into ($h_i(x)$) in each single tree. The weights depend on the achieved signal-to-noise ratio during training of the respective leaves:

$$S(x) = \frac{1}{N_{\text{trees}}} \sum_{i=1}^{N_{\text{trees}}} \ln(\alpha_i) \times h_i(x), \quad h_i(x) \in [0,1], \quad \alpha_i = \frac{1 - \text{err}_i}{\text{err}_i}, \quad \text{err}_i = \frac{N_{\text{mis-class}}}{N_{\text{all-events}}} .$$

From the receiver-operating-curve of the testing set, an optimal working point is defined categorizing every event with a score above a given threshold as 1, all others as 0 (see Suppl. Fig. S5, B). In conclusion, the BDT carves out regions in feature-space in the given data that are prone for growth, with dimensions given by the number of features. In this work, all available cell lines were split randomly and used for either training or testing. Thus, bias due to a cell line appearing with different stimuli in both sets was averted. In addition, uncertainty analysis was conducted by random sampling of training and testing cell lines 500 times with subsequent execution of the described algorithm. The training was conducted within the machine learning toolbox of MATLAB (5). A collection of 300 trees was trained in each run with a minimum of 5 events in each terminal leaf and a bootstrap ratio of 0.8. The BDT training was robust with respect to the relative amount of training and testing data and to the significance threshold upon which an event is labeled as growing (see Suppl. Fig. S8). For the latter, marking a cell line as growing if its growth increase exceeded 10 % with respect to the control leads to an introduction of substantial noise in the data set, rendering both model and receptor level based training as roughly equivalent with naïve growth prediction. Growth-increase larger than 30 % on the other hand increased the benefits of machine learning as well as the advantage of mechanistic input compared to receptor levels for all ligands but HGF. For HGF, the reduction of events leads to low numbers of true predictions with an average of one event, thus introducing high statistical uncertainty.

1.2 RTK hetero-dimerization model

In this Supplement, further details about the mechanistic signaling model are given. In the model, ligands bind to their respective receptors with given binding affinities (kD), namely 0.3 nM for IGF-1 binding to IGF-1R (6), 0.3 nM for HGF binding to c-Met (7), 0.05 nM for HRG binding to ErbB3 (8) and 1nM for binding of EGF as well as BTC to EGFR (9). Receptors with bound ligand can form several homo- and heterodimers for which biological evidence can be found in the literature (see Fig. 2 in the main text). The only exception is the HER2 receptor, which has no known ligand and is constantly able to bind to other receptors and a HGF-independent hetero-dimer of ErbB3 and Met (10; 11; 12; 13). All activated dimers are prone to undergo endocytosis. After internalization, the receptor dimers can get either dephosphorylated with subsequent recycling to the cell surface or are degraded (14; 15). Downstream of the receptor, all homo- and heterodimers except of the ErbB3-homodimer, which cannot

trans-phosphorylate due to its lack of intrinsic kinase activity, can activate the mitogen-activated protein kinase pathway (MAPK) via MEK and ERK as well as the phosphoinositide 3-kinase pathway (PI3K) via Akt. This leads to phosphorylation of S6K1 and S6 in turn. Several regulating feedbacks between the pathways are implemented (16).

In the following, detailed description of the ordinary differential equations, kinetic parameters, steady state transformations and receptor/surface level constraints will be given.

1.3 Experimental time-course data and chosen cell lines

The available data consists of three different data sets, which include time-resolved concentration measurements of activated and total receptors as well as of various phosphorylated downstream targets. The largest data set comprises nine cell lines with measurements of the EGFR, HER2 and ErbB3 receptors of the ErbB family and the IGF1-receptor, together with the downstream targets ERK, AKT, S6K1 and S6. Four different ligand concentrations of Epithelial growth factor (EGF), Heregulin (HRG), and Insulin-like growth factor (IGF-1) ranging from 0.156 to 10 nM are used and 12 measurement time points up to 240 minutes are taken. In addition, co-stimulations of the respective ligands are available for two of the nine cell lines. Out of the nine cell lines, six cell lines are used for model calibration, the remaining three to validate the model. Cell lines used for calibration include H322M (non-small cell lung cancer), BxPc-3 (pancreatic cancer), A431 (epidermoid cancer), BT-20 (breast cancer), ADRr (ovarian cancer) and IGROV-1 (ovarian cancer). BT474 (breast cancer), MDA-MB-231 (breast cancer) and ACHN (renal cancer) are utilized to validate the model.

Measurements after either EGF or Betacellulin (BTC) stimulation are available for one of the calibration cell lines, ADRr, with ligand concentrations between 0.11 and 9.26 nM, and 12 measurement time points up to 240 minutes. These include phosphorylation of EGFR, HER2, ErbB3, ERK and AKT. Apart from that, measurements with HGF and EGF as well as their co-stimulation is available for ACHN, which is used for validation with respect to HRG and IGF-1, spanning the same concentrations and measurements up to 120 minutes. Therein, phosphorylated EGFR and Met phosphorylation as well as phospho-ERK and phospho-AKT are measured. The receptor concentrations in all experiments are measured by ELISA whereas the downstream components are measured by lysate microarray

1.4 Model description

The model used in this study is based on a system of Ordinary Differential Equations (ODE). These ordinary differential equations are derived by means of the law of mass-action. The time evolution of the biochemical compounds is computed by numerically integrating these differential equations. The model contains parameters which are estimated by calibrating the model to data using a Maximum-Likelihood estimation approach. All analyses were performed using the *Data 2 Dynamics* software package (17), which is available from https://bitbucket.org/d2d-development/d2d-software.
The model consists of 61 differential equations, which are given by the following equations:

$$\mathrm{d[dose\_EGF]/dt} = -v_6 + v_7 \tag{1}$$

$$\mathrm{d[dose\_HGF]/dt} = -v_{14} + v_{15} \tag{2}$$

$$\mathrm{d[RTKph]/dt} = -v_{43} + v_{44} - v_{47} + v_{48} - v_{53} + v_{54} - v_{58} + v_{59} - v_{63} \tag{3}$$

$$+ v_{64} - v_{67} + v_{68} - v_{71} + v_{72} - v_{75} + v_{76} - v_{80} + v_{81} - v_{84} + v_{85} \tag{4}$$

$$\mathrm{d[dose\_IGF1]/dt} = -v_{12} + v_{13} \tag{5}$$

$$\mathrm{d[dose\_HRG]/dt} = -v_{10} + v_{11} \tag{6}$$

$$\mathrm{d[dose\_BTC]/dt} = -v_8 + v_9 \tag{7}$$

$$\mathrm{d[EGFR]/dt} = v_1 - v_6 + v_7 - v_8 + v_9 - 2 \cdot v_{32} - v_{35} - v_{36} - v_{40} + v_{45} \tag{8}$$

$$\mathrm{d[EGFR\_EGF]/dt} = v_6 - v_7 - 2 \cdot v_{16} - v_{21} - v_{23} - v_{30} \tag{9}$$

$$\mathrm{d[EGFR\_BTC]/dt} = v_8 - v_9 - 2 \cdot v_{17} - v_{22} - v_{24} - v_{25} - v_{31} \tag{10}$$

$$\mathrm{d[pEGFRd]/dt} = v_{16} + v_{17} + v_{32} - v_{41} \tag{11}$$

$$\mathrm{d[pEGFRi]/dt} = v_{41} - v_{42} - v_{43} \tag{12}$$

$$d[pEGFRi\_ph]/dt = v_{43} - v_{44} \quad (13)$$
$$d[EGFRi]/dt = +2 \cdot v_{44} - v_{45} + v_{64} + v_{72} + v_{85} \quad (14)$$
$$d[ErbB2]/dt = v_2 - 2 \cdot v_{18} - v_{21} - v_{22} - v_{26} - v_{35} - v_{37} + v_{50} \quad (15)$$
$$d[pErbB2]/dt = v_{18} - v_{46} \quad (16)$$
$$d[pErbB2i]/dt = v_{46} - v_{47} - v_{49} \quad (17)$$
$$d[ErbB2i]/dt = +2 \cdot v_{48} - v_{50} + v_{64} + v_{68} \quad (18)$$
$$d[pErbB2i\_ph]/dt = v_{47} - v_{48} \quad (19)$$
$$d[pErbB12]/dt = v_{21} + v_{22} + v_{35} - v_{61} \quad (20)$$
$$d[pErbB12i]/dt = v_{61} - v_{62} - v_{63} \quad (21)$$

$$d[pErbB12i\_ph]/dt = v_{63} - v_{64} \quad (22)$$
$$d[ErbB3]/dt = v_3 - v_{10} + v_{11} - v_{25} - 2 \cdot v_{33} - v_{36} - v_{37} - v_{38} + v_{55} \quad (23)$$
$$d[ErbB3\_HRG]/dt = v_{10} - v_{11} - 2 \cdot v_{19} - v_{23} - v_{24} - v_{26} - v_{28} - v_{29} \quad (24)$$
$$d[pErbB3d]/dt = v_{19} + v_{33} - v_{51} \quad (25)$$
$$d[pErbB3i]/dt = v_{51} - v_{52} - v_{53} \quad (26)$$
$$d[pErbB3i\_ph]/dt = v_{53} - v_{54} \quad (27)$$
$$d[ErbB3i]/dt = +2 \cdot v_{54} - v_{55} + v_{68} + v_{72} + v_{81} \quad (28)$$
$$d[pErbB13]/dt = v_{23} + v_{24} + v_{25} + v_{36} - v_{69} \quad (29)$$
$$d[pErbB13i]/dt = v_{69} - v_{70} - v_{71} \quad (30)$$
$$d[pErbB13i\_ph]/dt = v_{71} - v_{72} \quad (31)$$

$$d[pErbB32]/dt = v_{26} + v_{37} - v_{65} \quad (32)$$
$$d[pErbB32i]/dt = v_{65} - v_{66} - v_{67} \quad (33)$$
$$d[pErbB32i\_ph]/dt = v_{67} - v_{68} \quad (34)$$
$$d[IGF1R]/dt = v_4 - v_{12} + v_{13} - 2 \cdot v_{34} + v_{60} \quad (35)$$
$$d[IGF1R\_IGF1]/dt = v_{12} - v_{13} - 2 \cdot v_{20} \quad (36)$$
$$d[pIGF1Rd]/dt = v_{20} + v_{34} - v_{56} \quad (37)$$
$$d[pIGF1Ri]/dt = v_{56} - v_{57} - v_{58} \quad (38)$$
$$d[pIGF1Ri\_ph]/dt = v_{58} - v_{59} \quad (39)$$
$$d[IGF1Ri]/dt = +2 \cdot v_{59} - v_{60} \quad (40)$$
$$d[Met]/dt = v_5 - v_{14} + v_{15} - v_{28} - v_{38} - 2 \cdot v_{39} - v_{40} + v_{77} \quad (41)$$

$$d[Met\_HGF]/dt = v_{14} - v_{15} - 2 \cdot v_{27} - v_{29} - v_{30} - v_{31} \quad (42)$$
$$d[pMetd]/dt = v_{27} + v_{39} - v_{73} \quad (43)$$
$$d[pMeti]/dt = v_{73} - v_{74} - v_{75} \quad (44)$$
$$d[pMeti\_ph]/dt = v_{75} - v_{76} \quad (45)$$
$$d[Meti]/dt = +2 \cdot v_{76} - v_{77} + v_{81} + v_{85} \quad (46)$$
$$d[pMetErbB3]/dt = v_{28} + v_{29} + v_{38} - v_{78} \quad (47)$$
$$d[pMetErbB3i]/dt = v_{78} - v_{79} - v_{80} \quad (48)$$
$$d[pMetErbB3i\_ph]/dt = v_{80} - v_{81} \quad (49)$$
$$d[pMetEGFR]/dt = v_{30} + v_{31} + v_{40} - v_{82} \quad (50)$$
$$d[pMetEGFRi]/dt = v_{82} - v_{83} - v_{84} \quad (51)$$

$$d[pMetEGFRi\_ph]/dt = v_{84} - v_{85} \quad (52)$$
$$d[MEK]/dt = -v_{86} - v_{87} - v_{88} - v_{89} - v_{90} - v_{91} - v_{92} - v_{93} - v_{94} + v_{95} \quad (53)$$
$$d[pMEK]/dt = v_{86} + v_{87} + v_{88} + v_{89} + v_{90} + v_{91} + v_{92} + v_{93} + v_{94} - v_{95} \quad (54)$$
$$d[ERK]/dt = -v_{96} + v_{97} \quad (55)$$
$$d[pERK]/dt = v_{96} - v_{97} \quad (56)$$

$$\mathrm{d[AKT]/dt} = -v_{98} - v_{99} - v_{100} - v_{101} - v_{102} - v_{103} - v_{104} - v_{105} - v_{106} + v_{107} \tag{57}$$
$$\mathrm{d[pAKT]/dt} = v_{98} + v_{99} + v_{100} + v_{101} + v_{102} + v_{103} + v_{104} + v_{105} + v_{106} - v_{107} \tag{58}$$
$$\mathrm{d[S6K1]/dt} = -v_{108} - v_{109} + v_{110} \tag{59}$$
$$\mathrm{d[pS6K1]/dt} = v_{108} + v_{109} - v_{110} \tag{60}$$
$$\mathrm{d[S6]/dt} = -v_{111} - v_{112} + v_{113} \tag{61}$$
$$\mathrm{d[pS6]/dt} = v_{111} + v_{112} - v_{113} \tag{62}$$

Based on numerical integration of these equations, 5 derived quantities are computed and used to constrain the receptor surface levels of each cell line:

$$[\text{facs\_der\_egfr}](t) = [\text{EGFR}] + [\text{EGFR\_BTC}] + [\text{EGFR\_EGF}] + 2 \cdot [\text{pEGFRd}] \tag{63}$$

$$+[\text{pErbB12}] + [\text{pErbB13}] + [\text{pMetEGFR}] \tag{64}$$

$$[\text{facs\_der\_erbb2}](t) = [\text{ErbB2}] + [\text{pErbB12}] + 2 \cdot [\text{pErbB2}] + [\text{pErbB32}] \tag{65}$$

$$[\text{facs\_der\_erbb3}](t) = [\text{ErbB3}] + [\text{ErbB3\_HRG}] + [\text{pErbB13}] + [\text{pErbB32}] + 2 \cdot [\text{pErbB3d}] + [\text{pMetErbB3}] \tag{66}$$

$$[\text{facs\_der\_met}](t) = [\text{Met}] + [\text{pMetEGFR}] + [\text{pMetErbB3}] + 2 \cdot [\text{pMetd}] \tag{67}$$

$$[\text{facs\_der\_igf1r}](t) = [\text{IGF1R}] + [\text{IGF1R\_IGF1}] + 2 \cdot [\text{pIGF1Rd}] \tag{68}$$

$$\tag{69}$$

The model contains of 113 flux expressions corresponding to these equations. Reactions describe interactions between dynamics variables and build up the ODE systems. The following list indicates the reaction laws and their corresponding reaction rate equations. Promoting rate modifiers are indicated in black above the rate law arrow. Inhibitory rate modifiers are indicated in red below the rate law arrow. In the reaction rate equations dynamic and input variables are indicated by square brackets. The remaining variables are model parameters that remain constant over time. The system of ODEs was integrated using the CVODES algorithm from the SUNDIALS suite of solvers (18). First order derivatives were computed using the sensitivity equations and used for numerical optimization. Relative and absolute tolerances were set to 1e-06 and 1e-06 respectively.

- **Reaction 1:**

  $$\varnothing \longrightarrow \text{EGFR}$$

  $$v_1 = \text{EGFR\_prod}$$

- **Reaction 2:**

  $$\varnothing \longrightarrow \text{ErbB2}$$

  $$v_2 = \text{ErbB2\_prod}$$

- **Reaction 3:**

  $$\varnothing \longrightarrow \text{ErbB3}$$

  $$v_3 = \text{ErbB3\_prod}$$

- **Reaction 4:**

  $$\varnothing \longrightarrow \text{IGF1R}$$

  $$v_4 = \text{IGF1R\_prod}$$

- **Reaction 5:**

$$\varnothing \longrightarrow \text{Met}$$

$v_5 = \text{Met\_prod}$

- **Reaction 6:**

$$\text{dose\_EGF} + \text{EGFR} \longrightarrow \text{EGFR\_EGF}$$

$v_6 = [\text{EGFR}] \cdot \text{EGFR\_lig\_binding} \cdot [\text{dose\_EGF}]$

- **Reaction 7:**

$$\text{EGFR\_EGF} \longrightarrow \text{dose\_EGF} + \text{EGFR}$$

$v_7 = [\text{EGFR\_EGF}] \cdot \text{EGFR\_lig\_binding} \cdot \text{EGF\_kD}$

- **Reaction 8:**

$$\text{dose\_BTC} + \text{EGFR} \longrightarrow \text{EGFR\_BTC}$$

$v_8 = [\text{EGFR}] \cdot \text{EGFR\_BTC\_binding} \cdot [\text{dose\_BTC}]$

- **Reaction 9:**

$$\text{EGFR\_BTC} \longrightarrow \text{dose\_BTC} + \text{EGFR}$$

$v_9 = [\text{EGFR\_BTC}] \cdot \text{EGFR\_BTC\_binding} \cdot \text{EGF\_kD}$

- **Reaction 10:**

$$\text{dose\_HRG} + \text{ErbB3} \longrightarrow \text{ErbB3\_HRG}$$

$v_{10} = [\text{ErbB3}] \cdot \text{ErbB3\_lig\_binding} \cdot [\text{dose\_HRG}]$

- **Reaction 11:**

$$\text{ErbB3\_HRG} \longrightarrow \text{dose\_HRG} + \text{ErbB3}$$

$v_{11} = [\text{ErbB3\_HRG}] \cdot \text{ErbB3\_lig\_binding} \cdot \text{HRG\_kD}$

- **Reaction 12:**

$$\text{dose\_IGF1} + \text{IGF1R} \longrightarrow \text{IGF1R\_IGF1}$$

$$v_{12} = [\text{IGF1R}] \cdot \text{IGF1R\_lig\_binding} \cdot [\text{dose\_IGF1}]$$

- **Reaction 13:**

$$\text{IGF1R\_IGF1} \longrightarrow \text{dose\_IGF1} + \text{IGF1R}$$

$$v_{13} = [\text{IGF1R\_IGF1}] \cdot \text{IGF1R\_lig\_binding} \cdot \text{IGF1\_kD}$$

- **Reaction 14:**

$$\text{dose\_HGF} + \text{Met} \longrightarrow \text{Met\_HGF}$$

$$v_{14} = [\text{Met}] \cdot \text{Met\_lig\_binding} \cdot [\text{dose\_HGF}]$$

- **Reaction 15:**

$$\text{Met\_HGF} \longrightarrow \text{dose\_HGF} + \text{Met}$$

$$v_{15} = \text{HGF\_kD} \cdot [\text{Met\_HGF}] \cdot \text{Met\_lig\_binding}$$

- **Reaction 16:**

$$2 \cdot \text{EGFR\_EGF} \longrightarrow \text{pEGFRd}$$

$$v_{16} = [\text{EGFR\_EGF}]^2 \cdot \text{EGFR\_dimerize}$$

- **Reaction 17:**

$$2 \cdot \text{EGFR\_BTC} \longrightarrow \text{pEGFRd}$$

$$v_{17} = [\text{EGFR\_BTC}]^2 \cdot \text{EGFR\_BTC\_dimerize}$$

- **Reaction 18:**

$$2 \cdot \text{ErbB2} \longrightarrow \text{pErbB2}$$

$$v_{18} = [\text{ErbB2}]^2 \cdot \text{ErbB2\_dimerize}$$

- **Reaction 19:**

$$2 \cdot \text{ErbB3\_HRG} \longrightarrow \text{pErbB3d}$$

$$v_{19} = [\text{ErbB3\_HRG}]^2 \cdot \text{ErbB3\_dimerize}$$

- **Reaction 20:**

$$2 \cdot \text{IGF1R\_IGF1} \longrightarrow \text{pIGF1Rd}$$

$$v_{20} = [\text{IGF1R\_IGF1}]^2 \cdot \text{IGF1R\_dimerize}$$

- **Reaction 21:**

$$\text{EGFR\_EGF} + \text{ErbB2} \longrightarrow \text{pErbB12}$$

$$v_{21} = [\text{EGFR\_EGF}] \cdot \text{EGFR\_ErbB2\_dimerize} \cdot [\text{ErbB2}]$$

- **Reaction 22:**

$$\text{EGFR\_BTC} + \text{ErbB2} \longrightarrow \text{pErbB12}$$

$$v_{22} = [\text{EGFR\_BTC}] \cdot \text{EGFR\_ErbB2\_BTC\_dimerize} \cdot [\text{ErbB2}]$$

- **Reaction 23:**

$$\text{EGFR\_EGF} + \text{ErbB3\_HRG} \longrightarrow \text{pErbB13}$$

$$v_{23} = [\text{EGFR\_EGF}] \cdot \text{EGFR\_ErbB3\_dimerize} \cdot [\text{ErbB3\_HRG}]$$

- **Reaction 24:**

$$\text{EGFR\_BTC} + \text{ErbB3\_HRG} \longrightarrow \text{pErbB13}$$

$$v_{24} = [\text{EGFR\_BTC}] \cdot \text{EGFR\_ErbB3\_BTC\_dimerize} \cdot [\text{ErbB3\_HRG}]$$

- **Reaction 25:**

$$\text{EGFR\_BTC} + \text{ErbB3} \longrightarrow \text{pErbB13}$$

$$v_{25} = [\text{EGFR\_BTC}] \cdot \text{EGFR\_ErbB3\_dimerize\_noHRG} \cdot [\text{ErbB3}]$$

- **Reaction 26:**

$$\text{ErbB2} + \text{ErbB3\_HRG} \longrightarrow \text{pErbB32}$$

$$v_{26} = [\text{ErbB2}] \cdot \text{ErbB2\_ErbB3\_dimerize} \cdot [\text{ErbB3\_HRG}]$$

- **Reaction 27:**

$$2 \cdot \text{Met\_HGF} \longrightarrow \text{pMetd}$$

$$v_{27} = [\text{Met\_HGF}]^2 \cdot \text{Met\_dimerize}$$

- **Reaction 28:**

$$\text{ErbB3\_HRG} + \text{Met} \longrightarrow \text{pMetErbB3}$$

$$v_{28} = [\text{ErbB3\_HRG}] \cdot [\text{Met}] \cdot \text{Met\_ErbB3\_dimerize}$$

- **Reaction 29:**

$$\text{ErbB3\_HRG} + \text{Met\_HGF} \longrightarrow \text{pMetErbB3}$$

$$v_{29} = [\text{ErbB3\_HRG}] \cdot [\text{Met\_HGF}] \cdot \text{Met\_lig\_ErbB3\_dimerize}$$

- **Reaction 30:**

$$\text{EGFR\_EGF} + \text{Met\_HGF} \longrightarrow \text{pMetEGFR}$$

$$v_{30} = [\text{EGFR\_EGF}] \cdot \text{Met\_EGFR\_dimerize} \cdot [\text{Met\_HGF}]$$

- **Reaction 31:**

$$\text{EGFR\_BTC} + \text{Met\_HGF} \longrightarrow \text{pMetEGFR}$$

$$v_{31} = [\text{EGFR\_BTC}] \cdot \text{Met\_EGFR\_BTC\_dimerize} \cdot [\text{Met\_HGF}]$$

- **Reaction 32:**

$$2 \cdot \text{EGFR} \longrightarrow \text{pEGFRd}$$

$$v_{32} = [\text{EGFR}]^2 \cdot \text{EGFR\_basal\_activation}$$

- **Reaction 33:**

$$2 \cdot \text{ErbB3} \longrightarrow \text{pErbB3d}$$

$$v_{33} = [\text{ErbB3}]^2 \cdot \text{ErbB3\_basal\_activation}$$

- **Reaction 34:**

$$2 \cdot \text{IGF1R} \longrightarrow \text{pIGF1Rd}$$

$$v_{34} = [\text{IGF1R}]^2 \cdot \text{IGF1R\_basal\_activation}$$

- **Reaction 35:**

$$\text{EGFR} + \text{ErbB2} \longrightarrow \text{pErbB12}$$

$$v_{35} = [\text{EGFR}] \cdot \text{EGFR\_ErbB2\_basal\_act} \cdot [\text{ErbB2}]$$

- **Reaction 36:**

$$\text{EGFR} + \text{ErbB3} \longrightarrow \text{pErbB13}$$

$$v_{36} = [\text{EGFR}] \cdot \text{EGFR\_ErbB3\_basal\_act} \cdot [\text{ErbB3}]$$

- **Reaction 37:**

$$\text{ErbB2} + \text{ErbB3} \longrightarrow \text{pErbB32}$$

$$v_{37} = [\text{ErbB2}] \cdot [\text{ErbB3}] \cdot \text{ErbB3\_ErbB2\_basal\_act}$$

- **Reaction 38:**

$$\text{ErbB3} + \text{Met} \longrightarrow \text{pMetErbB3}$$

$$v_{38} = [\text{ErbB3}] \cdot [\text{Met}] \cdot \text{Met\_ErbB3\_basal\_act}$$

- **Reaction 39:**

$$2 \cdot \text{Met} \longrightarrow \text{pMetd}$$

$$v_{39} = [\text{Met}]^2 \cdot \text{Met\_basal\_act}$$

- **Reaction 40:**

$$\text{EGFR} + \text{Met} \longrightarrow \text{pMetEGFR}$$

$v_{40} = [\text{EGFR}] \cdot [\text{Met}] \cdot \text{Met\_EGFR\_basal\_act}$

- **Reaction 41:**

$$\text{pEGFRd} \longrightarrow \text{pEGFRi}$$

$v_{41} = \text{pEGFR\_internalize} \cdot [\text{pEGFRd}]$

- **Reaction 42:**

$$\text{pEGFRi} \longrightarrow \varnothing$$

$v_{42} = \text{pEGFR\_degradation} \cdot [\text{pEGFRi}]$

- **Reaction 43:**

$$\text{RTKph} + \text{pEGFRi} \longrightarrow \text{pEGFRi\_ph}$$

$v_{43} = [\text{RTKph}] \cdot \text{pEGFR\_phosphatase\_binding} \cdot [\text{pEGFRi}]$

- **Reaction 44:**

$$\text{pEGFRi\_ph} \longrightarrow \text{RTKph} + 2 \cdot \text{EGFRi}$$

$v_{44} = \text{pEGFRi\_dephosph} \cdot [\text{pEGFRi\_ph}]$

- **Reaction 45:**

$$\text{EGFRi} \longrightarrow \text{EGFR}$$

$v_{45} = \text{EGFR\_basal\_recycle} \cdot [\text{EGFRi}]$

- **Reaction 46:**

$$\text{pErbB2} \longrightarrow \text{pErbB2i}$$

$v_{46} = [\text{pErbB2}] \cdot \text{pErbB2\_internalize}$

- **Reaction 47:**

$$\text{RTKph} + \text{pErbB2i} \longrightarrow \text{pErbB2i\_ph}$$

$v_{47} = [\text{RTKph}] \cdot [\text{pErbB2i}] \cdot \text{pErbB2i\_phosphatase}$

- **Reaction 48:**

$$\text{pErbB2i\_ph} \longrightarrow \text{RTKph} + 2 \cdot \text{ErbB2i}$$

$v_{48} = \text{pErbB2i\_dephosph} \cdot [\text{pErbB2i\_ph}]$

- **Reaction 49:**

$$\text{pErbB2i} \longrightarrow \varnothing$$

$v_{49} = \text{pErbB2\_degradation} \cdot [\text{pErbB2i}]$

- **Reaction 50:**

$$\text{ErbB2i} \longrightarrow \text{ErbB2}$$

$v_{50} = \text{ErbB2\_recycle} \cdot [\text{ErbB2i}]$

- **Reaction 51:**

$$\text{pErbB3d} \longrightarrow \text{pErbB3i}$$

$v_{51} = \text{pErbB3\_internalize} \cdot [\text{pErbB3d}]$

- **Reaction 52:**

$$\text{pErbB3i} \longrightarrow \varnothing$$

$v_{52} = \text{pErbB3\_degradation} \cdot [\text{pErbB3i}]$

- **Reaction 53:**

$$\text{RTKph} + \text{pErbB3i} \longrightarrow \text{pErbB3i\_ph}$$

$v_{53} = [\text{RTKph}] \cdot [\text{pErbB3i}] \cdot \text{pErbB3i\_phosphatase}$

- **Reaction 54:**

  $$\text{pErbB3i\_ph} \longrightarrow \text{RTKph} + 2 \cdot \text{ErbB3i}$$

  $v_{54} = \text{pErbB3i\_dephosph} \cdot [\text{pErbB3i\_ph}]$

- **Reaction 55:**

  $$\text{ErbB3i} \longrightarrow \text{ErbB3}$$

  $v_{55} = \text{ErbB3\_basal\_recycle} \cdot [\text{ErbB3i}]$

- **Reaction 56:**

  $$\text{pIGF1Rd} \longrightarrow \text{pIGF1Ri}$$

  $v_{56} = \text{pIGF1R\_internalize} \cdot [\text{pIGF1Rd}]$

- **Reaction 57:**

  $$\text{pIGF1Ri} \longrightarrow \varnothing$$

  $v_{57} = \text{pIGF1R\_degradation} \cdot [\text{pIGF1Ri}]$

- **Reaction 58:**

  $$\text{RTKph} + \text{pIGF1Ri} \longrightarrow \text{pIGF1Ri\_ph}$$

  $v_{58} = [\text{RTKph}] \cdot [\text{pIGF1Ri}] \cdot \text{pIGF1Ri\_phosphatase}$

- **Reaction 59:**

  $$\text{pIGF1Ri\_ph} \longrightarrow \text{RTKph} + 2 \cdot \text{IGF1Ri}$$

  $v_{59} = \text{pIGF1Ri\_dephosph} \cdot [\text{pIGF1Ri\_ph}]$

- **Reaction 60:**

  $$\text{IGF1Ri} \longrightarrow \text{IGF1R}$$

  $v_{60} = \text{IGF1R\_basal\_recycle} \cdot [\text{IGF1Ri}]$

- **Reaction 61:**

$$\text{pErbB12} \longrightarrow \text{pErbB12i}$$

$v_{61} = [\text{pErbB12}] \cdot \text{pErbB12\_internalize}$

- **Reaction 62:**

$$\text{pErbB12i} \longrightarrow \varnothing$$

$v_{62} = \text{pErbB12\_degradation} \cdot [\text{pErbB12i}]$

- **Reaction 63:**

$$\text{RTKph} + \text{pErbB12i} \longrightarrow \text{pErbB12i\_ph}$$

$v_{63} = [\text{RTKph}] \cdot [\text{pErbB12i}] \cdot \text{pErbB12i\_phosphatase}$

- **Reaction 64:**

$$\text{pErbB12i\_ph} \longrightarrow \text{RTKph} + \text{EGFRi} + \text{ErbB2i}$$

$v_{64} = \text{pErbB12i\_dephosph} \cdot [\text{pErbB12i\_ph}]$

- **Reaction 65:**

$$\text{pErbB32} \longrightarrow \text{pErbB32i}$$

$v_{65} = [\text{pErbB32}] \cdot \text{pErbB32\_internalize}$

- **Reaction 66:**

$$\text{pErbB32i} \longrightarrow \varnothing$$

$v_{66} = \text{pErbB32\_degradation} \cdot [\text{pErbB32i}]$

- **Reaction 67:**

$$\text{RTKph} + \text{pErbB32i} \longrightarrow \text{pErbB32i\_ph}$$

$v_{67} = [\text{RTKph}] \cdot [\text{pErbB32i}] \cdot \text{pErbB32i\_phosphatase}$

- **Reaction 68:**

  $$\text{pErbB32i\_ph} \longrightarrow \text{RTKph} + \text{ErbB2i} + \text{ErbB3i}$$

  $v_{68} = \text{pErbB32i\_dephosph} \cdot [\text{pErbB32i\_ph}]$

- **Reaction 69:**

  $$\text{pErbB13} \longrightarrow \text{pErbB13i}$$

  $v_{69} = [\text{pErbB13}] \cdot \text{pErbB13\_internalize}$

- **Reaction 70:**

  $$\text{pErbB13i} \longrightarrow \varnothing$$

  $v_{70} = \text{pErbB13\_degradation} \cdot [\text{pErbB13i}]$

- **Reaction 71:**

  $$\text{RTKph} + \text{pErbB13i} \longrightarrow \text{pErbB13i\_ph}$$

  $v_{71} = [\text{RTKph}] \cdot [\text{pErbB13i}] \cdot \text{pErbB13i\_phosphatase}$

- **Reaction 72:**

  $$\text{pErbB13i\_ph} \longrightarrow \text{RTKph} + \text{EGFRi} + \text{ErbB3i}$$

  $v_{72} = \text{pErbB13i\_dephosph} \cdot [\text{pErbB13i\_ph}]$

- **Reaction 73:**

  $$\text{pMetd} \longrightarrow \text{pMeti}$$

  $v_{73} = \text{pMet\_internalize} \cdot [\text{pMetd}]$

- **Reaction 74:**

  $$\text{pMeti} \longrightarrow \varnothing$$

  $v_{74} = \text{pMet\_degradation} \cdot [\text{pMeti}]$

- **Reaction 75:**

$$\text{RTKph} + \text{pMeti} \longrightarrow \text{pMeti\_ph}$$

$$v_{75} = [\text{RTKph}] \cdot [\text{pMeti}] \cdot \text{pMeti\_phosphatase}$$

- **Reaction 76:**

$$\text{pMeti\_ph} \longrightarrow \text{RTKph} + 2 \cdot \text{Meti}$$

$$v_{76} = \text{pMeti\_dephosph} \cdot [\text{pMeti\_ph}]$$

- **Reaction 77:**

$$\text{Meti} \longrightarrow \text{Met}$$

$$v_{77} = \text{Met\_recycle} \cdot [\text{Meti}]$$

- **Reaction 78:**

$$\text{pMetErbB3} \longrightarrow \text{pMetErbB3i}$$

$$v_{78} = [\text{pMetErbB3}] \cdot \text{pMetErbB3\_internalize}$$

- **Reaction 79:**

$$\text{pMetErbB3i} \longrightarrow \varnothing$$

$$v_{79} = \text{pMetErbB3\_degradation} \cdot [\text{pMetErbB3i}]$$

- **Reaction 80:**

$$\text{RTKph} + \text{pMetErbB3i} \longrightarrow \text{pMetErbB3i\_ph}$$

$$v_{80} = [\text{RTKph}] \cdot [\text{pMetErbB3i}] \cdot \text{pMetErbB3i\_phosphatase}$$

- **Reaction 81:**

$$\text{pMetErbB3i\_ph} \longrightarrow \text{RTKph} + \text{ErbB3i} + \text{Meti}$$

$$v_{81} = \text{pMetErbB3i\_dephosph} \cdot [\text{pMetErbB3i\_ph}]$$

- **Reaction 82:**

$$\text{pMetEGFR} \longrightarrow \text{pMetEGFRi}$$

$$v_{82} = [\text{pMetEGFR}] \cdot \text{pMetEGFR\_internalize}$$

- **Reaction 83:**

$$\text{pMetEGFRi} \longrightarrow \varnothing$$

$$v_{83} = \text{pMetEGFR\_degradation} \cdot [\text{pMetEGFRi}]$$

- **Reaction 84:**

$$\text{RTKph} + \text{pMetEGFRi} \longrightarrow \text{pMetEGFRi\_ph}$$

$$v_{84} = [\text{RTKph}] \cdot [\text{pMetEGFRi}] \cdot \text{pMetEGFRi\_phosphatase}$$

- **Reaction 85:**

$$\text{pMetEGFRi\_ph} \longrightarrow \text{RTKph} + \text{EGFRi} + \text{Meti}$$

$$v_{85} = \text{pMetEGFRi\_dephosph} \cdot [\text{pMetEGFRi\_ph}]$$

- **Reaction 86:**

$$\text{MEK} \xrightarrow[\text{pERK}]{\text{pEGFRd}} \text{pMEK}$$

$$v_{86} = \frac{[\text{MEK}] \cdot \text{MEK\_phosphorylation\_pEGFR} \cdot [\text{pEGFRd}]}{\text{feedback\_pAKT} \cdot \text{init\_pAKT} + \text{feedback\_pERK} \cdot [\text{pERK}] + 1}$$

- **Reaction 87:**

$$\text{MEK} \xrightarrow[\text{pERK}]{\text{pErbB12}} \text{pMEK}$$

$$v_{87} = \frac{[\text{MEK}] \cdot \text{MEK\_phosphorylation\_pErbB12} \cdot [\text{pErbB12}]}{\text{feedback\_pAKT} \cdot \text{init\_pAKT} + \text{feedback\_pERK} \cdot [\text{pERK}] + 1}$$

- **Reaction 88:**

$$\text{MEK} \xrightarrow[\text{pERK}]{\text{pErbB13}} \text{pMEK}$$

$$v_{88} = \frac{[\text{MEK}] \cdot \text{MEK\_phosphorylation\_pErbB13} \cdot [\text{pErbB13}]}{\text{feedback\_pAKT} \cdot \text{init\_pAKT} + \text{feedback\_pERK} \cdot [\text{pERK}] + 1}$$

- **Reaction 89:**

$$\text{MEK} \xrightarrow[\text{pERK}]{\text{pErbB32}} \text{pMEK}$$

$$v_{89} = \frac{[\text{MEK}] \cdot \text{MEK\_phosphorylation\_pErbB32} \cdot [\text{pErbB32}]}{\text{feedback\_pAKT} \cdot \text{init\_pAKT} + \text{feedback\_pERK} \cdot [\text{pERK}] + 1}$$

- **Reaction 90:**

$$\text{MEK} \xrightarrow[\text{pERK}]{\text{pMetd}} \text{pMEK}$$

$$v_{90} = \frac{[\text{MEK}] \cdot \text{MEK\_phosphorylation\_pMetd} \cdot [\text{pMetd}]}{\text{feedback\_pAKT} \cdot \text{init\_pAKT} + \text{feedback\_pERK} \cdot [\text{pERK}] + 1}$$

- **Reaction 91:**

$$\text{MEK} \xrightarrow[\text{pERK}]{\text{pMetEGFR}} \text{pMEK}$$

$$v_{91} = \frac{[\text{MEK}] \cdot \text{MEK\_phosphorylation\_pMetEGFR} \cdot [\text{pMetEGFR}]}{\text{feedback\_pAKT} \cdot \text{init\_pAKT} + \text{feedback\_pERK} \cdot [\text{pERK}] + 1}$$

- **Reaction 92:**

$$\text{MEK} \xrightarrow[\text{pERK}]{\text{pIGF1Rd}} \text{pMEK}$$

$$v_{92} = \frac{[\text{MEK}] \cdot \text{MEK\_phosphorylation\_pIGF1R} \cdot [\text{pIGF1Rd}]}{\text{feedback\_pAKT} \cdot \text{init\_pAKT} + \text{feedback\_pERK} \cdot [\text{pERK}] + 1}$$

- **Reaction 93:**

$$\text{MEK} \xrightarrow[\text{pERK}]{\text{pMetErbB3}} \text{pMEK}$$

$$v_{93} = \frac{[\text{MEK}] \cdot \text{MEK\_phosphorylation\_pMetErbB3} \cdot [\text{pMetErbB3}]}{\text{feedback\_pAKT} \cdot \text{init\_pAKT} + \text{feedback\_pERK} \cdot [\text{pERK}] + 1}$$

- **Reaction 94:**

$$\mathrm{MEK} \xrightarrow[\color{red}{\mathrm{pERK}}]{\mathrm{pIGF1Ri}} \mathrm{pMEK}$$

$$v_{94} = \frac{[\mathrm{MEK}] \cdot \mathrm{MEK\_internIGF1R\_effect} \cdot \mathrm{MEK\_phosphorylation\_pIGF1R} \cdot [\mathrm{pIGF1Ri}]}{\mathrm{feedback\_pAKT} \cdot \mathrm{init\_pAKT} + \mathrm{feedback\_pERK} \cdot [\mathrm{pERK}] + 1}$$

- **Reaction 95:**

$$\mathrm{pMEK} \longrightarrow \mathrm{MEK}$$

$$v_{95} = [\mathrm{pMEK}] \cdot \mathrm{pMEK\_dephosphorylation}$$

- **Reaction 96:**

$$\mathrm{ERK} \xrightarrow{\mathrm{pMEK}} \mathrm{pERK}$$

$$v_{96} = [\mathrm{ERK}] \cdot \mathrm{ERK\_phosphorylation\_pMEK} \cdot [\mathrm{pMEK}]$$

- **Reaction 97:**

$$\mathrm{pERK} \longrightarrow \mathrm{ERK}$$

$$v_{97} = [\mathrm{pERK}] \cdot \mathrm{pERK\_dephosphorylation}$$

- **Reaction 98:**

$$\mathrm{AKT} \xrightarrow[\color{red}{\mathrm{pERK,\ pS6K1}}]{\mathrm{pEGFRd}} \mathrm{pAKT}$$

$$v_{98} = \frac{[\mathrm{AKT}] \cdot \mathrm{AKT\_activation\_pEGFR} \cdot [\mathrm{pEGFRd}]}{\mathrm{feedback\_pERK\_on\_AKT} \cdot [\mathrm{pERK}] + \mathrm{feedback\_pS6K1} \cdot [\mathrm{pS6K1}] + 1}$$

- **Reaction 99:**

$$\mathrm{AKT} \xrightarrow[\color{red}{\mathrm{pERK,\ pS6K1}}]{\mathrm{pErbB12}} \mathrm{pAKT}$$

$$v_{99} = \frac{[\mathrm{AKT}] \cdot \mathrm{AKT\_activation\_pErbB12} \cdot [\mathrm{pErbB12}]}{\mathrm{feedback\_pERK\_on\_AKT} \cdot [\mathrm{pERK}] + \mathrm{feedback\_pS6K1} \cdot [\mathrm{pS6K1}] + 1}$$

- **Reaction 100:**

$$\text{AKT} \xrightarrow[\text{pERK, pS6K1}]{\text{pErbB13}} \text{pAKT}$$

$$v_{100} = \frac{[\text{AKT}] \cdot \text{AKT\_activation\_pErbB13} \cdot [\text{pErbB13}]}{\text{feedback\_pERK\_on\_AKT} \cdot [\text{pERK}] + \text{feedback\_pS6K1} \cdot [\text{pS6K1}] + 1}$$

- **Reaction 101:**

$$\text{AKT} \xrightarrow[\text{pERK, pS6K1}]{\text{pErbB32}} \text{pAKT}$$

$$v_{101} = \frac{[\text{AKT}] \cdot \text{AKT\_activation\_pErbB32} \cdot [\text{pErbB32}]}{\text{feedback\_pERK\_on\_AKT} \cdot [\text{pERK}] + \text{feedback\_pS6K1} \cdot [\text{pS6K1}] + 1}$$

- **Reaction 102:**

$$\text{AKT} \xrightarrow[\text{pERK, pS6K1}]{\text{pMetEGFR}} \text{pAKT}$$

$$v_{102} = \frac{[\text{AKT}] \cdot \text{AKT\_activation\_pMetEGFR} \cdot [\text{pMetEGFR}]}{\text{feedback\_pERK\_on\_AKT} \cdot [\text{pERK}] + \text{feedback\_pS6K1} \cdot [\text{pS6K1}] + 1}$$

- **Reaction 103:**

$$\text{AKT} \xrightarrow[\text{pERK, pS6K1}]{\text{pMetd}} \text{pAKT}$$

$$v_{103} = \frac{[\text{AKT}] \cdot \text{AKT\_activation\_pMetd} \cdot [\text{pMetd}]}{\text{feedback\_pERK\_on\_AKT} \cdot [\text{pERK}] + \text{feedback\_pS6K1} \cdot [\text{pS6K1}] + 1}$$

- **Reaction 104:**

$$\text{AKT} \xrightarrow[\text{pERK, pS6K1}]{\text{pIGF1Rd}} \text{pAKT}$$

$$v_{104} = \frac{[\text{AKT}] \cdot \text{AKT\_activation\_pIGF1R} \cdot [\text{pIGF1Rd}]}{\text{feedback\_pERK\_on\_AKT} \cdot [\text{pERK}] + \text{feedback\_pS6K1} \cdot [\text{pS6K1}] + 1}$$

- **Reaction 105:**

$$\text{AKT} \xrightarrow[\text{pERK, pS6K1}]{\text{pMetErbB3}} \text{pAKT}$$

$$v_{105} = \frac{[\text{AKT}] \cdot \text{AKT\_activation\_pMetErbB3} \cdot [\text{pMetErbB3}]}{\text{feedback\_pERK\_on\_AKT} \cdot [\text{pERK}] + \text{feedback\_pS6K1} \cdot [\text{pS6K1}] + 1}$$

- **Reaction 106:**

$$\mathrm{AKT} \xrightarrow[\text{pERK, pS6K1}]{\text{pIGF1Ri}} \mathrm{pAKT}$$

$$v_{106} = \frac{[\mathrm{AKT}] \cdot \mathrm{AKT\_activation\_pIGF1R} \cdot \mathrm{AKT\_internIGF1R\_effect} \cdot [\mathrm{pIGF1Ri}]}{\mathrm{feedback\_pERK\_on\_AKT} \cdot [\mathrm{pERK}] + \mathrm{feedback\_pS6K1} \cdot [\mathrm{pS6K1}] + 1}$$

- **Reaction 107:**

$$\mathrm{pAKT} \longrightarrow \mathrm{AKT}$$

$$v_{107} = [\mathrm{pAKT}] \cdot \mathrm{pAKT\_deactivation}$$

- **Reaction 108:**

$$\mathrm{S6K1} \xrightarrow{\text{pAKT}} \mathrm{pS6K1}$$

$$v_{108} = [\mathrm{S6K1}] \cdot \mathrm{S6K1\_phosphorylation\_pAKT} \cdot [\mathrm{pAKT}]$$

- **Reaction 109:**

$$\mathrm{S6K1} \xrightarrow{\text{pERK}} \mathrm{pS6K1}$$

$$v_{109} = [\mathrm{S6K1}] \cdot \mathrm{S6K1\_phosphorylation\_pERK} \cdot [\mathrm{pERK}]$$

- **Reaction 110:**

$$\mathrm{pS6K1} \longrightarrow \mathrm{S6K1}$$

$$v_{110} = [\mathrm{pS6K1}] \cdot \mathrm{pS6K1\_dephosphorylation}$$

- **Reaction 111:**

$$\mathrm{S6} \xrightarrow{\text{pS6K1}} \mathrm{pS6}$$

$$v_{111} = [\mathrm{S6}] \cdot \mathrm{S6\_phosphorylation\_pS6K1} \cdot [\mathrm{pS6K1}]$$

- **Reaction 112:**

$$\mathrm{S6} \xrightarrow{\text{pERK}} \mathrm{pS6}$$

$$v_{112} = [\mathrm{S6}] \cdot \mathrm{S6\_phosphorylation\_pERK} \cdot [\mathrm{pERK}]$$

- **Reaction 113:**

$$\text{pS6} \longrightarrow \text{S6}$$

$$v_{113} = [\text{pS6}] \cdot \text{pS6\_dephosphorylation}$$

1.5 Observables

The model contains 19 standard observables listed in table 2.

Observable		Equations
FACS_EGFR [au]	y	$\log_{10}$([facs_der_egfr])
	σ	sd_FACS_EGFR
FACS_ErbB2 [au]	y	$\log_{10}$([facs_der_erbb2])
	σ	sd_FACS_ErbB2
FACS$_{E}rbB3[au]$	y	$\log_{10}$([facs_der_erbb3])
	σ	sd_FACS_ErbB3
FACS_IGF1R [au]	y	$\log_{10}$([facs_der_igf1r])
	σ	sd_FACS_IGF1R
FACS_Met [au]	y	$\log_{10}$([facs_der_met])
	σ	sd_FACS_Met
tEGFR_au [au]	y	$\log_{10}$(offset_tEGFR_Cellline + scale_tEGFR_Cellline · ([EGFR] + [EGFR_BTC] + [EGFR_EGF] + [EGFRi] + 2 · [pEGFRd] + 2 · [pEGFRi] + 2 · [pEGFRi_ph] + [pErbB12] + [pErbB12i] + [pErbB12i_ph] + [pErbB13] + [pErbB13i] + [pErbB13i_ph] +[pMetEGFR] + [pMetEGFRi] + [pMetEGFRi_ph])
	σ	sd_tEGFR_au_Cellline
tErbB2_au [au]	y	$\log_{10}$(offset_tErbB2_Cellline + scale_tErbB2_Cellline · ([ErbB2] + [ErbB2i] + [pErbB12] + [pErbB12i] + [pErbB12i_ph] + 2 · [pErbB2] + 2 · [pErbB2i] + 2 · [pErbB2i_ph] + [pErbB32] + [pErbB32i] + [pErbB32i_ph]))
	σ	sd_tErbB2_au_Cellline
tErbB3_au [au]	y	$\log_{10}$(offset_tErbB3_Cellline + scale_tErbB3_Cellline · ([ErbB3] + [ErbB3_HRG] + [ErbB3i] + [pErbB13] + [pErbB13i] + [pErbB13i_ph] + [pErbB32] + [pErbB32i] + [pErbB32i_ph] + 2 · [pErbB3d] + 2 · [pErbB3i] + 2 · [pErbB3i_ph] +[pMetErbB3] + [pMetErbB3i] + [pMetErbB3i_ph])
	σ	sd_tErbB3_au_Cellline
tIGF1R_au [au]	y	$\log_{10}$(offset_tIGF1R_Cellline + scale_tIGF1R_Cellline · ([IGF1R] + [IGF1R_IGF1] + [IGF1Ri] + 2 · [pIGF1Rd] + 2 · [pIGF1Ri] + 2 · [pIGF1Ri_ph]))
	σ	sd_tIGF1R_au_Cellline
pEGFR_au [au]	y	$\log_{10}$(offset_pEGFR_Cellline + scale_pEGFR_Cellline · (2 · [pEGFRd] + 2 · [pEGFRi] + 2 · [pEGFRi_ph] + [pErbB12] + [pErbB12i] + [pErbB12i_ph] + [pErbB13] + [pErbB13i] + [pErbB13i_ph] + [pMetEGFR] + [pMetEGFRi] + [pMetEGFRi_ph]))
	σ	sd_pEGFR_au_Cellline
pErbB2_au [au]	y	$\log_{10}$(offset_pErbB2_Cellline + scale_pErbB2_Cellline · ([pErbB12] + [pErbB12i] + [pErbB12i_ph] + 2 · [pErbB2] + 2 · [pErbB2i] + 2 · [pErbB2i_ph] + [pErbB32] + [pErbB32i] + [pErbB32i_ph]))
	σ	sd_pErbB2_au_Cellline
pErbB3_au [au]	y	$\log_{10}$(offset_pErbB3_Cellline + scale_pErbB3_Cellline · ([pErbB13] + [pErbB13i] + [pErbB13i_ph] + [pErbB32] + [pErbB32i] + [pErbB32i_ph] + 2 · [pErbB3d] + 2 · [pErbB3i] + 2 · [pErbB3i_ph] + [pMetErbB3] + [pMetErbB3i] + [pMetErbB3i_ph]))
	σ	sd_pErbB3_au_Cellline
pIGF1R_au [au]	y	$\log_{10}$(offset_pIGF1R_Cellline + scale_pIGF1R_Cellline · (2 · [pIGF1Rd] + 2 · [pIGF1Ri] + 2 · [pIGF1Ri_ph]))
	σ	sd_pIGF1R_au_Cellline
pMet_au [au]	y	$\log_{10}$(offset_pMet_Cellline + scale_pMet_Cellline · ([pMetEGFR] + [pMetEGFRi] + [pMetEGFRi_ph] + [pMetErbB3] + [pMetErbB3i] + [pMetErbB3i_ph] + 2 · [pMetd] + 2 · [pMeti] + 2 · [pMeti_ph]))
	σ	sd_pMet_au_Cellline
pMEK_au [au]	y	$\log_{10}$(offset_pMEK_Cellline + [pMEK] · scale_pMEK_Cellline)
	σ	sd_pMEK_au_Cellline
pERK_au [au]	y	$\log_{10}$(offset_pERK_Cellline + [pERK] · scale_pERK_Cellline)
	σ	sd_pERK_au_Cellline
pAKT_au [au]	y	$\log_{10}$(offset_pAKT_Cellline + [pAKT] · scale_pAKT_Cellline)
	σ	sd_pAKT_au_Cellline
pS6K1_au [au]	y	$\log_{10}$(offset_pS6K1_Cellline + [pS6K1] · scale_pS6K1_Cellline)
	σ	sd_pS6K1_au_Cellline
pS6_au [au]	y	$\log_{10}$(offset_pS6_Cellline + [pS6] · scale_pS6_Cellline)
	σ	sd_pS6_au_Cellline

Suppl. Table 2: Model observables and error models

1.6 Dynamic parameters

In total 442 parameters are estimated from the experimental data, yielding a value of the objective function $\chi^2 = 30977.2$ for a total of 10428 data points. The model parameters were estimated by maximum likelihood estimation applying the MATLAB lsqnonlin algorithm. The model parameters which influence system dynamics are listed in Table 3. Parameters highlighted in red color indicate parameter values close to their bounds. An extensive list of all the estimated parameters (including all observational and error model parameters) is given in section 1.17.

	name	θ_{min}	$\hat{\theta}$	θ_{max}	log	non-log $\hat{\theta}$	fitted
1	AKT_activation_pEGFR	-5	-5.0000	+3	1	$+1.00 \cdot 10^{-05}$	1
2	AKT_activation_pErbB12	-5	-1.1940	+3	1	$+6.40 \cdot 10^{-02}$	1
3	AKT_activation_pErbB13	-5	+1.1178	+3	1	$+1.31 \cdot 10^{+01}$	1
4	AKT_activation_pErbB32	-5	-0.2513	+3	1	$+5.61 \cdot 10^{-01}$	1
5	AKT_activation_pIGF1R	-5	-0.1641	+3	1	$+6.85 \cdot 10^{-01}$	1
6	AKT_activation_pMetEGFR	-5	-5.0000	+3	1	$+1.00 \cdot 10^{-05}$	1
7	AKT_activation_pMetErbB3	-5	-1.4321	+3	1	$+3.70 \cdot 10^{-02}$	1
8	AKT_activation_pMetd	-5	-0.0479	+3	1	$+8.96 \cdot 10^{-01}$	1
9	AKT_internIGF1R_effect	-5	-4.9907	+3	1	$+1.02 \cdot 10^{-05}$	1
10	EGFR_BTC_binding	-5	-4.6498	+3	1	$+2.24 \cdot 10^{-05}$	1
11	EGFR_BTC_dimerize	-5	+3.0000	+3	1	$+1.00 \cdot 10^{+03}$	1
12	EGFR_ErbB2_BTC_dimerize	-5	+0.2072	+3	1	$+1.61 \cdot 10^{+00}$	1
13	EGFR_ErbB2_basal_act	-5	-5.0000	+6	1	$+1.00 \cdot 10^{-05}$	1
14	EGFR_ErbB2_dimerize	-5	-1.8041	+3	1	$+1.57 \cdot 10^{-02}$	1
15	EGFR_ErbB3_BTC_dimerize	-5	-1.4539	+3	1	$+3.52 \cdot 10^{-02}$	1
16	EGFR_ErbB3_basal_act	-5	-3.1243	+6	1	$+7.51 \cdot 10^{-04}$	1
17	EGFR_ErbB3_dimerize	-5	-2.9275	+3	1	$+1.18 \cdot 10^{-03}$	1
18	EGFR_ErbB3_dimerize_noHRG	-5	-5.0000	+3	1	$+1.00 \cdot 10^{-05}$	1
19	EGFR_basal_activation	-5	-5.0000	+6	1	$+1.00 \cdot 10^{-05}$	1
20	EGFR_basal_recycle	-5	+5.7152	+6	1	$+5.19 \cdot 10^{+05}$	1
21	EGFR_dimerize	-5	-1.2005	+3	1	$+6.30 \cdot 10^{-02}$	1
22	EGFR_lig_binding	-5	-4.7227	+3	1	$+1.89 \cdot 10^{-05}$	1
23	EGF_kD	-5	+0.0000	+3	1	$+1.00 \cdot 10^{+00}$	2
24	ERK_phosphorylation_pMEK	-5	-3.5879	+3	1	$+2.58 \cdot 10^{-04}$	1
25	ErbB2_ErbB3_dimerize	-5	-1.5192	+3	1	$+3.03 \cdot 10^{-02}$	1
26	ErbB2_dimerize	-5	-2.1680	+3	1	$+6.79 \cdot 10^{-03}$	1
27	ErbB2_recycle	-5	-2.1824	+3	1	$+6.57 \cdot 10^{-03}$	1
28	ErbB3_ErbB2_basal_act	-5	-5.0000	+6	1	$+1.00 \cdot 10^{-05}$	1
29	ErbB3_basal_activation	-5	-1.5331	+6	1	$+2.93 \cdot 10^{-02}$	1
30	ErbB3_basal_recycle	-5	-0.1327	+6	1	$+7.37 \cdot 10^{-01}$	1
31	ErbB3_dimerize	-5	-1.3508	+3	1	$+4.46 \cdot 10^{-02}$	1
32	ErbB3_lig_binding	-5	-4.2430	+3	1	$+5.71 \cdot 10^{-05}$	1
33	HGF_kD	-5	-0.5229	+3	1	$+3.00 \cdot 10^{-01}$	2
34	HRG_kD	-5	-1.3010	+3	1	$+5.00 \cdot 10^{-02}$	2
35	IGF1R_basal_activation	-5	-2.9317	+3	1	$+1.17 \cdot 10^{-03}$	1
36	IGF1R_basal_recycle	-5	+3.0000	+3	1	$+1.00 \cdot 10^{+03}$	1
37	IGF1R_dimerize	-5	+1.2337	+3	1	$+1.71 \cdot 10^{+01}$	1
38	IGF1R_lig_binding	-5	-2.8155	+3	1	$+1.53 \cdot 10^{-03}$	1
39	IGF1_kD	-5	-0.5229	+3	1	$+3.00 \cdot 10^{-01}$	2
40	MEK_internIGF1R_effect	-5	-5.0000	+3	1	$+1.00 \cdot 10^{-05}$	1
41	MEK_phosphorylation_pEGFR	-5	-5.0000	+3	1	$+1.00 \cdot 10^{-05}$	1
42	MEK_phosphorylation_pErbB12	-5	-0.5625	+3	1	$+2.74 \cdot 10^{-01}$	1
43	MEK_phosphorylation_pErbB13	-5	-5.0000	+3	1	$+1.00 \cdot 10^{-05}$	1
44	MEK_phosphorylation_pErbB32	-5	-1.2178	+3	1	$+6.06 \cdot 10^{-02}$	1
45	MEK_phosphorylation_pIGF1R	-5	-1.5236	+3	1	$+2.99 \cdot 10^{-02}$	1
46	MEK_phosphorylation_pMetEGFR	-5	-5.0000	+3	1	$+1.00 \cdot 10^{-05}$	1
47	MEK_phosphorylation_pMetErbB3	-5	-1.4164	+3	1	$+3.83 \cdot 10^{-02}$	1
48	MEK_phosphorylation_pMetd	-5	+0.2741	+3	1	$+1.88 \cdot 10^{+00}$	1
49	Met_EGFR_BTC_dimerize	-5	-1.9539	+3	1	$+1.11 \cdot 10^{-02}$	1
50	Met_EGFR_basal_act	-5	-4.7581	+6	1	$+1.75 \cdot 10^{-05}$	1
51	Met_EGFR_dimerize	-5	-3.2898	+3	1	$+5.13 \cdot 10^{-04}$	1
52	Met_ErbB3_basal_act	-5	+0.5177	+6	1	$+3.29 \cdot 10^{+00}$	1
53	Met_ErbB3_dimerize	-5	-1.4308	+3	1	$+3.71 \cdot 10^{-02}$	1
54	Met_basal_act	-5	-5.0000	+6	1	$+1.00 \cdot 10^{-05}$	1
55	Met_dimerize	-5	-2.0378	+3	1	$+9.17 \cdot 10^{-03}$	1
56	Met_lig_ErbB3_dimerize	-5	+2.7536	+3	1	$+5.67 \cdot 10^{+02}$	1
57	Met_lig_binding	-5	-2.3452	+3	1	$+4.52 \cdot 10^{-03}$	1
58	Met_recycle	-5	-0.2656	+3	1	$+5.42 \cdot 10^{-01}$	1
59	S6K1_phosphorylation_pAKT	-5	-0.6024	+3	1	$+2.50 \cdot 10^{-01}$	1
60	S6K1_phosphorylation_pERK	-5	-4.9718	+3	1	$+1.07 \cdot 10^{-05}$	1
61	S6_phosphorylation_pERK	-5	-4.9987	+3	1	$+1.00 \cdot 10^{-05}$	1
62	S6_phosphorylation_pS6K1	-5	-2.0671	+3	1	$+8.57 \cdot 10^{-03}$	1
63	feedback_pAKT	-5	-4.9754	+3	1	$+1.06 \cdot 10^{-05}$	1
64	feedback_pERK	-5	+3.0000	+3	1	$+1.00 \cdot 10^{+03}$	1
65	feedback_pERK_on_AKT	-5	-4.9989	+3	1	$+1.00 \cdot 10^{-05}$	1

66	feedback_pS6K1	-5	-4.6796	+3	1	$+2.09 \cdot 10^{-05}$	1
67	init_AKT	-5	+0.4327	+3	1	$+2.71 \cdot 10^{+00}$	1
68	init_EGFR	-5	+1.2519	+3	1	$+1.79 \cdot 10^{+01}$	1
69	init_EGFR_BTC	-5	+0.0000	+3	0	$+0.00 \cdot 10^{+00}$	2
70	init_EGFR_EGF	-5	+0.0000	+3	0	$+0.00 \cdot 10^{+00}$	2
71	init_ErbB2	-5	+0.7560	+3	1	$+5.70 \cdot 10^{+00}$	1
72	init_ErbB3	-5	+0.3944	+3	1	$+2.48 \cdot 10^{+00}$	1
73	init_ErbB3_HRG	-5	+0.0000	+3	0	$+0.00 \cdot 10^{+00}$	2
74	init_IGF1R	-5	+0.6753	+3	1	$+4.73 \cdot 10^{+00}$	1
75	init_IGF1R_IGF1	-5	+0.0000	+3	0	$+0.00 \cdot 10^{+00}$	2
76	init_MEK	-5	+0.6275	+3	1	$+4.24 \cdot 10^{+00}$	1
77	init_Met	-5	+0.8978	+3	1	$+7.90 \cdot 10^{+00}$	1
78	init_Met_HGF	-5	+0.0000	+3	0	$+0.00 \cdot 10^{+00}$	2
79	init_RTKph	-5	-0.2084	+3	1	$+6.19 \cdot 10^{-01}$	1
80	init_S6	-5	+2.1629	+3	1	$+1.46 \cdot 10^{+02}$	1
81	init_pERK	-5	-0.4759	+3	1	$+3.34 \cdot 10^{-01}$	1
82	init_pS6K1	-5	-2.9054	+3	1	$+1.24 \cdot 10^{-03}$	1
251	pAKT_deactivation	-5	-0.5186	+3	1	$+3.03 \cdot 10^{-01}$	1
252	pEGFR_degradation	-5	-5.0000	+3	1	$+1.00 \cdot 10^{-05}$	1
253	pEGFR_internalize	-5	+0.7946	+3	1	$+6.23 \cdot 10^{+00}$	1
254	pEGFR_phosphatase_binding	-5	+2.2663	+3	1	$+1.85 \cdot 10^{+02}$	1
255	pEGFRi_dephosph	-5	+1.3369	+3	1	$+2.17 \cdot 10^{+01}$	1
256	pERK_dephosphorylation	-5	-0.2655	+3	1	$+5.43 \cdot 10^{-01}$	1
257	pErbB12_degradation	-5	-0.7403	+3	1	$+1.82 \cdot 10^{-01}$	1
258	pErbB12_internalize	-5	+0.2777	+3	1	$+1.90 \cdot 10^{+00}$	1
259	pErbB12i_dephosph	-5	+3.0000	+3	1	$+1.00 \cdot 10^{+03}$	1
260	pErbB12i_phosphatase	-5	-0.0730	+3	1	$+8.45 \cdot 10^{-01}$	1
261	pErbB13_degradation	-5	+1.6965	+3	1	$+4.97 \cdot 10^{+01}$	1
262	pErbB13_internalize	-5	+3.0000	+3	1	$+1.00 \cdot 10^{+03}$	1
263	pErbB13i_dephosph	-5	+1.7665	+3	1	$+5.84 \cdot 10^{+01}$	1
264	pErbB13i_phosphatase	-5	-4.7972	+3	1	$+1.60 \cdot 10^{-05}$	1
265	pErbB2_degradation	-5	+2.5034	+3	1	$+3.19 \cdot 10^{+02}$	1
266	pErbB2_internalize	-5	+3.0000	+3	1	$+1.00 \cdot 10^{+03}$	1
267	pErbB2i_dephosph	-5	+0.9161	+3	1	$+8.24 \cdot 10^{+00}$	1
268	pErbB2i_phosphatase	-5	+3.0000	+3	1	$+1.00 \cdot 10^{+03}$	1
269	pErbB32_degradation	-5	-0.2414	+3	1	$+5.74 \cdot 10^{-01}$	1
270	pErbB32_internalize	-5	+0.0363	+3	1	$+1.09 \cdot 10^{+00}$	1
271	pErbB32i_dephosph	-5	-1.9237	+3	1	$+1.19 \cdot 10^{-02}$	1
272	pErbB32i_phosphatase	-5	-1.3117	+3	1	$+4.88 \cdot 10^{-02}$	1
273	pErbB3_degradation	-5	-0.0417	+3	1	$+9.08 \cdot 10^{-01}$	1
274	pErbB3_internalize	-5	+3.0000	+3	1	$+1.00 \cdot 10^{+03}$	1
275	pErbB3i_dephosph	-5	+1.2126	+3	1	$+1.63 \cdot 10^{+01}$	1
276	pErbB3i_phosphatase	-5	+1.9148	+3	1	$+8.22 \cdot 10^{+01}$	1
277	pIGF1R_degradation	-5	-5.0000	+3	1	$+1.00 \cdot 10^{-05}$	1
278	pIGF1R_internalize	-5	+3.0000	+3	1	$+1.00 \cdot 10^{+03}$	1
279	pIGF1Ri_dephosph	-5	+2.5207	+3	1	$+3.32 \cdot 10^{+02}$	1
280	pIGF1Ri_phosphatase	-5	+3.0000	+3	1	$+1.00 \cdot 10^{+03}$	1
281	pMEK_dephosphorylation	-5	-0.4839	+3	1	$+3.28 \cdot 10^{-01}$	1
282	pMetEGFR_degradation	-5	+0.1539	+3	1	$+1.43 \cdot 10^{+00}$	1
283	pMetEGFR_internalize	-5	+0.1229	+3	1	$+1.33 \cdot 10^{+00}$	1
284	pMetEGFRi_dephosph	-5	-0.2978	+3	1	$+5.04 \cdot 10^{-01}$	1
285	pMetEGFRi_phosphatase	-5	-4.4470	+3	1	$+3.57 \cdot 10^{-05}$	1
286	pMetErbB3_degradation	-5	+2.9929	+3	1	$+9.84 \cdot 10^{+02}$	1
287	pMetErbB3_internalize	-5	+3.0000	+3	1	$+1.00 \cdot 10^{+03}$	1
288	pMetErbB3i_dephosph	-5	+3.0000	+3	1	$+1.00 \cdot 10^{+03}$	1
289	pMetErbB3i_phosphatase	-5	+3.0000	+3	1	$+1.00 \cdot 10^{+03}$	1
290	pMet_degradation	-5	+0.2802	+3	1	$+1.91 \cdot 10^{+00}$	1
291	pMet_internalize	-5	+3.0000	+3	1	$+1.00 \cdot 10^{+03}$	1
292	pMeti_dephosph	-5	+1.1134	+3	1	$+1.30 \cdot 10^{+01}$	1
293	pMeti_phosphatase	-5	+3.0000	+3	1	$+1.00 \cdot 10^{+03}$	1
294	pS6K1_dephosphorylation	-5	-1.9859	+3	1	$+1.03 \cdot 10^{-02}$	1
295	pS6_dephosphorylation	-5	-0.9252	+3	1	$+1.19 \cdot 10^{-01}$	1
296	relto_A431_init_EGFR	-4	+0.9838	+4	1	$+9.63 \cdot 10^{+00}$	1
297	relto_A431_init_ErbB2	-4	+0.0013	+4	1	$+1.00 \cdot 10^{+00}$	1
298	relto_A431_init_ErbB3	-4	-0.0403	+4	1	$+9.11 \cdot 10^{-01}$	1

299	relto_A431_init_IGF1R	-4	+0.4236	+4	1	$+2.65 \cdot 10^{+00}$	1
300	relto_A431_init_Met	-4	-0.6500	+4	1	$+2.24 \cdot 10^{-01}$	1
301	relto_ACHN_init_EGFR	-4	+0.7324	+4	1	$+5.40 \cdot 10^{+00}$	1
302	relto_ACHN_init_ErbB2	-4	-0.1347	+4	1	$+7.33 \cdot 10^{-01}$	1
303	relto_ACHN_init_ErbB3	-4	-0.3509	+4	1	$+4.46 \cdot 10^{-01}$	1
304	relto_ACHN_init_IGF1R	-4	-0.1530	+4	1	$+7.03 \cdot 10^{-01}$	1
305	relto_ACHN_init_Met	-4	+0.1113	+4	1	$+1.29 \cdot 10^{+00}$	1
306	relto_ADRr_init_EGFR	-4	+0.0119	+4	1	$+1.03 \cdot 10^{+00}$	1
307	relto_ADRr_init_ErbB2	-4	+0.0311	+4	1	$+1.07 \cdot 10^{+00}$	1
308	relto_ADRr_init_ErbB3	-4	-0.2414	+4	1	$+5.74 \cdot 10^{-01}$	1
309	relto_ADRr_init_IGF1R	-4	+0.3059	+4	1	$+2.02 \cdot 10^{+00}$	1
310	relto_ADRr_init_Met	-4	-0.3171	+4	1	$+4.82 \cdot 10^{-01}$	1
311	relto_BT20_init_EGFR	-4	+1.0201	+4	1	$+1.05 \cdot 10^{+01}$	1
312	relto_BT20_init_ErbB2	-4	+0.1754	+4	1	$+1.50 \cdot 10^{+00}$	1
313	relto_BT20_init_ErbB3	-4	+0.2661	+4	1	$+1.85 \cdot 10^{+00}$	1
314	relto_BT20_init_IGF1R	-4	+0.5507	+4	1	$+3.55 \cdot 10^{+00}$	1
315	relto_BT20_init_Met	-4	-0.4177	+4	1	$+3.82 \cdot 10^{-01}$	1
316	relto_IGROV_init_EGFR	-4	-0.0264	+4	1	$+9.41 \cdot 10^{-01}$	1
317	relto_IGROV_init_ErbB2	-4	+0.6286	+4	1	$+4.25 \cdot 10^{+00}$	1
318	relto_IGROV_init_ErbB3	-4	-0.2399	+4	1	$+5.76 \cdot 10^{-01}$	1
319	relto_IGROV_init_IGF1R	-4	-0.7987	+4	1	$+1.59 \cdot 10^{-01}$	1
320	relto_IGROV_init_Met	-4	-0.1926	+4	1	$+6.42 \cdot 10^{-01}$	1
321	relto_init_EGFR	-4	+0.2627	+4	1	$+1.83 \cdot 10^{+00}$	1
322	relto_init_ErbB2	-4	-0.2169	+4	1	$+6.07 \cdot 10^{-01}$	1
323	relto_init_ErbB3	-4	+0.0165	+4	1	$+1.04 \cdot 10^{+00}$	1
324	relto_init_IGF1R	-4	+0.1713	+4	1	$+1.48 \cdot 10^{+00}$	1
325	relto_init_Met	-4	+0.2563	+4	1	$+1.80 \cdot 10^{+00}$	1
326	scale_Ligand	+1	+4.5776	+6	1	$+3.78 \cdot 10^{+04}$	1

Suppl. Table 3: Estimated dynamic parameter values
$\hat{\theta}$ indicates the estimated value of the parameters. θ_{min} and θ_{max} indicate the upper and lower bounds for the parameters. The log-column indicates if the value of a parameter was log-transformed. If log = 1 the non-log-column indicates the non-logarithmic value of the estimate. The fitted-column indicates if the parameter value was estimated (1), was temporarily fixed (0) or if its value was fixed to a constant value (2).

1.7 Experimental data and model fits

The model observables and the experimental data is shown in Figures 11 to 40. In each figure, the observables are displayed as solid lines and the error model that describes the measurement noise is indicated by shades. Data is shown as dots in the color of the respective ligand condition. The agreement of the model observables and the experimental data yields a value of the objective function $\chi^2 = 30977.2$ for 10428 data points in this data set.

1.8 Cell line H322M

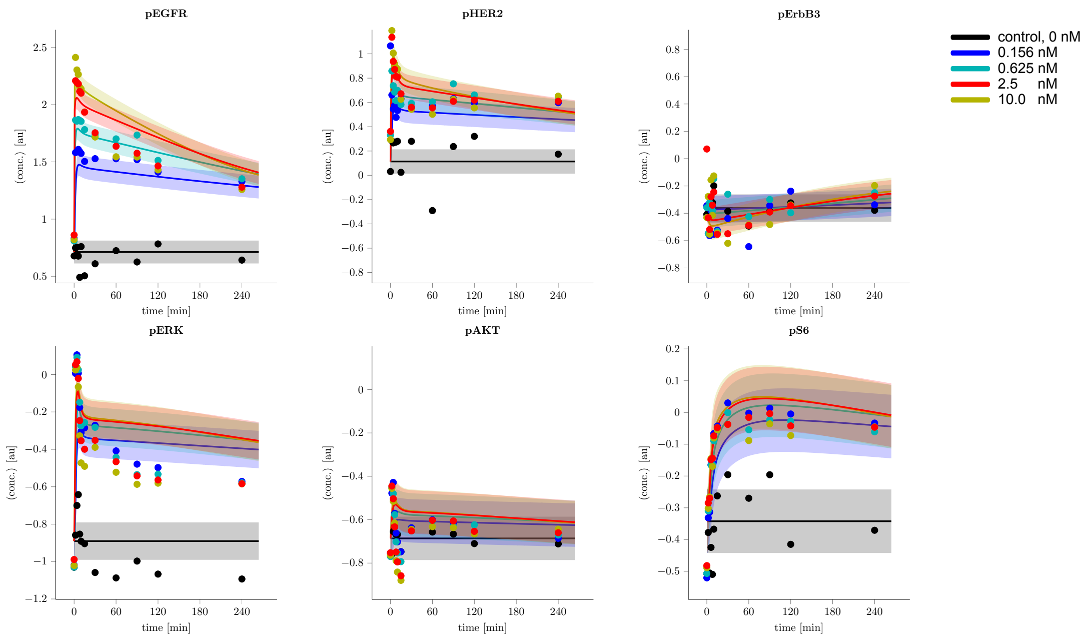

Suppl. Figure 11: Model trajectories for cell line H322M after EGF stimulation.

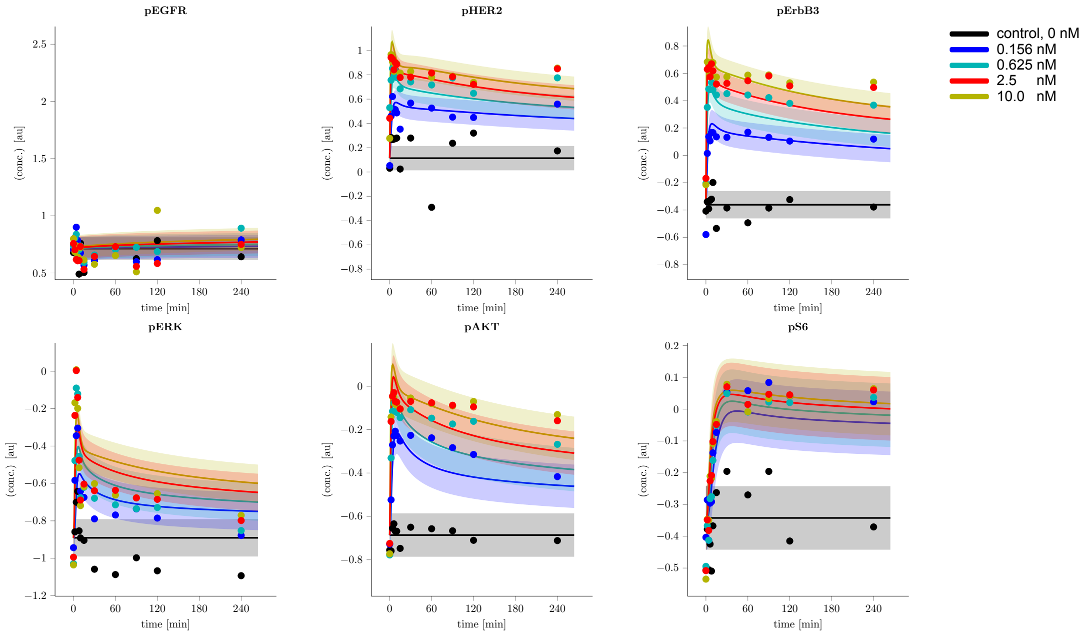

Suppl. Figure 12: Model trajectories for cell line H322M after HRG stimulation.

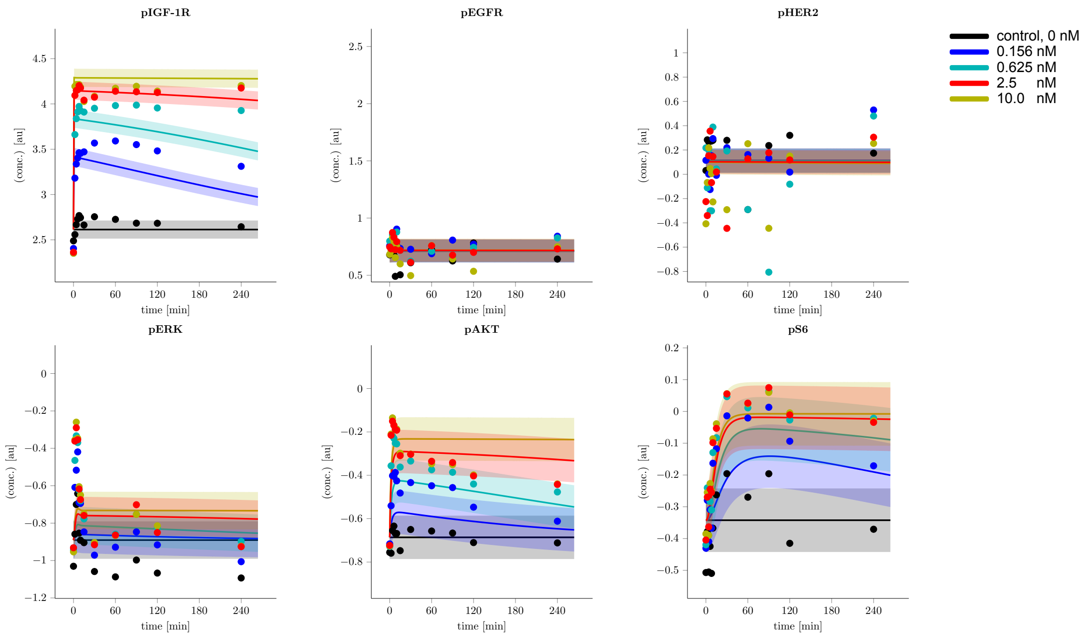

Suppl. Figure 13: Model trajectories for cell line H322M after IGF-1 stimulation.

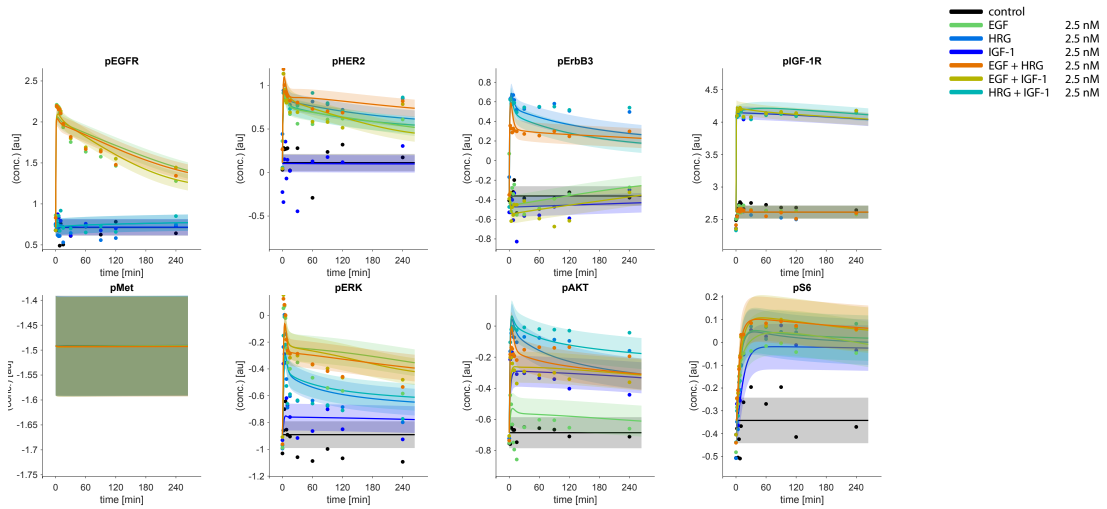

Suppl. Figure 14: Model trajectories for cell line H322M after ligand co-stimulations.

1.9 Cell line BxPc-3

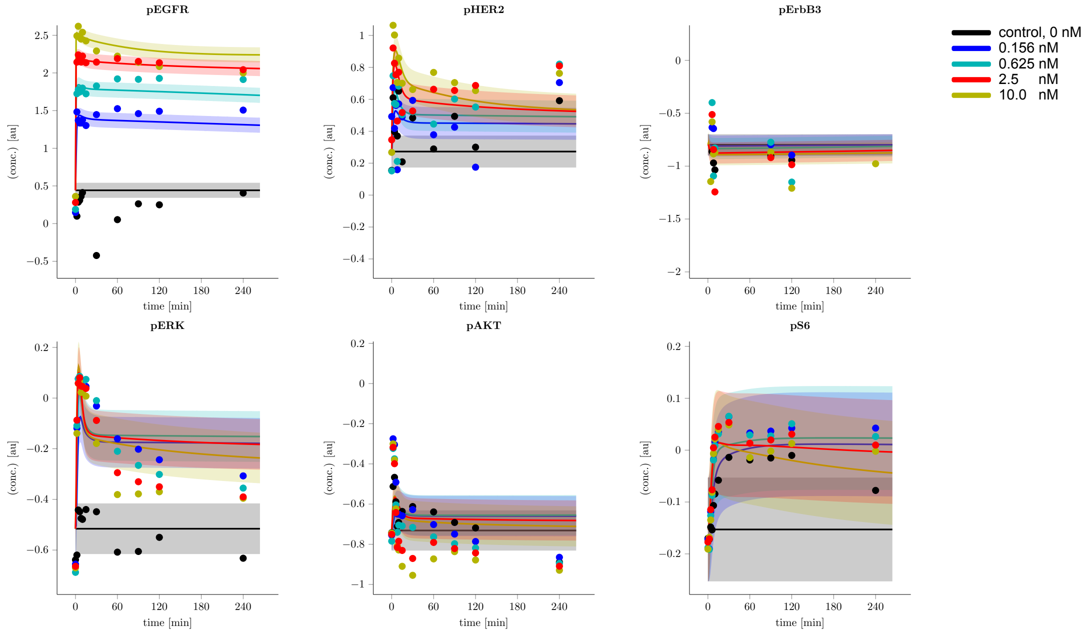

Suppl. Figure 15: Model trajectories for cell line BxPc-3 after EGF stimulation.

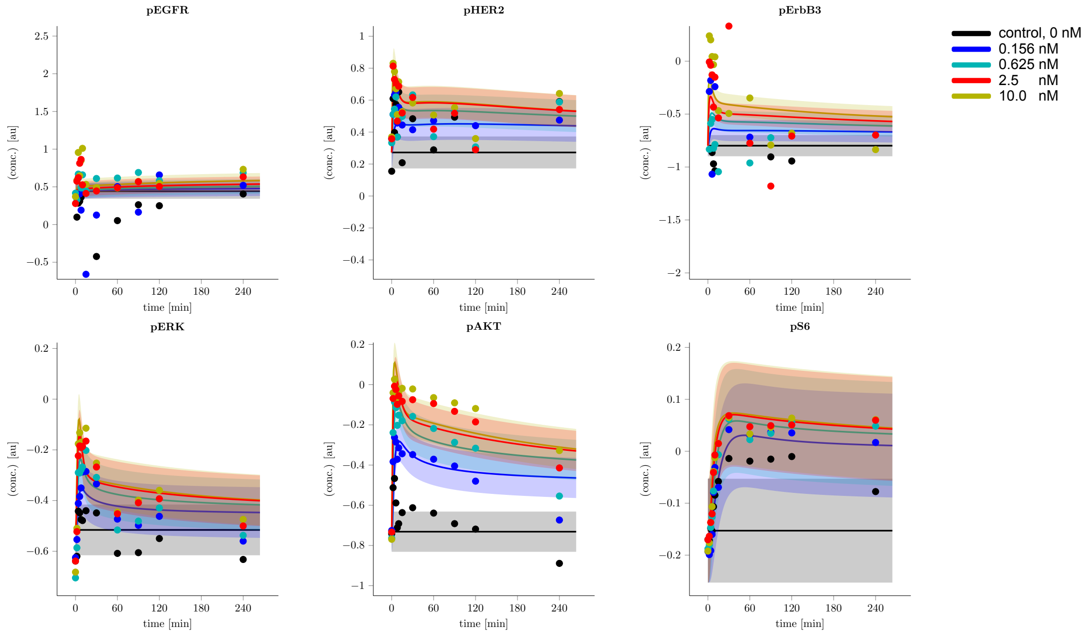

Suppl. Figure 16: Model trajectories for cell line BxPc-3 after HRG stimulation.

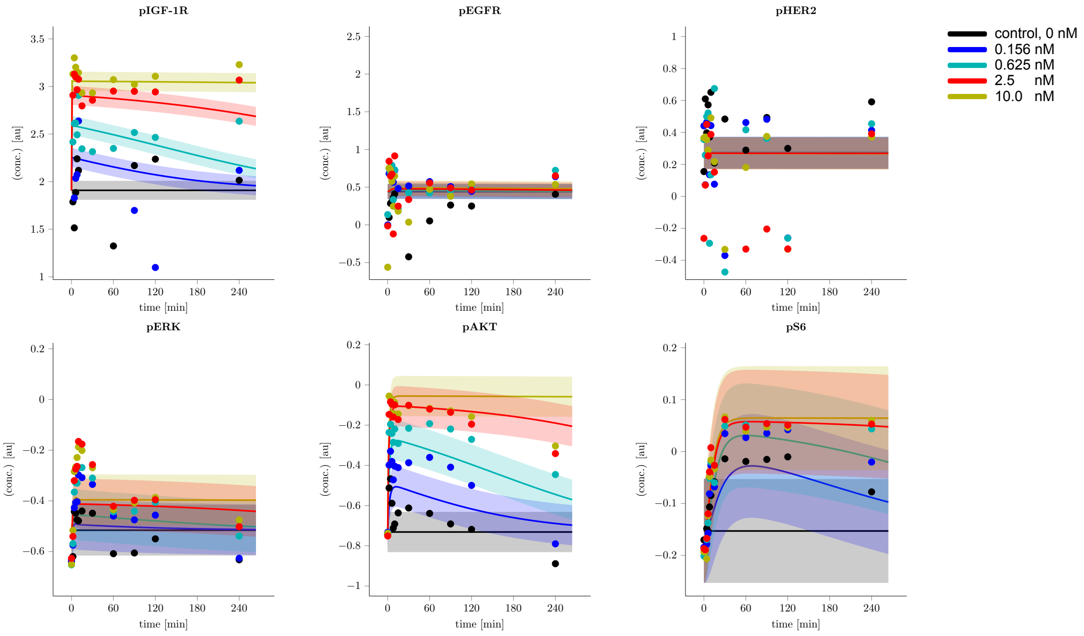

Suppl. Figure 17: Model trajectories for cell line BxPc-3 after IGF-1 stimulation.

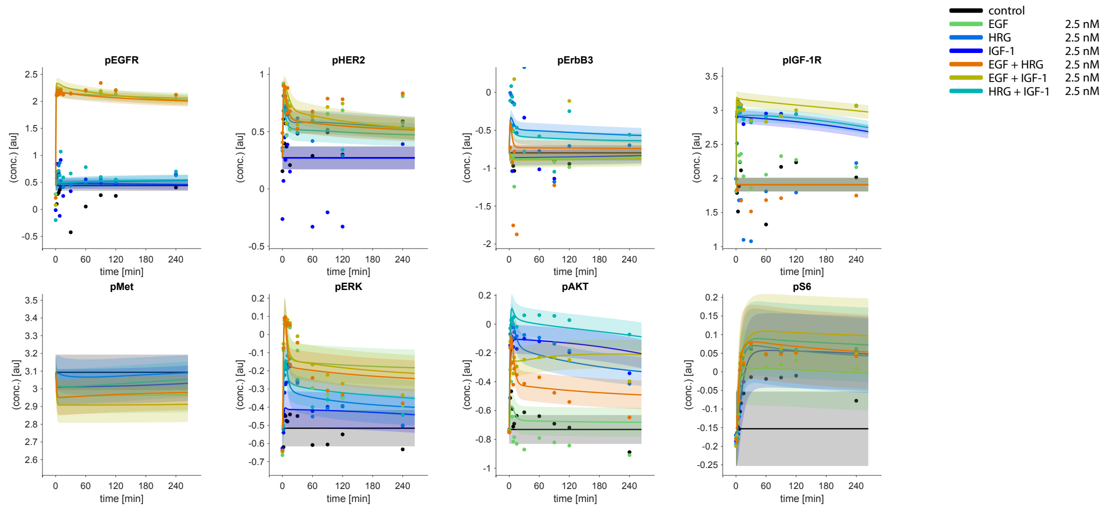

Suppl. Figure 18: Model trajectories for cell line BxPc-3 after ligand co-stimulations.

1.10 Cell line A431

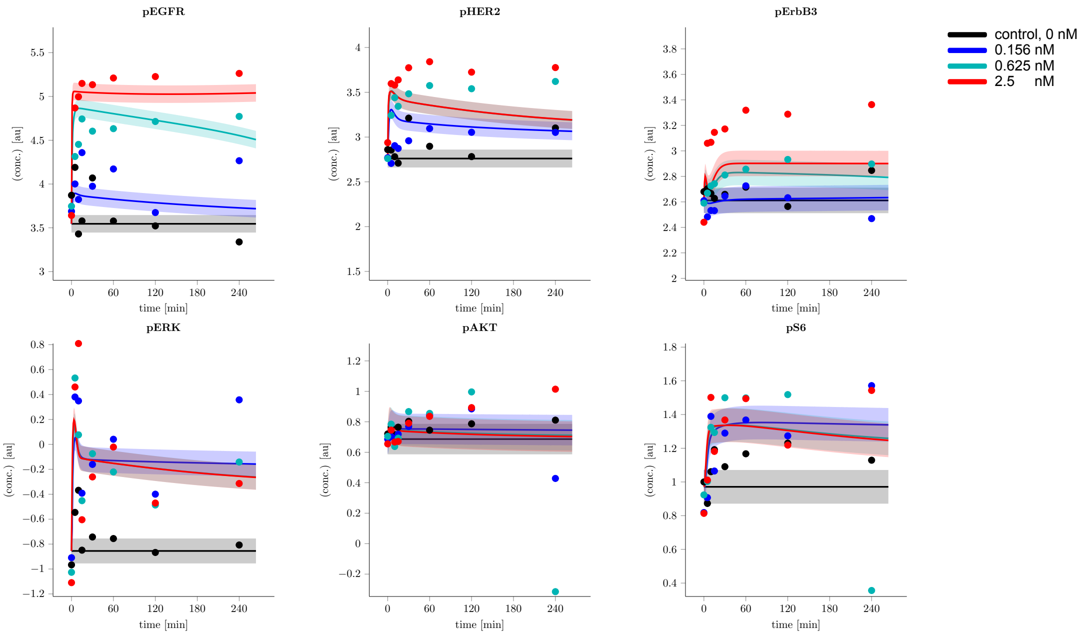

Suppl. Figure 19: Model trajectories for cell line A4431 after EGF stimulation.

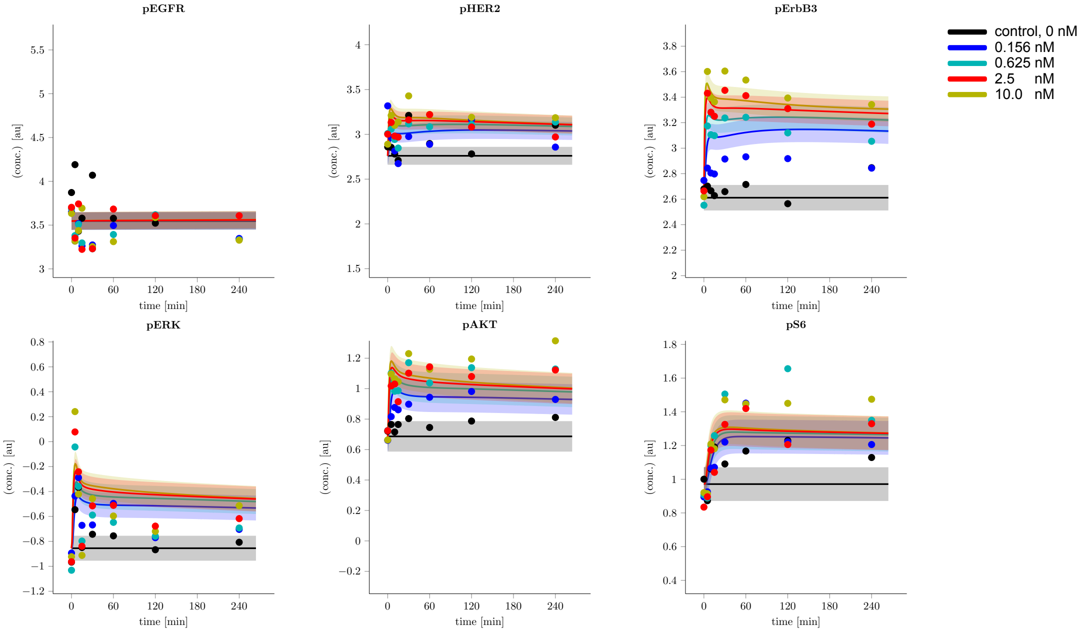

Suppl. Figure 20: Model trajectories for cell line A4431 after HRG stimulation.

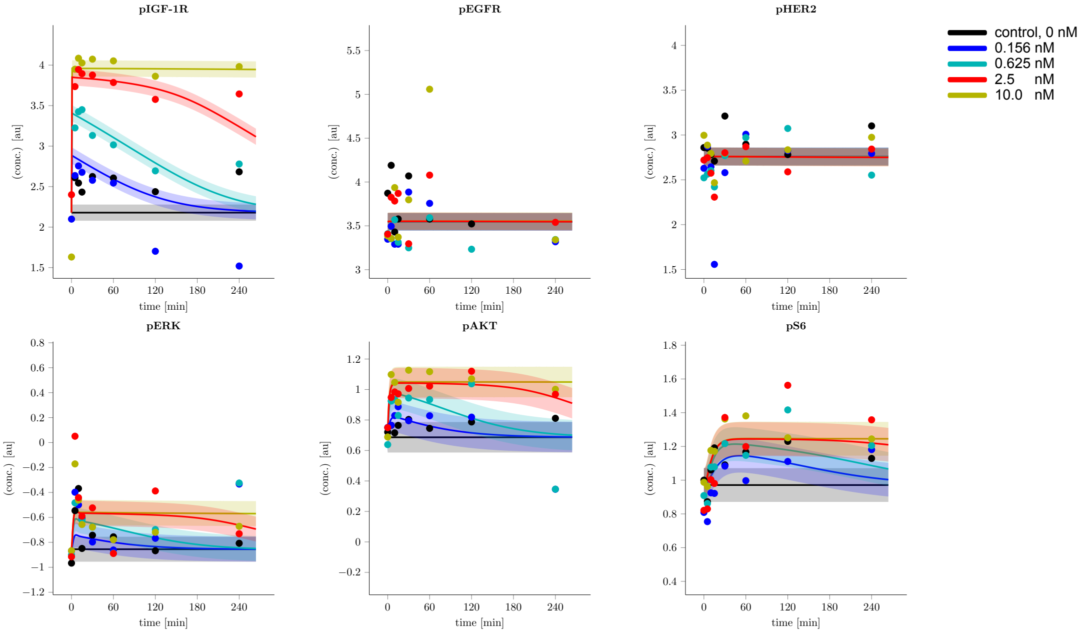

Suppl. Figure 21: Model trajectories for cell line A4431 after IGF-1 stimulation.

1.11 Cell line BT-20

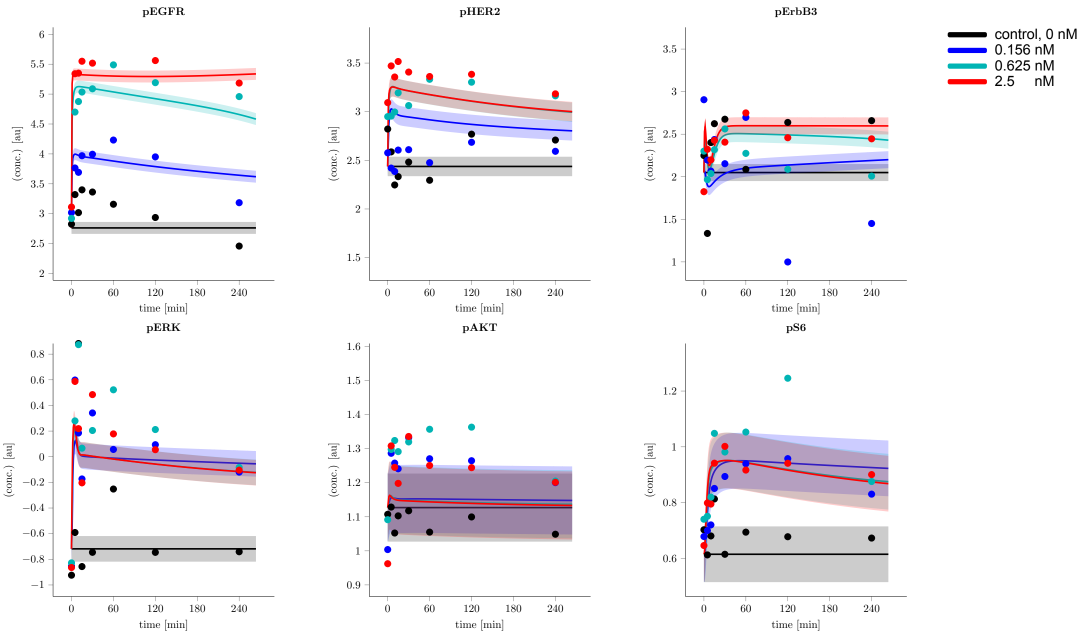

Suppl. Figure 22: Model trajectories for cell line BT-20 after EGF stimulation.

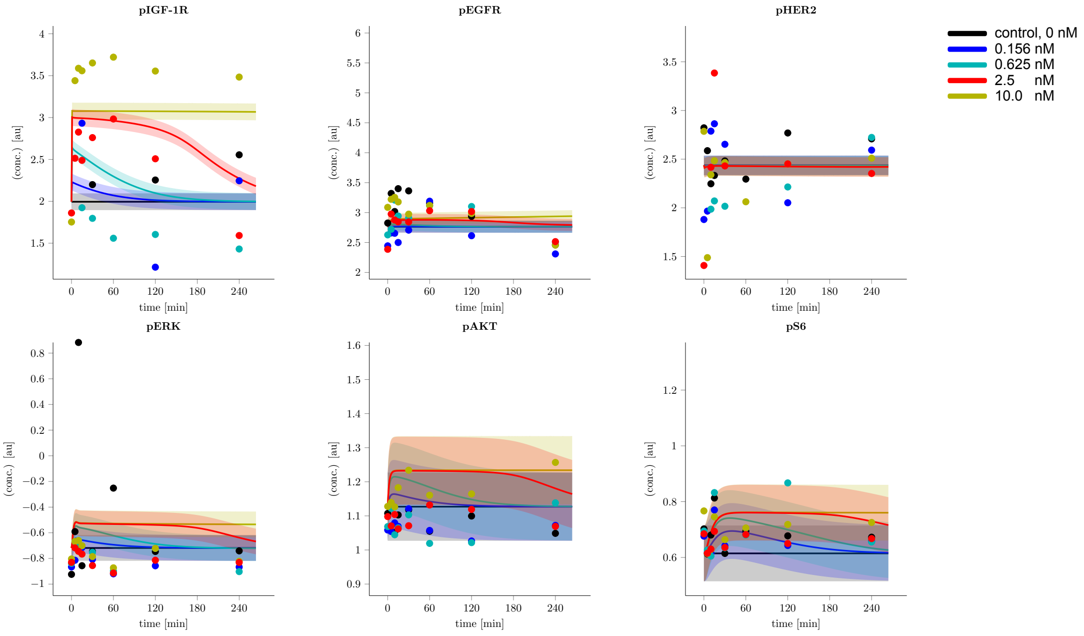

Suppl. Figure 23: Model trajectories for cell line BT-20 after IGF-1 stimulation.

1.12 Cell line IGROV-1

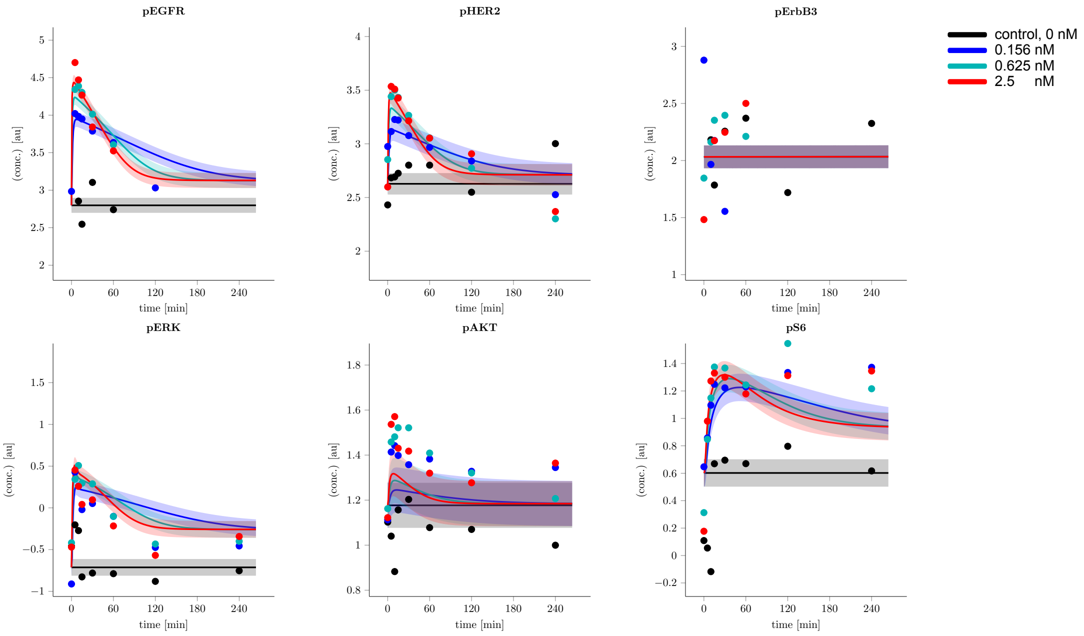

Suppl. Figure 24: Model trajectories for cell line IGROV-1 after EGF stimulation.

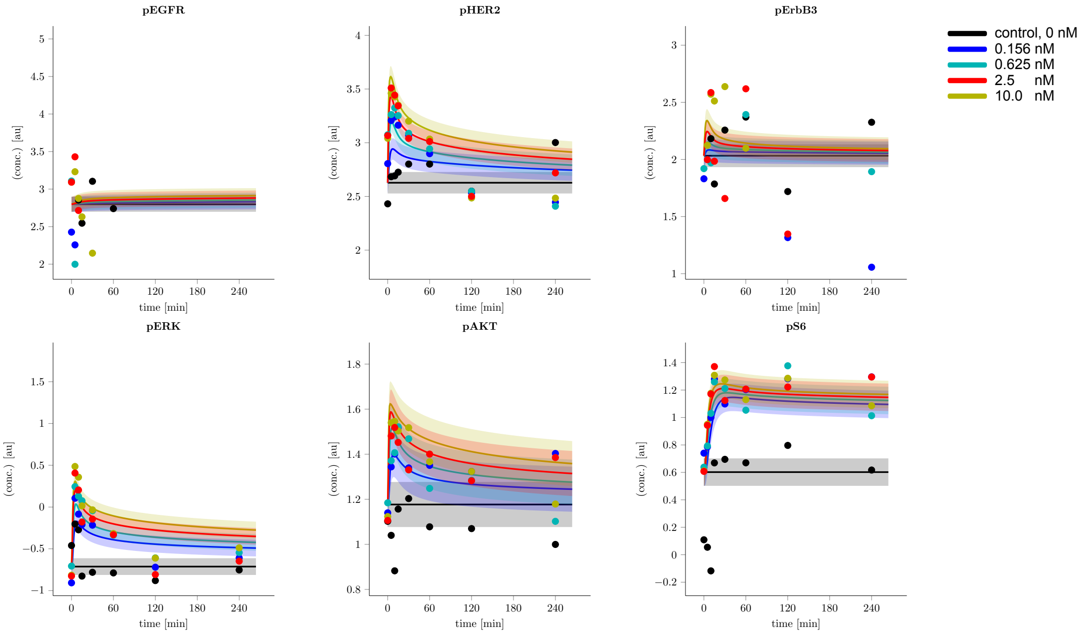

Suppl. Figure 25: Model trajectories for cell line IGROV-1 after HRG stimulation.

1.13 Cell line ADRr

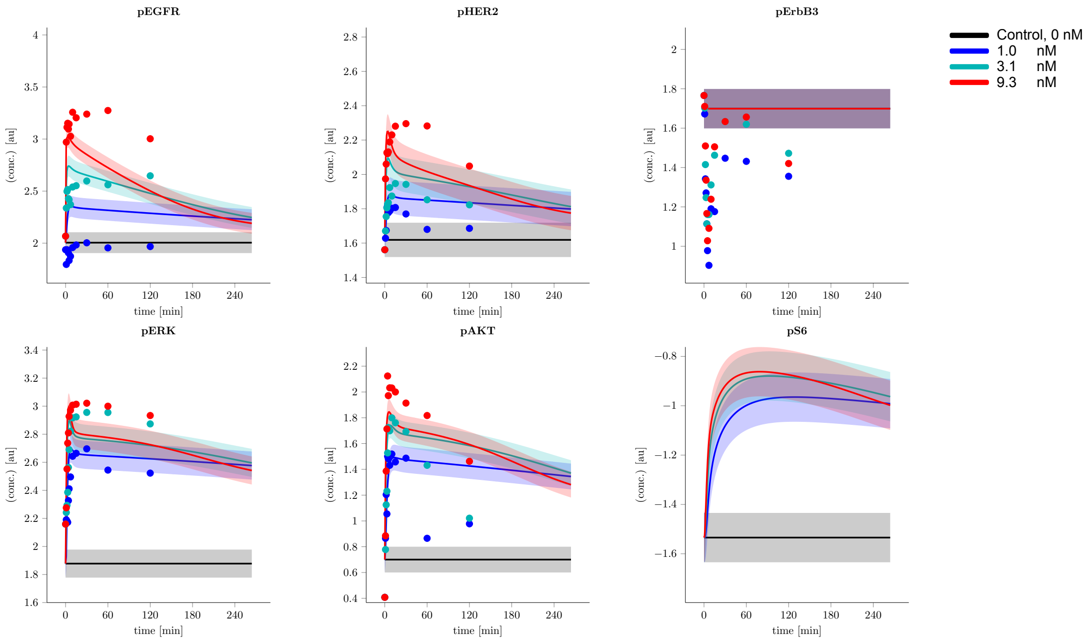

Suppl. Figure 26: Model trajectories for cell line ADRr after EGF stimulation.

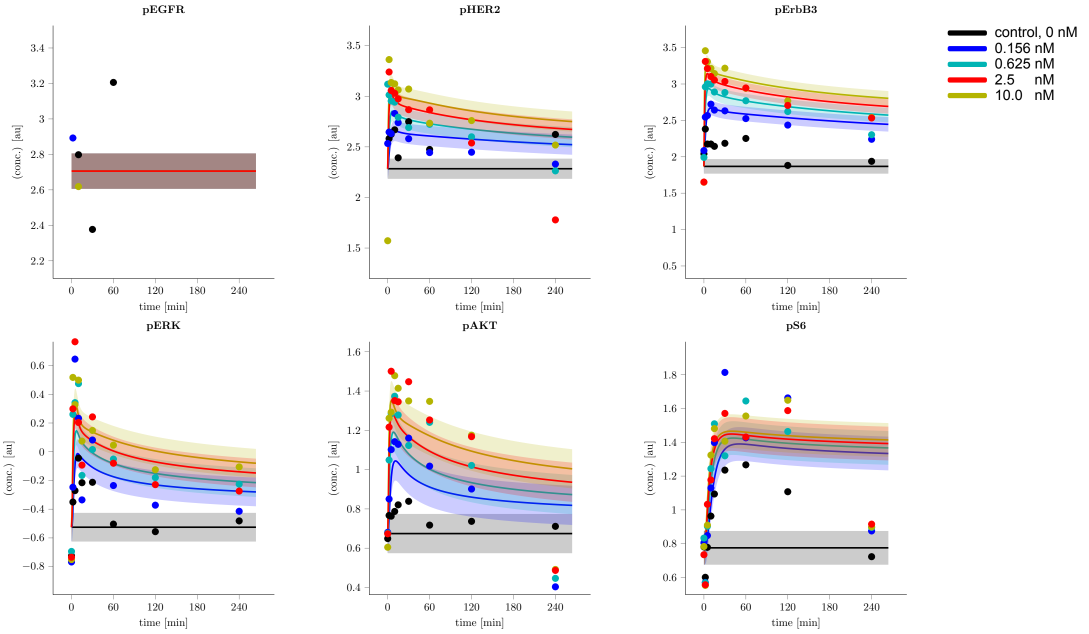

Suppl. Figure 27: Model trajectories for cell line ADRr after HRG stimulation.

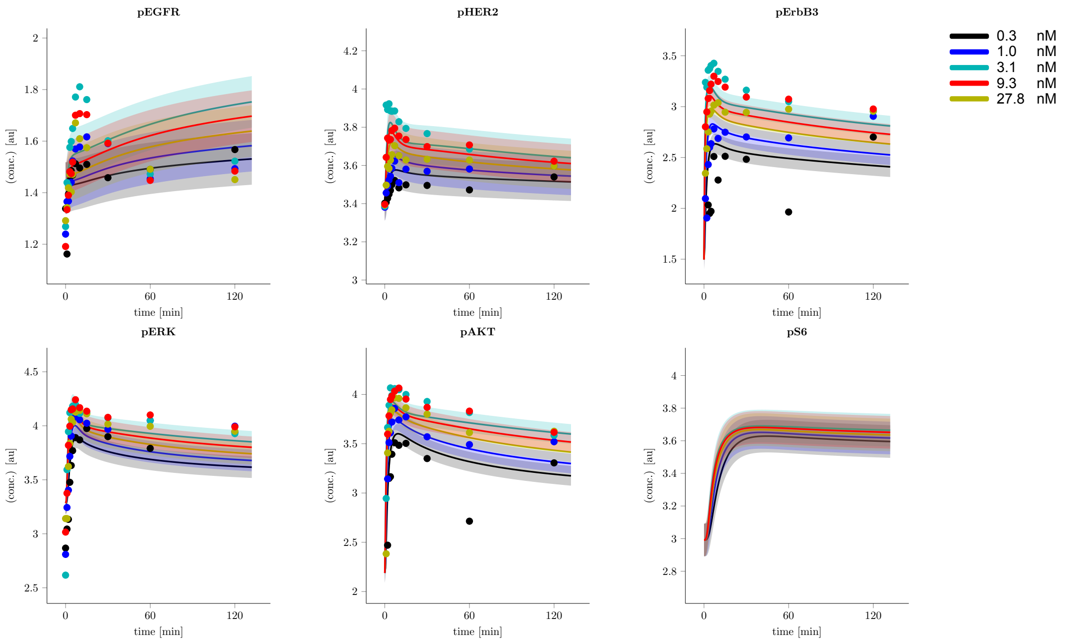

Suppl. Figure 28: Model trajectories for cell line ADRr after HRG stimulation.

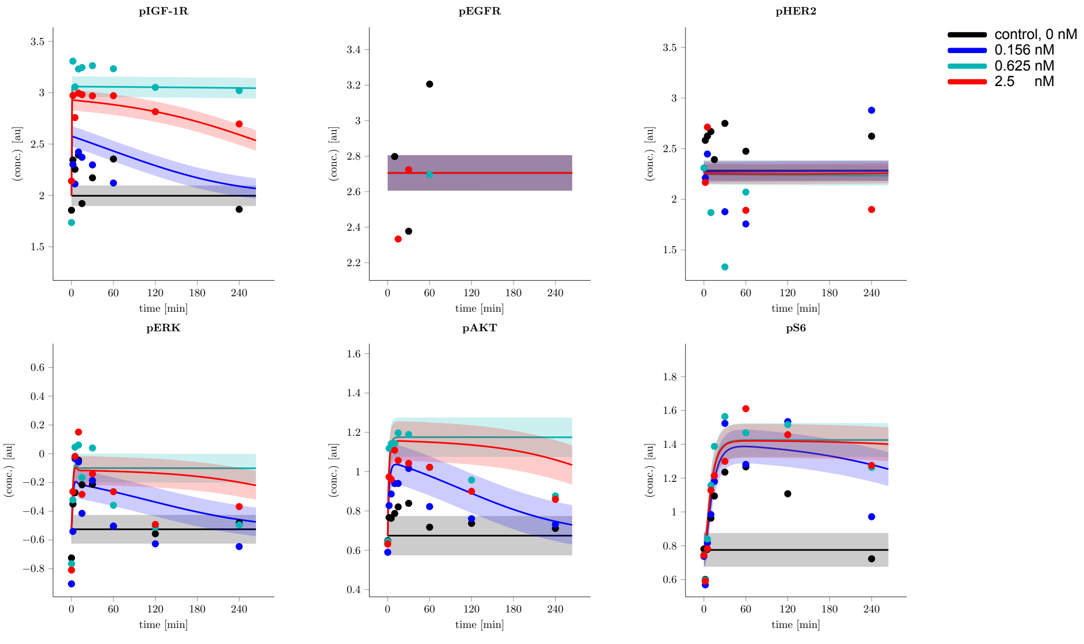

Suppl. Figure 29: Model trajectories for cell line ADRr after IGF-1 stimulation.

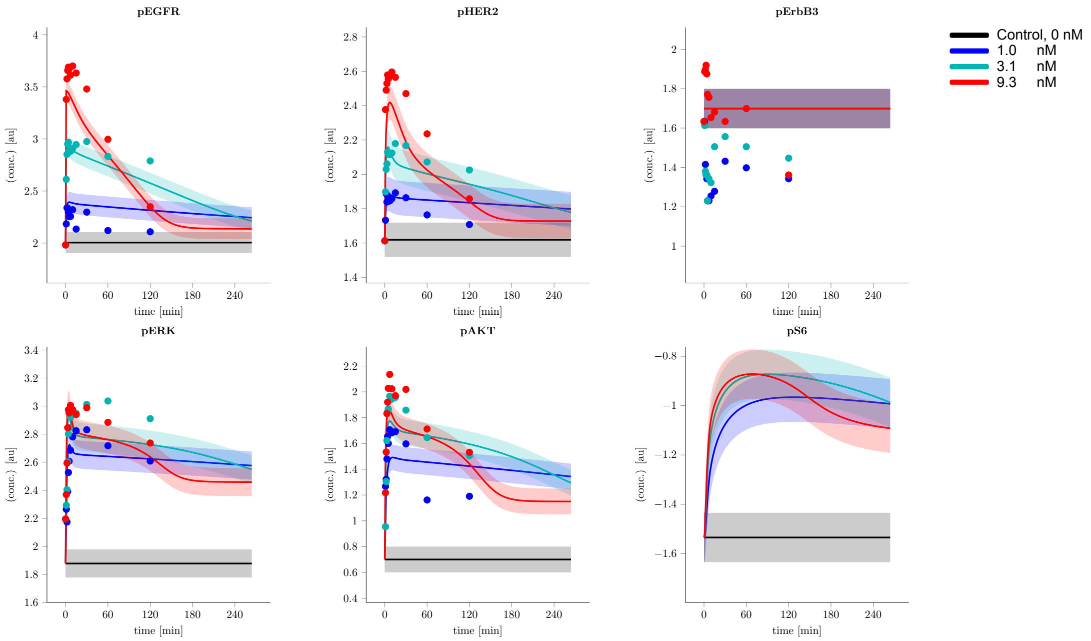

Suppl. Figure 30: Model trajectories for cell line ADRr after BTC stimulation.

1.14 Cell line ACHN

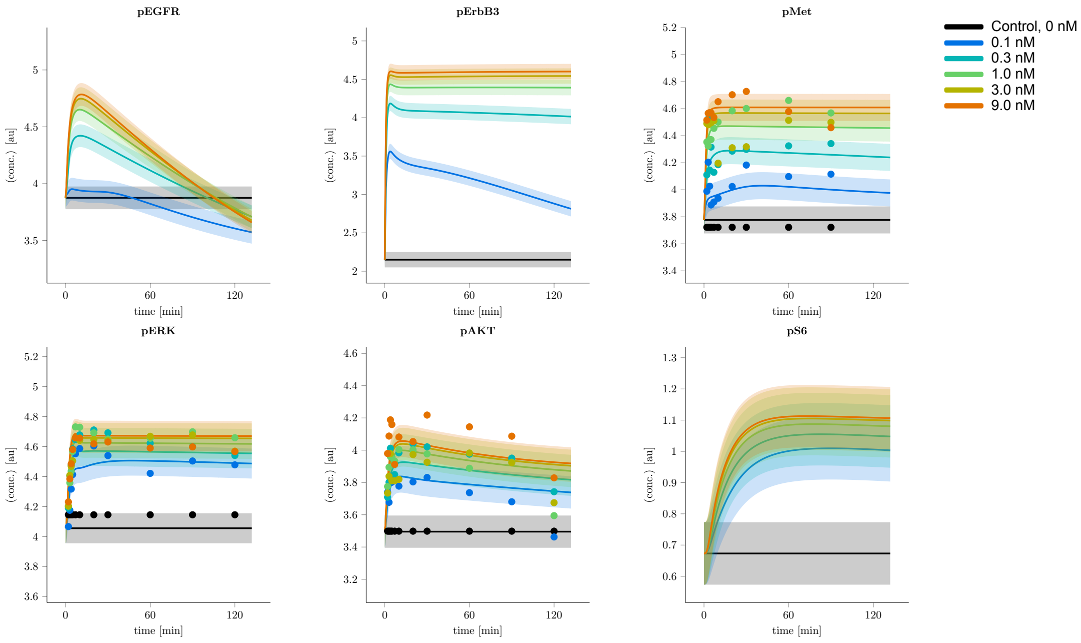

Suppl. Figure 31: Model trajectories for cell line ACHN after HGF stimulation.

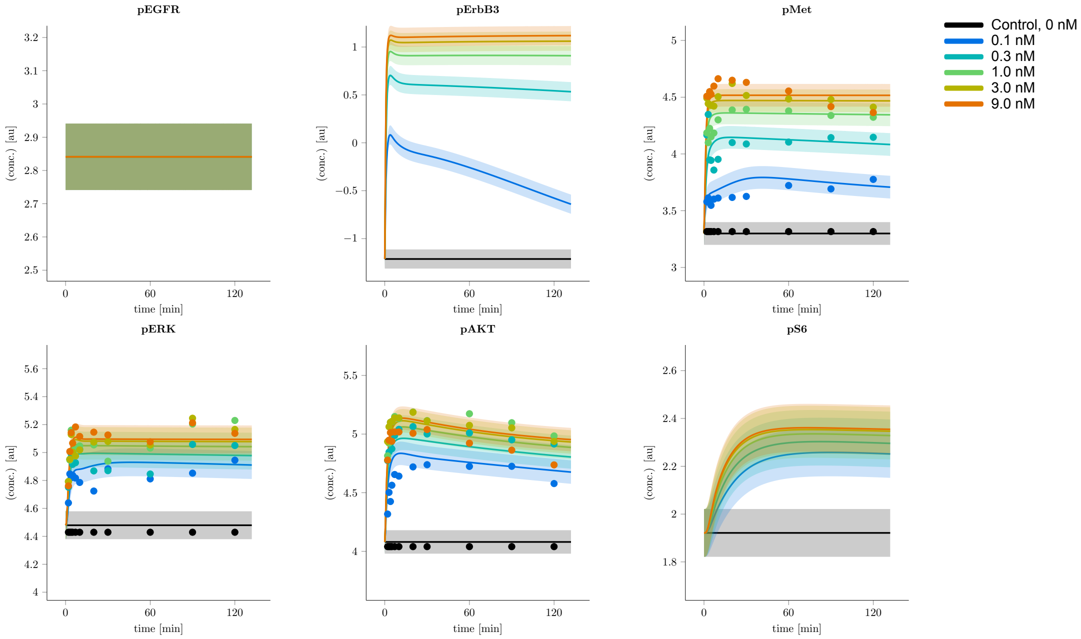

Suppl. Figure 32: Model trajectories for cell line ACHN after HGF stimulation.

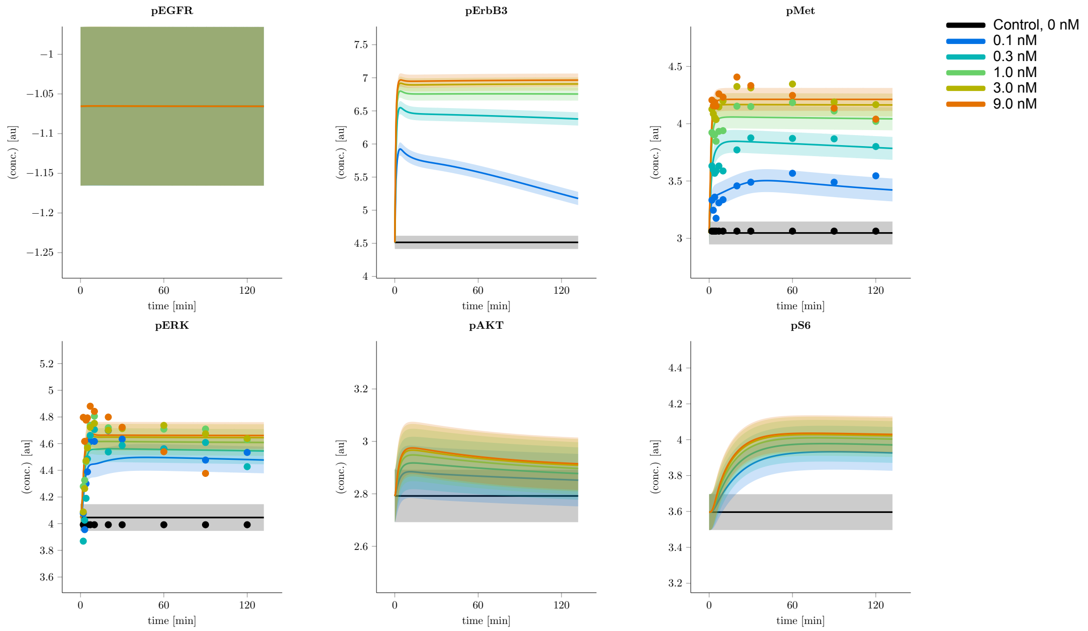

Suppl. Figure 33: Model trajectories for cell line ACHN after HGF stimulation.

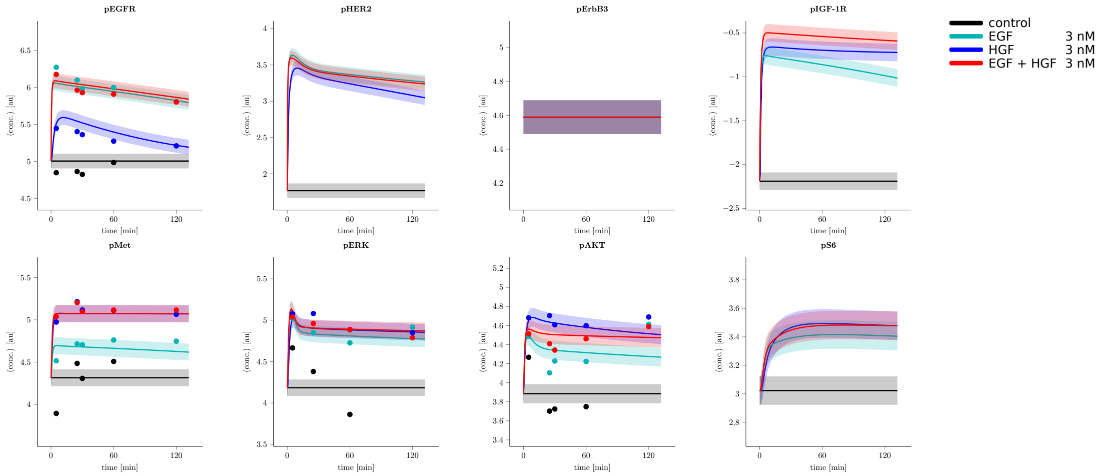

Suppl. Figure 34: Model trajectories for cell line ACHN after HGF+EGF co-stimulation.

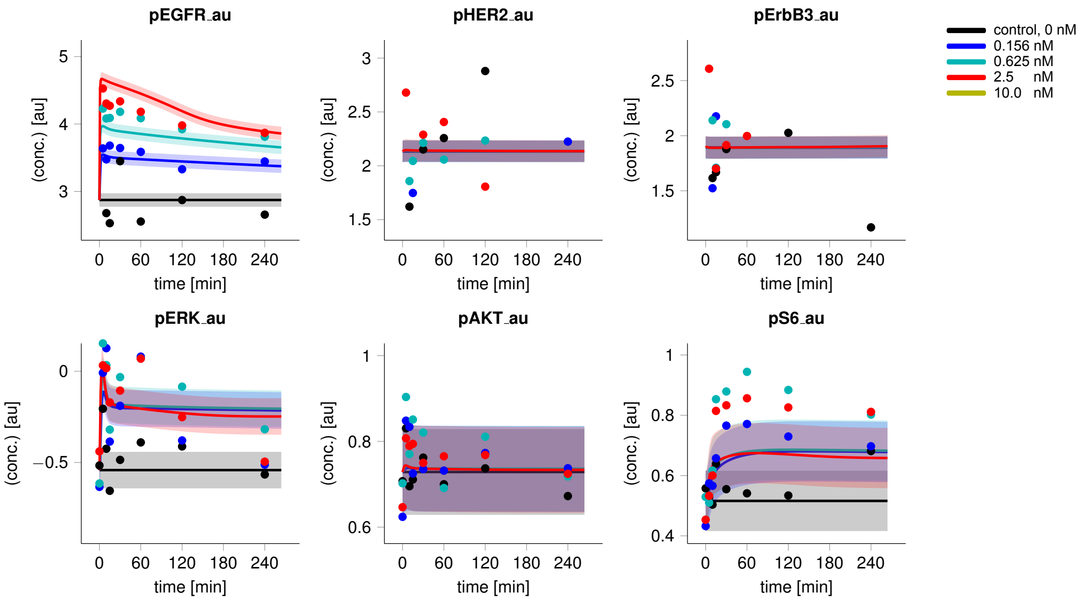

Suppl. Figure 35: Model trajectories for cell line ACHN after EGF stimulation.

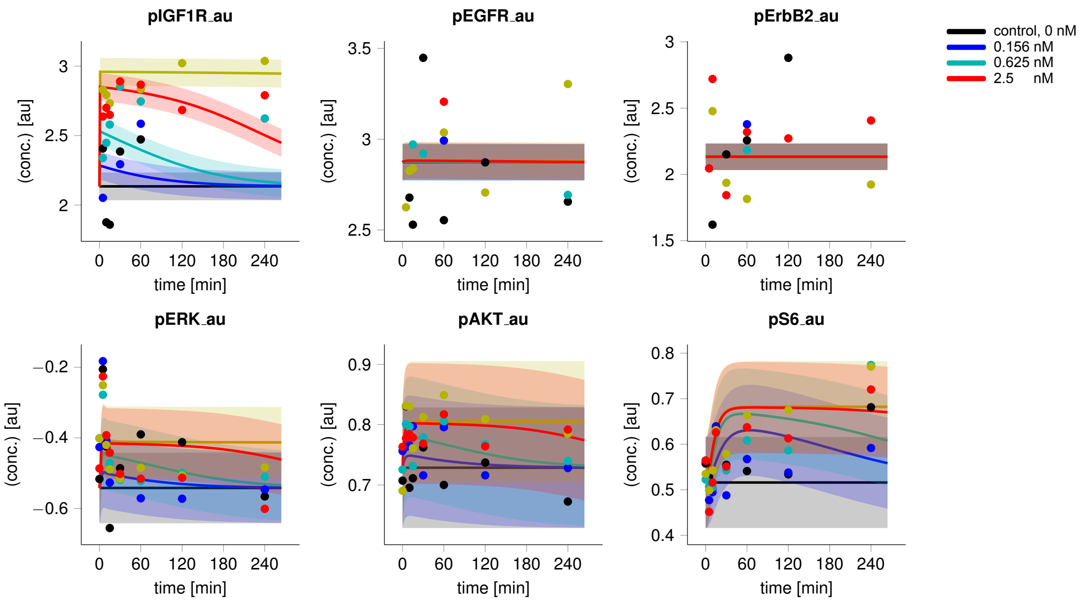

Suppl. Figure 36: Model trajectories for cell line ACHN after IGF-1 stimulation.

1.15 Cell line BT474

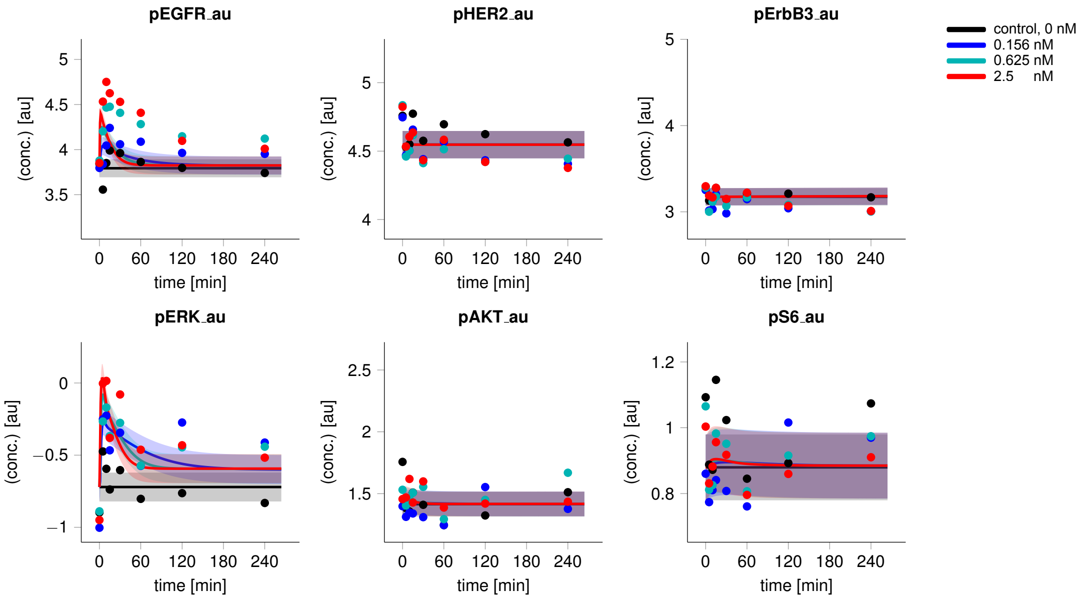

Suppl. Figure 37: Model trajectories for cell line BT474 after EGF stimulation.

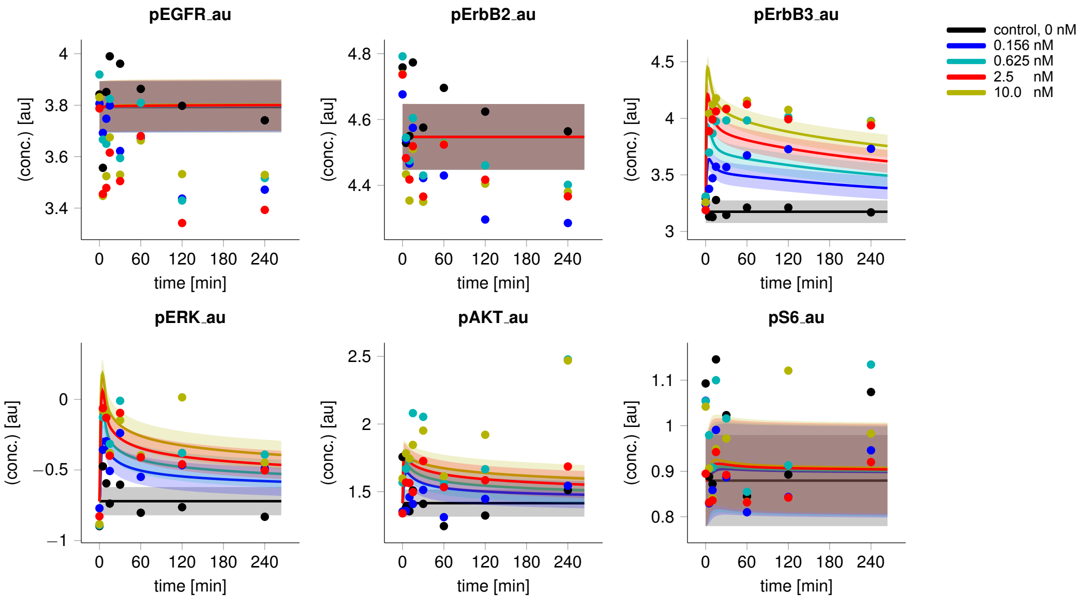

Suppl. Figure 38: Model trajectories for cell line BT474 after HRG stimulation.

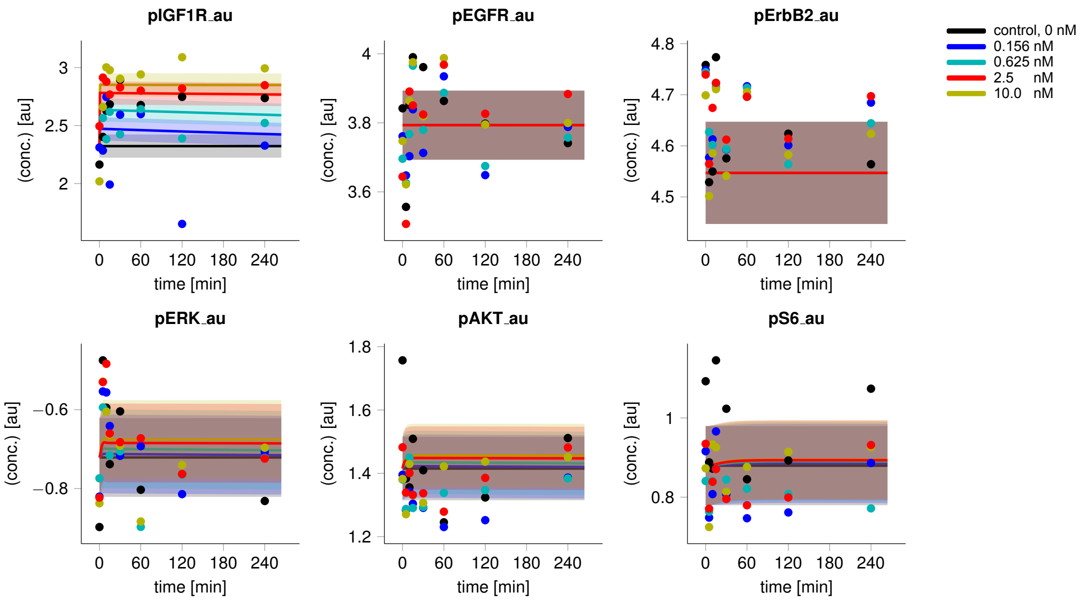

Suppl. Figure 39: Model trajectories for cell line BT474 after IGF-1 stimulation.

1.16 Cell line MDA-MB-231

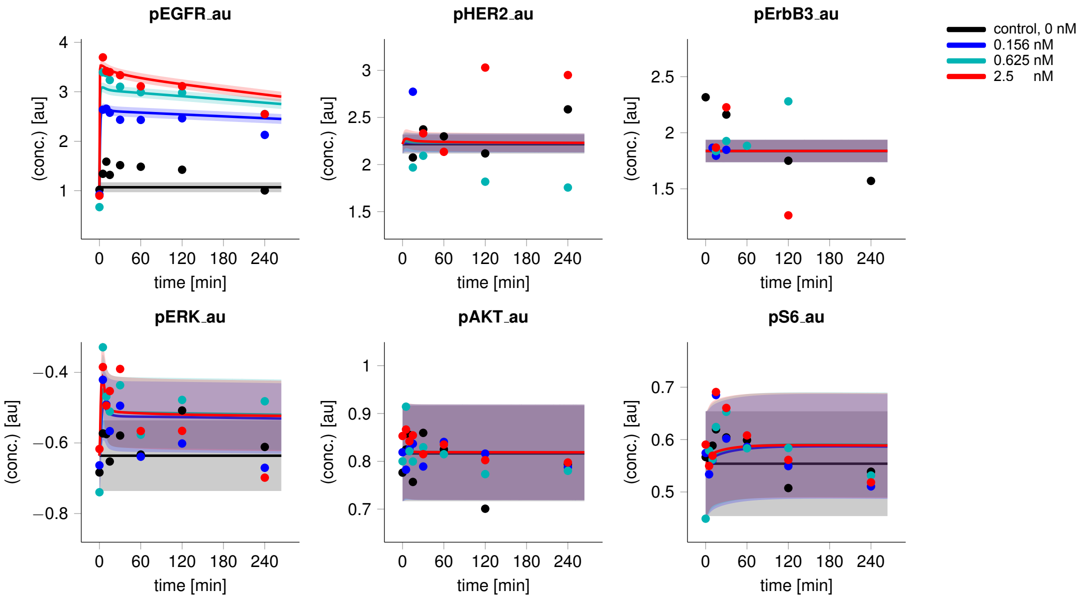

Suppl. Figure 40: Model trajectories for cell line MDA-MB-231 after EGF stimulation.

1.17 Estimated parameters

This section lists all the model parameters used in the model. Parameters highlighted in red color indicate parameter values close to their bounds. The parameter name prefix init_ indicates the initial value of a dynamic variable. The parameter name prefix offset_ indicates a offset of the experimental data. The parameter name prefix scale_ indicates a scaling factor of the experimental data. The parameter name prefix sd_ indicates the magnitude of the measurement noise for a specific measurement.

	name	θ_{min}	$\hat{\theta}$	θ_{max}	log	non-log $\hat{\theta}$	fitted
1	AKT_activation_pEGFR	-5	-5.0000	+3	1	$+1.00 \cdot 10^{-05}$	1
2	AKT_activation_pErbB12	-5	-1.1940	+3	1	$+6.40 \cdot 10^{-02}$	1
3	AKT_activation_pErbB13	-5	+1.1178	+3	1	$+1.31 \cdot 10^{+01}$	1
4	AKT_activation_pErbB32	-5	-0.2513	+3	1	$+5.61 \cdot 10^{-01}$	1
5	AKT_activation_pIGF1R	-5	-0.1641	+3	1	$+6.85 \cdot 10^{-01}$	1
6	AKT_activation_pMetEGFR	-5	-5.0000	+3	1	$+1.00 \cdot 10^{-05}$	1
7	AKT_activation_pMetErbB3	-5	-1.4321	+3	1	$+3.70 \cdot 10^{-02}$	1
8	AKT_activation_pMetd	-5	-0.0479	+3	1	$+8.96 \cdot 10^{-01}$	1
9	AKT_internIGF1R_effect	-5	-4.9907	+3	1	$+1.02 \cdot 10^{-05}$	1
10	EGFR_BTC_binding	-5	-4.6498	+3	1	$+2.24 \cdot 10^{-05}$	1
11	EGFR_BTC_dimerize	-5	+3.0000	+3	1	$+1.00 \cdot 10^{+03}$	1
12	EGFR_ErbB2_BTC_dimerize	-5	+0.2072	+3	1	$+1.61 \cdot 10^{+00}$	1
13	EGFR_ErbB2_basal_act	-5	-5.0000	+6	1	$+1.00 \cdot 10^{-05}$	1
14	EGFR_ErbB2_dimerize	-5	-1.8041	+3	1	$+1.57 \cdot 10^{-02}$	1
15	EGFR_ErbB3_BTC_dimerize	-5	-1.4539	+3	1	$+3.52 \cdot 10^{-02}$	1
16	EGFR_ErbB3_basal_act	-5	-3.1243	+6	1	$+7.51 \cdot 10^{-04}$	1
17	EGFR_ErbB3_dimerize	-5	-2.9275	+3	1	$+1.18 \cdot 10^{-03}$	1
18	EGFR_ErbB3_dimerize_noHRG	-5	-5.0000	+3	1	$+1.00 \cdot 10^{-05}$	1
19	EGFR_basal_activation	-5	-5.0000	+6	1	$+1.00 \cdot 10^{-05}$	1
20	EGFR_basal_recycle	-5	+5.7152	+6	1	$+5.19 \cdot 10^{+05}$	1
21	EGFR_dimerize	-5	-1.2005	+3	1	$+6.30 \cdot 10^{-02}$	1
22	EGFR_lig_binding	-5	-4.7227	+3	1	$+1.89 \cdot 10^{-05}$	1
23	EGF_kD	-5	+0.0000	+3	1	$+1.00 \cdot 10^{+00}$	2
24	ERK_phosphorylation_pMEK	-5	-3.5879	+3	1	$+2.58 \cdot 10^{-04}$	1
25	ErbB2_ErbB3_dimerize	-5	-1.5192	+3	1	$+3.03 \cdot 10^{-02}$	1
26	ErbB2_dimerize	-5	-2.1680	+3	1	$+6.79 \cdot 10^{-03}$	1
27	ErbB2_recycle	-5	-2.1824	+3	1	$+6.57 \cdot 10^{-03}$	1
28	ErbB3_ErbB2_basal_act	-5	-5.0000	+6	1	$+1.00 \cdot 10^{-05}$	1
29	ErbB3_basal_activation	-5	-1.5331	+6	1	$+2.93 \cdot 10^{-02}$	1
30	ErbB3_basal_recycle	-5	-0.1327	+6	1	$+7.37 \cdot 10^{-01}$	1
31	ErbB3_dimerize	-5	-1.3508	+3	1	$+4.46 \cdot 10^{-02}$	1
32	ErbB3_lig_binding	-5	-4.2430	+3	1	$+5.71 \cdot 10^{-05}$	1
33	HGF_kD	-5	-0.5229	+3	1	$+3.00 \cdot 10^{-01}$	2
34	HRG_kD	-5	-1.3010	+3	1	$+5.00 \cdot 10^{-02}$	2
35	IGF1R_basal_activation	-5	-2.9317	+3	1	$+1.17 \cdot 10^{-03}$	1
36	IGF1R_basal_recycle	-5	+3.0000	+3	1	$+1.00 \cdot 10^{+03}$	1
37	IGF1R_dimerize	-5	+1.2337	+3	1	$+1.71 \cdot 10^{+01}$	1
38	IGF1R_lig_binding	-5	-2.8155	+3	1	$+1.53 \cdot 10^{-03}$	1
39	IGF1_kD	-5	-0.5229	+3	1	$+3.00 \cdot 10^{-01}$	2
40	MEK_internIGF1R_effect	-5	-5.0000	+3	1	$+1.00 \cdot 10^{-05}$	1
41	MEK_phosphorylation_pEGFR	-5	-5.0000	+3	1	$+1.00 \cdot 10^{-05}$	1
42	MEK_phosphorylation_pErbB12	-5	-0.5625	+3	1	$+2.74 \cdot 10^{-01}$	1
43	MEK_phosphorylation_pErbB13	-5	-5.0000	+3	1	$+1.00 \cdot 10^{-05}$	1
44	MEK_phosphorylation_pErbB32	-5	-1.2178	+3	1	$+6.06 \cdot 10^{-02}$	1

	name	θ_{min}	$\hat{\theta}$	θ_{max}	log	non-log $\hat{\theta}$	fitted
45	MEK_phosphorylation_pIGF1R	-5	-1.5236	+3	1	$+2.99 \cdot 10^{-02}$	1
46	MEK_phosphorylation_pMetEGFR	-5	-5.0000	+3	1	$+1.00 \cdot 10^{-05}$	1
47	MEK_phosphorylation_pMetErbB3	-5	-1.4164	+3	1	$+3.83 \cdot 10^{-02}$	1
48	MEK_phosphorylation_pMetd	-5	+0.2741	+3	1	$+1.88 \cdot 10^{+00}$	1
49	Met_EGFR_BTC_dimerize	-5	-1.9539	+3	1	$+1.11 \cdot 10^{-02}$	1
50	Met_EGFR_basal_act	-5	-4.7581	+6	1	$+1.75 \cdot 10^{-05}$	1
51	Met_EGFR_dimerize	-5	-3.2898	+3	1	$+5.13 \cdot 10^{-04}$	1
52	Met_ErbB3_basal_act	-5	+0.5177	+6	1	$+3.29 \cdot 10^{+00}$	1
53	Met_ErbB3_dimerize	-5	-1.4308	+3	1	$+3.71 \cdot 10^{-02}$	1
54	Met_basal_act	-5	-5.0000	+6	1	$+1.00 \cdot 10^{-05}$	1
55	Met_dimerize	-5	-2.0378	+3	1	$+9.17 \cdot 10^{-03}$	1
56	Met_lig_ErbB3_dimerize	-5	+2.7536	+3	1	$+5.67 \cdot 10^{+02}$	1
57	Met_lig_binding	-5	-2.3452	+3	1	$+4.52 \cdot 10^{-03}$	1
58	Met_recycle	-5	-0.2656	+3	1	$+5.42 \cdot 10^{-01}$	1
59	S6K1_phosphorylation_pAKT	-5	-0.6024	+3	1	$+2.50 \cdot 10^{-01}$	1
60	S6K1_phosphorylation_pERK	-5	-4.9718	+3	1	$+1.07 \cdot 10^{-05}$	1
61	S6_phosphorylation_pERK	-5	-4.9987	+3	1	$+1.00 \cdot 10^{-05}$	1
62	S6_phosphorylation_pS6K1	-5	-2.0671	+3	1	$+8.57 \cdot 10^{-03}$	1
63	feedback_pAKT	-5	-4.9754	+3	1	$+1.06 \cdot 10^{-05}$	1
64	feedback_pERK	-5	+3.0000	+3	1	$+1.00 \cdot 10^{+03}$	1
65	feedback_pERK_on_AKT	-5	-4.9989	+3	1	$+1.00 \cdot 10^{-05}$	1
66	feedback_pS6K1	-5	-4.6796	+3	1	$+2.09 \cdot 10^{-05}$	1
67	init_AKT	-5	+0.4327	+3	1	$+2.71 \cdot 10^{+00}$	1
68	init_EGFR	-5	+1.2519	+3	1	$+1.79 \cdot 10^{+01}$	1
69	init_EGFR_BTC	-5	+0.0000	+3	0	$+0.00 \cdot 10^{+00}$	2
70	init_EGFR_EGF	-5	+0.0000	+3	0	$+0.00 \cdot 10^{+00}$	2
71	init_ErbB2	-5	+0.7560	+3	1	$+5.70 \cdot 10^{+00}$	1
72	init_ErbB3	-5	+0.3944	+3	1	$+2.48 \cdot 10^{+00}$	1
73	init_ErbB3_HRG	-5	+0.0000	+3	0	$+0.00 \cdot 10^{+00}$	2
74	init_IGF1R	-5	+0.6753	+3	1	$+4.73 \cdot 10^{+00}$	1
75	init_IGF1R_IGF1	-5	+0.0000	+3	0	$+0.00 \cdot 10^{+00}$	2
76	init_MEK	-5	+0.6275	+3	1	$+4.24 \cdot 10^{+00}$	1
77	init_Met	-5	+0.8978	+3	1	$+7.90 \cdot 10^{+00}$	1
78	init_Met_HGF	-5	+0.0000	+3	0	$+0.00 \cdot 10^{+00}$	2
79	init_RTKph	-5	-0.2084	+3	1	$+6.19 \cdot 10^{-01}$	1
80	init_S6	-5	+2.1629	+3	1	$+1.46 \cdot 10^{+02}$	1
81	init_pERK	-5	-0.4759	+3	1	$+3.34 \cdot 10^{-01}$	1
82	init_pS6K1	-5	-2.9054	+3	1	$+1.24 \cdot 10^{-03}$	1
83	offset_pAKT_CelllineA431	-5	+0.6550	+5	1	$+4.52 \cdot 10^{+00}$	1
84	offset_pAKT_CelllineACHN_197	-5	+3.3675	+5	1	$+2.33 \cdot 10^{+03}$	1
85	offset_pAKT_CelllineACHN_200	-5	-2.7863	+5	1	$+1.64 \cdot 10^{-03}$	1
86	offset_pAKT_CelllineACHN_218	-5	+2.7695	+5	1	$+5.88 \cdot 10^{+02}$	1
87	offset_pAKT_CelllineACHN_DM	-5	+3.5929	+5	1	$+3.92 \cdot 10^{+03}$	1
88	offset_pAKT_CelllineADRr	-5	+0.6627	+5	1	$+4.60 \cdot 10^{+00}$	1
89	offset_pAKT_CelllineADRr_B	-5	+2.0000	+5	1	$+1.00 \cdot 10^{+02}$	2

	name	θ_{min}	$\hat{\theta}$	θ_{max}	log	non-log $\hat{\theta}$	fitted
90	offset_pAKT_CelllineADRr_B2	-5	+0.0000	+5	1	$+1.00 \cdot 10^{+00}$	2
91	offset_pAKT_CelllineBT20	-5	+1.1126	+5	1	$+1.30 \cdot 10^{+01}$	1
92	offset_pAKT_CelllineBxPc3	-5	-0.8610	+5	1	$+1.38 \cdot 10^{-01}$	1
93	offset_pAKT_CelllineH322M	-5	-0.7277	+5	1	$+1.87 \cdot 10^{-01}$	1
94	offset_pAKT_CelllineIGROV1	-5	+1.1722	+5	1	$+1.49 \cdot 10^{+01}$	1
95	offset_pEGFR_CelllineA431	-5	+3.5299	+5	1	$+3.39 \cdot 10^{+03}$	1
96	offset_pEGFR_CelllineACHN_197	-5	+2.4035	+5	1	$+2.53 \cdot 10^{+02}$	1
97	offset_pEGFR_CelllineACHN_200	-5	+2.8411	+5	1	$+6.94 \cdot 10^{+02}$	1
98	offset_pEGFR_CelllineACHN_218	-5	-1.0656	+5	1	$+8.60 \cdot 10^{-02}$	1
99	offset_pEGFR_CelllineACHN_DM	-5	+5.0000	+5	1	$+1.00 \cdot 10^{+05}$	1
100	offset_pEGFR_CelllineADRr	-5	+2.7056	+5	1	$+5.08 \cdot 10^{+02}$	1
101	offset_pEGFR_CelllineADRr_B	-5	+1.2553	+5	1	$+1.80 \cdot 10^{+01}$	2
102	offset_pEGFR_CelllineADRr_B2	-5	+2.0000	+5	1	$+1.00 \cdot 10^{+02}$	2
103	offset_pEGFR_CelllineBT20	-5	+2.4567	+5	1	$+2.86 \cdot 10^{+02}$	1
104	offset_pEGFR_CelllineBxPc3	-5	+0.3572	+5	1	$+2.28 \cdot 10^{+00}$	1
105	offset_pEGFR_CelllineH322M	-5	+0.6904	+5	1	$+4.90 \cdot 10^{+00}$	1
106	offset_pEGFR_CelllineIGROV1	-5	+2.7554	+5	1	$+5.69 \cdot 10^{+02}$	1
107	offset_pERK_CelllineA431	-8	-7.0000	-6	1	$+1.00 \cdot 10^{-07}$	2
108	offset_pERK_CelllineACHN_197	-8	-7.0000	-6	1	$+1.00 \cdot 10^{-07}$	2
109	offset_pERK_CelllineACHN_200	-8	-7.0000	-6	1	$+1.00 \cdot 10^{-07}$	2
110	offset_pERK_CelllineACHN_218	-8	-7.0000	-6	1	$+1.00 \cdot 10^{-07}$	2
111	offset_pERK_CelllineACHN_DM	-8	-7.0000	-6	1	$+1.00 \cdot 10^{-07}$	2
112	offset_pERK_CelllineADRr	-8	-7.0000	-6	1	$+1.00 \cdot 10^{-07}$	2
113	offset_pERK_CelllineADRr_B	-8	-7.0000	-6	1	$+1.00 \cdot 10^{-07}$	2
114	offset_pERK_CelllineADRr_B2	-8	-7.0000	-6	1	$+1.00 \cdot 10^{-07}$	2
115	offset_pERK_CelllineBT20	-8	-7.0000	-6	1	$+1.00 \cdot 10^{-07}$	2
116	offset_pERK_CelllineBxPc3	-8	-7.0000	-6	1	$+1.00 \cdot 10^{-07}$	2
117	offset_pERK_CelllineH322M	-8	-7.0000	-6	1	$+1.00 \cdot 10^{-07}$	2
118	offset_pERK_CelllineIGROV1	-8	-7.0000	-6	1	$+1.00 \cdot 10^{-07}$	2
119	offset_pErbB2_CelllineA431	-5	+2.7226	+5	1	$+5.28 \cdot 10^{+02}$	1
120	offset_pErbB2_CelllineACHN_197	-5	-3.7900	+5	1	$+1.62 \cdot 10^{-04}$	1
121	offset_pErbB2_CelllineACHN_200	-5	-4.9351	+5	1	$+1.16 \cdot 10^{-05}$	1
122	offset_pErbB2_CelllineACHN_218	-5	+4.9376	+5	1	$+8.66 \cdot 10^{+04}$	1
123	offset_pErbB2_CelllineACHN_DM	-5	-0.6682	+5	1	$+2.15 \cdot 10^{-01}$	1
124	offset_pErbB2_CelllineADRr	-5	+2.2078	+5	1	$+1.61 \cdot 10^{+02}$	1
125	offset_pErbB2_CelllineADRr_B	-5	+3.3945	+5	1	$+2.48 \cdot 10^{+03}$	2
126	offset_pErbB2_CelllineADRr_B2	-5	+1.5798	+5	1	$+3.80 \cdot 10^{+01}$	2
127	offset_pErbB2_CelllineBT20	-5	+2.3793	+5	1	$+2.39 \cdot 10^{+02}$	1
128	offset_pErbB2_CelllineBxPc3	-5	+0.2581	+5	1	$+1.81 \cdot 10^{+00}$	1
129	offset_pErbB2_CelllineH322M	-5	+0.0385	+5	1	$+1.09 \cdot 10^{+00}$	1
130	offset_pErbB2_CelllineIGROV1	-5	-3.4653	+5	1	$+3.43 \cdot 10^{-04}$	1
131	offset_pErbB3_CelllineA431	-5	+2.5050	+5	1	$+3.20 \cdot 10^{+02}$	1
132	offset_pErbB3_CelllineACHN_197	-5	-1.2364	+5	1	$+5.80 \cdot 10^{-02}$	1
133	offset_pErbB3_CelllineACHN_200	-5	-1.8270	+5	1	$+1.49 \cdot 10^{-02}$	1
134	offset_pErbB3_CelllineACHN_218	-5	+1.6605	+5	1	$+4.58 \cdot 10^{+01}$	1

	name	θ_{min}	$\hat{\theta}$	θ_{max}	log	non-log $\hat{\theta}$	fitted
135	offset_pErbB3_CelllineACHN_DM	-5	+4.5888	+5	1	$+3.88 \cdot 10^{+04}$	1
136	offset_pErbB3_CelllineADRr	-5	+1.4259	+5	1	$+2.67 \cdot 10^{+01}$	1
137	offset_pErbB3_CelllineADRr_B	-5	-3.3965	+5	1	$+4.01 \cdot 10^{-04}$	1
138	offset_pErbB3_CelllineADRr_B2	-5	+1.6990	+5	1	$+5.00 \cdot 10^{+01}$	2
139	offset_pErbB3_CelllineBT20	-5	-3.8148	+5	1	$+1.53 \cdot 10^{-04}$	1
140	offset_pErbB3_CelllineBxPc3	-5	-1.0819	+5	1	$+8.28 \cdot 10^{-02}$	1
141	offset_pErbB3_CelllineH322M	-5	-5.0000	+5	1	$+1.00 \cdot 10^{-05}$	1
142	offset_pErbB3_CelllineIGROV1	-5	+2.0279	+5	1	$+1.07 \cdot 10^{+02}$	1
143	offset_pIGF1R_CelllineA431	-5	+2.1720	+5	1	$+1.49 \cdot 10^{+02}$	1
144	offset_pIGF1R_CelllineACHN_197	-5	-2.0799	+5	1	$+8.32 \cdot 10^{-03}$	1
145	offset_pIGF1R_CelllineACHN_200	-5	-1.2795	+5	1	$+5.25 \cdot 10^{-02}$	1
146	offset_pIGF1R_CelllineACHN_218	-5	+3.7923	+5	1	$+6.20 \cdot 10^{+03}$	1
147	offset_pIGF1R_CelllineACHN_DM	-5	-2.3077	+5	1	$+4.92 \cdot 10^{-03}$	1
148	offset_pIGF1R_CelllineADRr	-5	+1.9956	+5	1	$+9.90 \cdot 10^{+01}$	1
149	offset_pIGF1R_CelllineADRr_B	-5	+3.2540	+5	1	$+1.79 \cdot 10^{+03}$	1
150	offset_pIGF1R_CelllineADRr_B2	-5	+4.8136	+5	1	$+6.51 \cdot 10^{+04}$	1
151	offset_pIGF1R_CelllineBT20	-5	+1.9932	+5	1	$+9.85 \cdot 10^{+01}$	1
152	offset_pIGF1R_CelllineBxPc3	-5	+1.9074	+5	1	$+8.08 \cdot 10^{+01}$	1
153	offset_pIGF1R_CelllineH322M	-5	+2.6096	+5	1	$+4.07 \cdot 10^{+02}$	1
154	offset_pIGF1R_CelllineIGROV1	-5	+2.3416	+5	1	$+2.20 \cdot 10^{+02}$	1
155	offset_pMEK_CelllineA431	-5	+0.7637	+5	1	$+5.80 \cdot 10^{+00}$	1
156	offset_pMEK_CelllineACHN_197	-5	+4.5087	+5	1	$+3.23 \cdot 10^{+04}$	1
157	offset_pMEK_CelllineACHN_200	-5	-1.8933	+5	1	$+1.28 \cdot 10^{-02}$	1
158	offset_pMEK_CelllineACHN_218	-5	+2.7205	+5	1	$+5.25 \cdot 10^{+02}$	1
159	offset_pMEK_CelllineACHN_DM	-5	-0.3988	+5	1	$+3.99 \cdot 10^{-01}$	1
160	offset_pMEK_CelllineADRr	-5	+0.8455	+5	1	$+7.01 \cdot 10^{+00}$	1
161	offset_pMEK_CelllineADRr_B	-5	-0.1498	+5	1	$+7.08 \cdot 10^{-01}$	1
162	offset_pMEK_CelllineADRr_B2	-5	+3.6717	+5	1	$+4.70 \cdot 10^{+03}$	1
163	offset_pMEK_CelllineBT20	-5	+0.8337	+5	1	$+6.82 \cdot 10^{+00}$	1
164	offset_pMEK_CelllineBxPc3	-5	-0.5482	+5	1	$+2.83 \cdot 10^{-01}$	1
165	offset_pMEK_CelllineH322M	-5	-0.8625	+5	1	$+1.37 \cdot 10^{-01}$	1
166	offset_pMEK_CelllineIGROV1	-5	+0.7532	+5	1	$+5.66 \cdot 10^{+00}$	1
167	offset_pMet_CelllineA431	-5	+4.5772	+5	1	$+3.78 \cdot 10^{+04}$	1
168	offset_pMet_CelllineACHN_197	-5	+3.7558	+5	1	$+5.70 \cdot 10^{+03}$	1
169	offset_pMet_CelllineACHN_200	-5	+3.2405	+5	1	$+1.74 \cdot 10^{+03}$	1
170	offset_pMet_CelllineACHN_218	-5	+2.9952	+5	1	$+9.89 \cdot 10^{+02}$	1
171	offset_pMet_CelllineACHN_DM	-5	+4.2970	+5	1	$+1.98 \cdot 10^{+04}$	1
172	offset_pMet_CelllineADRr	-5	-1.6896	+5	1	$+2.04 \cdot 10^{-02}$	1
173	offset_pMet_CelllineADRr_B	-5	+3.8638	+5	1	$+7.31 \cdot 10^{+03}$	1
174	offset_pMet_CelllineADRr_B2	-5	+3.5039	+5	1	$+3.19 \cdot 10^{+03}$	1
175	offset_pMet_CelllineBT20	-5	-3.3032	+5	1	$+4.98 \cdot 10^{-04}$	1
176	offset_pMet_CelllineBxPc3	-5	+0.5379	+5	1	$+3.45 \cdot 10^{+00}$	1
177	offset_pMet_CelllineH322M	-5	-1.5028	+5	1	$+3.14 \cdot 10^{-02}$	1
178	offset_pMet_CelllineIGROV1	-5	+3.4264	+5	1	$+2.67 \cdot 10^{+03}$	1
179	offset_pS6K1_CelllineA431	-8	-7.0000	-6	1	$+1.00 \cdot 10^{-07}$	2

	name	θ_{min}	$\hat{\theta}$	θ_{max}	log	non-log $\hat{\theta}$	fitted
180	offset_pS6K1_CelllineACHN_197	-8	-7.0000	-6	1	$+1.00 \cdot 10^{-07}$	2
181	offset_pS6K1_CelllineACHN_200	-8	-7.0000	-6	1	$+1.00 \cdot 10^{-07}$	2
182	offset_pS6K1_CelllineACHN_218	-8	-7.0000	-6	1	$+1.00 \cdot 10^{-07}$	2
183	offset_pS6K1_CelllineACHN_DM	-8	-7.0000	-6	1	$+1.00 \cdot 10^{-07}$	2
184	offset_pS6K1_CelllineADRr	-8	-7.0000	-6	1	$+1.00 \cdot 10^{-07}$	2
185	offset_pS6K1_CelllineADRr_B	-8	-7.0000	-6	1	$+1.00 \cdot 10^{-07}$	2
186	offset_pS6K1_CelllineADRr_B2	-8	-7.0000	-6	1	$+1.00 \cdot 10^{-07}$	2
187	offset_pS6K1_CelllineBT20	-8	-7.0000	-6	1	$+1.00 \cdot 10^{-07}$	2
188	offset_pS6K1_CelllineBxPc3	-8	-7.0000	-6	1	$+1.00 \cdot 10^{-07}$	2
189	offset_pS6K1_CelllineH322M	-8	-7.0000	-6	1	$+1.00 \cdot 10^{-07}$	2
190	offset_pS6K1_CelllineIGROV1	-8	-7.0000	-6	1	$+1.00 \cdot 10^{-07}$	2
191	offset_pS6_CelllineA431	-8	-7.0000	-6	1	$+1.00 \cdot 10^{-07}$	2
192	offset_pS6_CelllineACHN_197	-8	-7.0000	-6	1	$+1.00 \cdot 10^{-07}$	2
193	offset_pS6_CelllineACHN_200	-8	-7.0000	-6	1	$+1.00 \cdot 10^{-07}$	2
194	offset_pS6_CelllineACHN_218	-8	-7.0000	-6	1	$+1.00 \cdot 10^{-07}$	2
195	offset_pS6_CelllineACHN_DM	-8	-7.0000	-6	1	$+1.00 \cdot 10^{-07}$	2
196	offset_pS6_CelllineADRr	-8	-7.0000	-6	1	$+1.00 \cdot 10^{-07}$	2
197	offset_pS6_CelllineADRr_B	-8	-7.0000	-6	1	$+1.00 \cdot 10^{-07}$	2
198	offset_pS6_CelllineADRr_B2	-8	-7.0000	-6	1	$+1.00 \cdot 10^{-07}$	2
199	offset_pS6_CelllineBT20	-8	-7.0000	-6	1	$+1.00 \cdot 10^{-07}$	2
200	offset_pS6_CelllineBxPc3	-8	-7.0000	-6	1	$+1.00 \cdot 10^{-07}$	2
201	offset_pS6_CelllineH322M	-8	-7.0000	-6	1	$+1.00 \cdot 10^{-07}$	2
202	offset_pS6_CelllineIGROV1	-8	-7.0000	-6	1	$+1.00 \cdot 10^{-07}$	2
203	offset_tEGFR_CelllineA431	-5	-1.3773	+5	1	$+4.19 \cdot 10^{-02}$	1
204	offset_tEGFR_CelllineACHN_197	-5	+2.2315	+5	1	$+1.70 \cdot 10^{+02}$	1
205	offset_tEGFR_CelllineACHN_200	-5	+2.1641	+5	1	$+1.46 \cdot 10^{+02}$	1
206	offset_tEGFR_CelllineACHN_218	-5	-1.4077	+5	1	$+3.91 \cdot 10^{-02}$	1
207	offset_tEGFR_CelllineACHN_DM	-5	+3.7591	+5	1	$+5.74 \cdot 10^{+03}$	1
208	offset_tEGFR_CelllineADRr	-5	+3.1407	+5	1	$+1.38 \cdot 10^{+03}$	1
209	offset_tEGFR_CelllineADRr_B	-5	-0.6157	+5	1	$+2.42 \cdot 10^{-01}$	1
210	offset_tEGFR_CelllineADRr_B2	-5	+1.1308	+5	1	$+1.35 \cdot 10^{+01}$	1
211	offset_tEGFR_CelllineBT20	-5	+3.5677	+5	1	$+3.70 \cdot 10^{+03}$	1
212	offset_tEGFR_CelllineBxPc3	-5	-3.1871	+5	1	$+6.50 \cdot 10^{-04}$	1
213	offset_tEGFR_CelllineH322M	-5	+0.1654	+5	1	$+1.46 \cdot 10^{+00}$	1
214	offset_tEGFR_CelllineIGROV1	-5	+2.0697	+5	1	$+1.17 \cdot 10^{+02}$	1
215	offset_tErbB2_CelllineA431	-5	+2.0018	+5	1	$+1.00 \cdot 10^{+02}$	1
216	offset_tErbB2_CelllineACHN_197	-5	-0.8851	+5	1	$+1.30 \cdot 10^{-01}$	1
217	offset_tErbB2_CelllineACHN_200	-5	+3.5520	+5	1	$+3.56 \cdot 10^{+03}$	1
218	offset_tErbB2_CelllineACHN_218	-5	+4.9383	+5	1	$+8.68 \cdot 10^{+04}$	1
219	offset_tErbB2_CelllineACHN_DM	-5	-2.6110	+5	1	$+2.45 \cdot 10^{-03}$	1
220	offset_tErbB2_CelllineADRr	-5	+2.0931	+5	1	$+1.24 \cdot 10^{+02}$	1
221	offset_tErbB2_CelllineADRr_B	-5	+0.2965	+5	1	$+1.98 \cdot 10^{+00}$	1
222	offset_tErbB2_CelllineADRr_B2	-5	-3.7316	+5	1	$+1.86 \cdot 10^{-04}$	1
223	offset_tErbB2_CelllineBT20	-5	+1.1788	+5	1	$+1.51 \cdot 10^{+01}$	1
224	offset_tErbB2_CelllineBxPc3	-5	+0.4320	+5	1	$+2.70 \cdot 10^{+00}$	1

	name	θ_{min}	$\hat{\theta}$	θ_{max}	log	non-log $\hat{\theta}$	fitted
225	offset_tErbB2_CelllineH322M	-5	+0.8980	+5	1	$+7.91 \cdot 10^{+00}$	1
226	offset_tErbB2_CelllineIGROV1	-5	-1.1435	+5	1	$+7.19 \cdot 10^{-02}$	1
227	offset_tErbB3_CelllineA431	-5	+2.1877	+5	1	$+1.54 \cdot 10^{+02}$	1
228	offset_tErbB3_CelllineACHN_197	-5	-0.6566	+5	1	$+2.20 \cdot 10^{-01}$	1
229	offset_tErbB3_CelllineACHN_200	-5	-0.2856	+5	1	$+5.18 \cdot 10^{-01}$	1
230	offset_tErbB3_CelllineACHN_218	-5	-2.7134	+5	1	$+1.93 \cdot 10^{-03}$	1
231	offset_tErbB3_CelllineACHN_DM	-5	-4.0735	+5	1	$+8.44 \cdot 10^{-05}$	1
232	offset_tErbB3_CelllineADRr	-5	+2.0672	+5	1	$+1.17 \cdot 10^{+02}$	1
233	offset_tErbB3_CelllineADRr_B	-5	+2.5830	+5	1	$+3.83 \cdot 10^{+02}$	1
234	offset_tErbB3_CelllineADRr_B2	-5	+4.0312	+5	1	$+1.07 \cdot 10^{+04}$	1
235	offset_tErbB3_CelllineBT20	-5	+1.8105	+5	1	$+6.46 \cdot 10^{+01}$	1
236	offset_tErbB3_CelllineBxPc3	-5	-0.3117	+5	1	$+4.88 \cdot 10^{-01}$	1
237	offset_tErbB3_CelllineH322M	-5	-0.1047	+5	1	$+7.86 \cdot 10^{-01}$	1
238	offset_tErbB3_CelllineIGROV1	-5	-4.2266	+5	1	$+5.93 \cdot 10^{-05}$	1
239	offset_tIGF1R_CelllineA431	-5	-0.5515	+5	1	$+2.81 \cdot 10^{-01}$	1
240	offset_tIGF1R_CelllineACHN_197	-5	+1.0263	+5	1	$+1.06 \cdot 10^{+01}$	1
241	offset_tIGF1R_CelllineACHN_200	-5	+1.3772	+5	1	$+2.38 \cdot 10^{+01}$	1
242	offset_tIGF1R_CelllineACHN_218	-5	-0.2264	+5	1	$+5.94 \cdot 10^{-01}$	1
243	offset_tIGF1R_CelllineACHN_DM	-5	-2.1151	+5	1	$+7.67 \cdot 10^{-03}$	1
244	offset_tIGF1R_CelllineADRr	-5	-2.6862	+5	1	$+2.06 \cdot 10^{-03}$	1
245	offset_tIGF1R_CelllineADRr_B	-5	+2.1330	+5	1	$+1.36 \cdot 10^{+02}$	1
246	offset_tIGF1R_CelllineADRr_B2	-5	-3.2752	+5	1	$+5.31 \cdot 10^{-04}$	1
247	offset_tIGF1R_CelllineBT20	-5	-0.5438	+5	1	$+2.86 \cdot 10^{-01}$	1
248	offset_tIGF1R_CelllineBxPc3	-5	+0.3426	+5	1	$+2.20 \cdot 10^{+00}$	1
249	offset_tIGF1R_CelllineH322M	-5	-1.3755	+5	1	$+4.21 \cdot 10^{-02}$	1
250	offset_tIGF1R_CelllineIGROV1	-5	-3.5996	+5	1	$+2.51 \cdot 10^{-04}$	1
251	pAKT_deactivation	-5	-0.5186	+3	1	$+3.03 \cdot 10^{-01}$	1
252	pEGFR_degradation	-5	-5.0000	+3	1	$+1.00 \cdot 10^{-05}$	1
253	pEGFR_internalize	-5	+0.7946	+3	1	$+6.23 \cdot 10^{+00}$	1
254	pEGFR_phosphatase_binding	-5	+2.2663	+3	1	$+1.85 \cdot 10^{+02}$	1
255	pEGFRi_dephosph	-5	+1.3369	+3	1	$+2.17 \cdot 10^{+01}$	1
256	pERK_dephosphorylation	-5	-0.2655	+3	1	$+5.43 \cdot 10^{-01}$	1
257	pErbB12_degradation	-5	-0.7403	+3	1	$+1.82 \cdot 10^{-01}$	1
258	pErbB12_internalize	-5	+0.2777	+3	1	$+1.90 \cdot 10^{+00}$	1
259	pErbB12i_dephosph	-5	+3.0000	+3	1	$+1.00 \cdot 10^{+03}$	1
260	pErbB12i_phosphatase	-5	-0.0730	+3	1	$+8.45 \cdot 10^{-01}$	1
261	pErbB13_degradation	-5	+1.6965	+3	1	$+4.97 \cdot 10^{+01}$	1
262	pErbB13_internalize	-5	+3.0000	+3	1	$+1.00 \cdot 10^{+03}$	1
263	pErbB13i_dephosph	-5	+1.7665	+3	1	$+5.84 \cdot 10^{+01}$	1
264	pErbB13i_phosphatase	-5	-4.7972	+3	1	$+1.60 \cdot 10^{-05}$	1
265	pErbB2_degradation	-5	+2.5034	+3	1	$+3.19 \cdot 10^{+02}$	1
266	pErbB2_internalize	-5	+3.0000	+3	1	$+1.00 \cdot 10^{+03}$	1
267	pErbB2i_dephosph	-5	+0.9161	+3	1	$+8.24 \cdot 10^{+00}$	1
268	pErbB2i_phosphatase	-5	+3.0000	+3	1	$+1.00 \cdot 10^{+03}$	1
269	pErbB32_degradation	-5	-0.2414	+3	1	$+5.74 \cdot 10^{-01}$	1

	name	θ_{min}	$\hat{\theta}$	θ_{max}	log	non-log $\hat{\theta}$	fitted
270	pErbB32_internalize	-5	+0.0363	+3	1	$+1.09 \cdot 10^{+00}$	1
271	pErbB32i_dephosph	-5	-1.9237	+3	1	$+1.19 \cdot 10^{-02}$	1
272	pErbB32i_phosphatase	-5	-1.3117	+3	1	$+4.88 \cdot 10^{-02}$	1
273	pErbB3_degradation	-5	-0.0417	+3	1	$+9.08 \cdot 10^{-01}$	1
274	pErbB3_internalize	-5	+3.0000	+3	1	$+1.00 \cdot 10^{+03}$	1
275	pErbB3i_dephosph	-5	+1.2126	+3	1	$+1.63 \cdot 10^{+01}$	1
276	pErbB3i_phosphatase	-5	+1.9148	+3	1	$+8.22 \cdot 10^{+01}$	1
277	pIGF1R_degradation	-5	-5.0000	+3	1	$+1.00 \cdot 10^{-05}$	1
278	pIGF1R_internalize	-5	+3.0000	+3	1	$+1.00 \cdot 10^{+03}$	1
279	pIGF1Ri_dephosph	-5	+2.5207	+3	1	$+3.32 \cdot 10^{+02}$	1
280	pIGF1Ri_phosphatase	-5	+3.0000	+3	1	$+1.00 \cdot 10^{+03}$	1
281	pMEK_dephosphorylation	-5	-0.4839	+3	1	$+3.28 \cdot 10^{-01}$	1
282	pMetEGFR_degradation	-5	+0.1539	+3	1	$+1.43 \cdot 10^{+00}$	1
283	pMetEGFR_internalize	-5	+0.1229	+3	1	$+1.33 \cdot 10^{+00}$	1
284	pMetEGFRi_dephosph	-5	-0.2978	+3	1	$+5.04 \cdot 10^{-01}$	1
285	pMetEGFRi_phosphatase	-5	-4.4470	+3	1	$+3.57 \cdot 10^{-05}$	1
286	pMetErbB3_degradation	-5	+2.9929	+3	1	$+9.84 \cdot 10^{+02}$	1
287	pMetErbB3_internalize	-5	+3.0000	+3	1	$+1.00 \cdot 10^{+03}$	1
288	pMetErbB3i_dephosph	-5	+3.0000	+3	1	$+1.00 \cdot 10^{+03}$	1
289	pMetErbB3i_phosphatase	-5	+3.0000	+3	1	$+1.00 \cdot 10^{+03}$	1
290	pMet_degradation	-5	+0.2802	+3	1	$+1.91 \cdot 10^{+00}$	1
291	pMet_internalize	-5	+3.0000	+3	1	$+1.00 \cdot 10^{+03}$	1
292	pMeti_dephosph	-5	+1.1134	+3	1	$+1.30 \cdot 10^{+01}$	1
293	pMeti_phosphatase	-5	+3.0000	+3	1	$+1.00 \cdot 10^{+03}$	1
294	pS6K1_dephosphorylation	-5	-1.9859	+3	1	$+1.03 \cdot 10^{-02}$	1
295	pS6_dephosphorylation	-5	-0.9252	+3	1	$+1.19 \cdot 10^{-01}$	1
296	relto_A431_init_EGFR	-4	+0.9838	+4	1	$+9.63 \cdot 10^{+00}$	1
297	relto_A431_init_ErbB2	-4	+0.0013	+4	1	$+1.00 \cdot 10^{+00}$	1
298	relto_A431_init_ErbB3	-4	-0.0403	+4	1	$+9.11 \cdot 10^{-01}$	1
299	relto_A431_init_IGF1R	-4	+0.4236	+4	1	$+2.65 \cdot 10^{+00}$	1
300	relto_A431_init_Met	-4	-0.6500	+4	1	$+2.24 \cdot 10^{-01}$	1
301	relto_ACHN_init_EGFR	-4	+0.7324	+4	1	$+5.40 \cdot 10^{+00}$	1
302	relto_ACHN_init_ErbB2	-4	-0.1347	+4	1	$+7.33 \cdot 10^{-01}$	1
303	relto_ACHN_init_ErbB3	-4	-0.3509	+4	1	$+4.46 \cdot 10^{-01}$	1
304	relto_ACHN_init_IGF1R	-4	-0.1530	+4	1	$+7.03 \cdot 10^{-01}$	1
305	relto_ACHN_init_Met	-4	+0.1113	+4	1	$+1.29 \cdot 10^{+00}$	1
306	relto_ADRr_init_EGFR	-4	+0.0119	+4	1	$+1.03 \cdot 10^{+00}$	1
307	relto_ADRr_init_ErbB2	-4	+0.0311	+4	1	$+1.07 \cdot 10^{+00}$	1
308	relto_ADRr_init_ErbB3	-4	-0.2414	+4	1	$+5.74 \cdot 10^{-01}$	1
309	relto_ADRr_init_IGF1R	-4	+0.3059	+4	1	$+2.02 \cdot 10^{+00}$	1
310	relto_ADRr_init_Met	-4	-0.3171	+4	1	$+4.82 \cdot 10^{-01}$	1
311	relto_BT20_init_EGFR	-4	+1.0201	+4	1	$+1.05 \cdot 10^{+01}$	1
312	relto_BT20_init_ErbB2	-4	+0.1754	+4	1	$+1.50 \cdot 10^{+00}$	1
313	relto_BT20_init_ErbB3	-4	+0.2661	+4	1	$+1.85 \cdot 10^{+00}$	1
314	relto_BT20_init_IGF1R	-4	+0.5507	+4	1	$+3.55 \cdot 10^{+00}$	1

	name	θ_{min}	$\hat{\theta}$	θ_{max}	log	non-log $\hat{\theta}$	fitted
315	relto_BT20_init_Met	-4	-0.4177	+4	1	$+3.82 \cdot 10^{-01}$	1
316	relto_IGROV_init_EGFR	-4	-0.0264	+4	1	$+9.41 \cdot 10^{-01}$	1
317	relto_IGROV_init_ErbB2	-4	+0.6286	+4	1	$+4.25 \cdot 10^{+00}$	1
318	relto_IGROV_init_ErbB3	-4	-0.2399	+4	1	$+5.76 \cdot 10^{-01}$	1
319	relto_IGROV_init_IGF1R	-4	-0.7987	+4	1	$+1.59 \cdot 10^{-01}$	1
320	relto_IGROV_init_Met	-4	-0.1926	+4	1	$+6.42 \cdot 10^{-01}$	1
321	relto_init_EGFR	-4	+0.2627	+4	1	$+1.83 \cdot 10^{+00}$	1
322	relto_init_ErbB2	-4	-0.2169	+4	1	$+6.07 \cdot 10^{-01}$	1
323	relto_init_ErbB3	-4	+0.0165	+4	1	$+1.04 \cdot 10^{+00}$	1
324	relto_init_IGF1R	-4	+0.1713	+4	1	$+1.48 \cdot 10^{+00}$	1
325	relto_init_Met	-4	+0.2563	+4	1	$+1.80 \cdot 10^{+00}$	1
326	scale_Ligand	+1	+4.5776	+6	1	$+3.78 \cdot 10^{+04}$	1
327	scale_pAKT_CelllineA431	-4	+0.8937	+6	1	$+7.83 \cdot 10^{+00}$	1
328	scale_pAKT_CelllineACHN_197	-4	+4.5362	+6	1	$+3.44 \cdot 10^{+04}$	1
329	scale_pAKT_CelllineACHN_200	-4	+5.7146	+6	1	$+5.18 \cdot 10^{+05}$	1
330	scale_pAKT_CelllineACHN_218	-4	+3.1301	+6	1	$+1.35 \cdot 10^{+03}$	1
331	scale_pAKT_CelllineACHN_DM	-4	+5.2088	+6	1	$+1.62 \cdot 10^{+05}$	1
332	scale_pAKT_CelllineADRr	-4	+1.1162	+6	1	$+1.31 \cdot 10^{+01}$	1
333	scale_pAKT_CelllineADRr_B	-4	+3.7523	+6	1	$+5.65 \cdot 10^{+03}$	1
334	scale_pAKT_CelllineADRr_B2	-4	+2.6214	+6	1	$+4.18 \cdot 10^{+02}$	1
335	scale_pAKT_CelllineBT20	-4	+0.6415	+6	1	$+4.38 \cdot 10^{+00}$	1
336	scale_pAKT_CelllineBxPc3	-4	-0.0058	+6	1	$+9.87 \cdot 10^{-01}$	1
337	scale_pAKT_CelllineH322M	-4	-0.1464	+6	1	$+7.14 \cdot 10^{-01}$	1
338	scale_pAKT_CelllineIGROV1	-4	+1.0929	+6	1	$+1.24 \cdot 10^{+01}$	1
339	scale_pEGFR_CelllineA431	-4	+2.8971	+6	1	$+7.89 \cdot 10^{+02}$	1
340	scale_pEGFR_CelllineACHN_197	-4	+4.9872	+6	1	$+9.71 \cdot 10^{+04}$	1
341	scale_pEGFR_CelllineACHN_200	-4	-0.5042	+6	1	$+3.13 \cdot 10^{-01}$	1
342	scale_pEGFR_CelllineACHN_218	-4	-3.7650	+6	1	$+1.72 \cdot 10^{-04}$	1
343	scale_pEGFR_CelllineACHN_DM	-4	+4.1539	+6	1	$+1.43 \cdot 10^{+04}$	1
344	scale_pEGFR_CelllineADRr	-4	-0.1229	+6	1	$+7.54 \cdot 10^{-01}$	1
345	scale_pEGFR_CelllineADRr_B	-4	+3.1484	+6	1	$+1.41 \cdot 10^{+03}$	1
346	scale_pEGFR_CelllineADRr_B2	-4	+2.2794	+6	1	$+1.90 \cdot 10^{+02}$	1
347	scale_pEGFR_CelllineBT20	-4	+3.1443	+6	1	$+1.39 \cdot 10^{+03}$	1
348	scale_pEGFR_CelllineBxPc3	-4	+1.3893	+6	1	$+2.45 \cdot 10^{+01}$	1
349	scale_pEGFR_CelllineH322M	-4	+1.5191	+6	1	$+3.30 \cdot 10^{+01}$	1
350	scale_pEGFR_CelllineIGROV1	-4	+3.6998	+6	1	$+5.01 \cdot 10^{+03}$	1
351	scale_pERK_CelllineA431	-4	-0.3792	+6	1	$+4.18 \cdot 10^{-01}$	1
352	scale_pERK_CelllineACHN_197	-4	+4.5316	+6	1	$+3.40 \cdot 10^{+04}$	1
353	scale_pERK_CelllineACHN_200	-4	+4.9551	+6	1	$+9.02 \cdot 10^{+04}$	1
354	scale_pERK_CelllineACHN_218	-4	+4.5220	+6	1	$+3.33 \cdot 10^{+04}$	1
355	scale_pERK_CelllineACHN_DM	-4	+4.6607	+6	1	$+4.58 \cdot 10^{+04}$	1
356	scale_pERK_CelllineADRr	-4	-0.0496	+6	1	$+8.92 \cdot 10^{-01}$	1
357	scale_pERK_CelllineADRr_B	-4	+3.7606	+6	1	$+5.76 \cdot 10^{+03}$	1
358	scale_pERK_CelllineADRr_B2	-4	+2.3537	+6	1	$+2.26 \cdot 10^{+02}$	1
359	scale_pERK_CelllineBT20	-4	-0.2425	+6	1	$+5.72 \cdot 10^{-01}$	1

	name	θ_{min}	$\hat{\theta}$	θ_{max}	log	non-log $\hat{\theta}$	fitted
360	scale_pERK_CelllineBxPc3	-4	-0.0399	+6	1	$+9.12 \cdot 10^{-01}$	1
361	scale_pERK_CelllineH322M	-4	-0.4148	+6	1	$+3.85 \cdot 10^{-01}$	1
362	scale_pERK_CelllineIGROV1	-4	-0.2369	+6	1	$+5.80 \cdot 10^{-01}$	1
363	scale_pErbB2_CelllineA431	-4	+2.9286	+6	1	$+8.48 \cdot 10^{+02}$	1
364	scale_pErbB2_CelllineACHN_197	-4	+5.5897	+6	1	$+3.89 \cdot 10^{+05}$	1
365	scale_pErbB2_CelllineACHN_200	-4	-1.4013	+6	1	$+3.97 \cdot 10^{-02}$	1
366	scale_pErbB2_CelllineACHN_218	-4	+5.4501	+6	1	$+2.82 \cdot 10^{+05}$	1
367	scale_pErbB2_CelllineACHN_DM	-4	+3.3241	+6	1	$+2.11 \cdot 10^{+03}$	1
368	scale_pErbB2_CelllineADRr	-4	+2.8392	+6	1	$+6.91 \cdot 10^{+02}$	1
369	scale_pErbB2_CelllineADRr_B	-4	+3.3188	+6	1	$+2.08 \cdot 10^{+03}$	1
370	scale_pErbB2_CelllineADRr_B2	-4	+1.9030	+6	1	$+8.00 \cdot 10^{+01}$	1
371	scale_pErbB2_CelllineBT20	-4	+2.4723	+6	1	$+2.97 \cdot 10^{+02}$	1
372	scale_pErbB2_CelllineBxPc3	-4	+0.5790	+6	1	$+3.79 \cdot 10^{+00}$	1
373	scale_pErbB2_CelllineH322M	-4	+0.7220	+6	1	$+5.27 \cdot 10^{+00}$	1
374	scale_pErbB2_CelllineIGROV1	-4	+2.8038	+6	1	$+6.37 \cdot 10^{+02}$	1
375	scale_pErbB3_CelllineA431	-4	+3.1879	+6	1	$+1.54 \cdot 10^{+03}$	1
376	scale_pErbB3_CelllineACHN_197	-4	+3.2330	+6	1	$+1.71 \cdot 10^{+03}$	1
377	scale_pErbB3_CelllineACHN_200	-4	-0.2498	+6	1	$+5.63 \cdot 10^{-01}$	1
378	scale_pErbB3_CelllineACHN_218	-4	+5.5970	+6	1	$+3.95 \cdot 10^{+05}$	1
379	scale_pErbB3_CelllineACHN_DM	-4	-2.7580	+6	1	$+1.75 \cdot 10^{-03}$	1
380	scale_pErbB3_CelllineADRr	-4	+3.0066	+6	1	$+1.02 \cdot 10^{+03}$	1
381	scale_pErbB3_CelllineADRr_B	-4	+2.8293	+6	1	$+6.75 \cdot 10^{+02}$	1
382	scale_pErbB3_CelllineADRr_B2	-4	-3.8820	+6	1	$+1.31 \cdot 10^{-04}$	1
383	scale_pErbB3_CelllineBT20	-4	+2.7342	+6	1	$+5.42 \cdot 10^{+02}$	1
384	scale_pErbB3_CelllineBxPc3	-4	-0.5592	+6	1	$+2.76 \cdot 10^{-01}$	1
385	scale_pErbB3_CelllineH322M	-4	+0.4332	+6	1	$+2.71 \cdot 10^{+00}$	1
386	scale_pErbB3_CelllineIGROV1	-4	+1.2609	+6	1	$+1.82 \cdot 10^{+01}$	1
387	scale_pIGF1R_CelllineA431	-5	+3.0416	+6	1	$+1.10 \cdot 10^{+03}$	1
388	scale_pIGF1R_CelllineACHN_197	-4	+2.8211	+6	1	$+6.62 \cdot 10^{+02}$	1
389	scale_pIGF1R_CelllineACHN_200	-4	+2.6575	+6	1	$+4.54 \cdot 10^{+02}$	1
390	scale_pIGF1R_CelllineACHN_218	-4	-3.6359	+6	1	$+2.31 \cdot 10^{-04}$	1
391	scale_pIGF1R_CelllineACHN_DM	-4	+1.0222	+6	1	$+1.05 \cdot 10^{+01}$	1
392	scale_pIGF1R_CelllineADRr	-4	+2.2941	+6	1	$+1.97 \cdot 10^{+02}$	1
393	scale_pIGF1R_CelllineADRr_B	-4	-1.8424	+6	1	$+1.44 \cdot 10^{-02}$	1
394	scale_pIGF1R_CelllineADRr_B2	-4	+5.3359	+6	1	$+2.17 \cdot 10^{+05}$	1
395	scale_pIGF1R_CelllineBT20	-5	+1.9583	+6	1	$+9.08 \cdot 10^{+01}$	1
396	scale_pIGF1R_CelllineBxPc3	-5	+2.5348	+6	1	$+3.43 \cdot 10^{+02}$	1
397	scale_pIGF1R_CelllineH322M	-5	+4.0764	+6	1	$+1.19 \cdot 10^{+04}$	1
398	scale_pIGF1R_CelllineIGROV1	-4	+3.3702	+6	1	$+2.35 \cdot 10^{+03}$	1
399	scale_pMEK_CelllineA431	-4	+3.3420	+6	1	$+2.20 \cdot 10^{+03}$	1
400	scale_pMEK_CelllineACHN_197	-4	-3.6696	+6	1	$+2.14 \cdot 10^{-04}$	1
401	scale_pMEK_CelllineACHN_200	-4	-3.1682	+6	1	$+6.79 \cdot 10^{-04}$	1
402	scale_pMEK_CelllineACHN_218	-4	+1.3145	+6	1	$+2.06 \cdot 10^{+01}$	1
403	scale_pMEK_CelllineACHN_DM	-4	+0.8402	+6	1	$+6.92 \cdot 10^{+00}$	1
404	scale_pMEK_CelllineADRr	-4	-3.1701	+6	1	$+6.76 \cdot 10^{-04}$	1

	name	θ_{min}	$\hat{\theta}$	θ_{max}	log	non-log $\hat{\theta}$	fitted
405	scale_pMEK_CelllineADRr_B	-4	+2.6717	+6	1	$+4.70 \cdot 10^{+02}$	1
406	scale_pMEK_CelllineADRr_B2	-4	+3.4004	+6	1	$+2.51 \cdot 10^{+03}$	1
407	scale_pMEK_CelllineBT20	-4	+3.5633	+6	1	$+3.66 \cdot 10^{+03}$	1
408	scale_pMEK_CelllineBxPc3	-4	+3.1279	+6	1	$+1.34 \cdot 10^{+03}$	1
409	scale_pMEK_CelllineH322M	-4	+2.5448	+6	1	$+3.51 \cdot 10^{+02}$	1
410	scale_pMEK_CelllineIGROV1	-4	+3.8842	+6	1	$+7.66 \cdot 10^{+03}$	1
411	scale_pMet_CelllineA431	-4	-2.1929	+6	1	$+6.41 \cdot 10^{-03}$	1
412	scale_pMet_CelllineACHN_197	-4	+3.4525	+6	1	$+2.83 \cdot 10^{+03}$	1
413	scale_pMet_CelllineACHN_200	-4	+3.4015	+6	1	$+2.52 \cdot 10^{+03}$	1
414	scale_pMet_CelllineACHN_218	-4	+3.0937	+6	1	$+1.24 \cdot 10^{+03}$	1
415	scale_pMet_CelllineACHN_DM	-4	+3.9502	+6	1	$+8.92 \cdot 10^{+03}$	1
416	scale_pMet_CelllineADRr	-4	+0.9621	+6	1	$+9.16 \cdot 10^{+00}$	1
417	scale_pMet_CelllineADRr_B	-4	-1.8174	+6	1	$+1.52 \cdot 10^{-02}$	1
418	scale_pMet_CelllineADRr_B2	-4	-1.1938	+6	1	$+6.40 \cdot 10^{-02}$	1
419	scale_pMet_CelllineBT20	-4	-3.0302	+6	1	$+9.33 \cdot 10^{-04}$	1
420	scale_pMet_CelllineBxPc3	-4	+3.6848	+6	1	$+4.84 \cdot 10^{+03}$	1
421	scale_pMet_CelllineH322M	-4	-2.1851	+6	1	$+6.53 \cdot 10^{-03}$	1
422	scale_pMet_CelllineIGROV1	-4	-3.4428	+6	1	$+3.61 \cdot 10^{-04}$	1
423	scale_pS6K1_CelllineA431	-4	-0.2278	+6	1	$+5.92 \cdot 10^{-01}$	1
424	scale_pS6K1_CelllineACHN_197	-4	+5.6430	+6	1	$+4.40 \cdot 10^{+05}$	1
425	scale_pS6K1_CelllineACHN_200	-4	-3.4246	+6	1	$+3.76 \cdot 10^{-04}$	1
426	scale_pS6K1_CelllineACHN_218	-4	+4.2325	+6	1	$+1.71 \cdot 10^{+04}$	1
427	scale_pS6K1_CelllineACHN_DM	-4	-0.7888	+6	1	$+1.63 \cdot 10^{-01}$	1
428	scale_pS6K1_CelllineADRr	-4	+1.1418	+6	1	$+1.39 \cdot 10^{+01}$	1
429	scale_pS6K1_CelllineADRr_B	-4	+5.6021	+6	1	$+4.00 \cdot 10^{+05}$	1
430	scale_pS6K1_CelllineADRr_B2	-4	+0.0570	+6	1	$+1.14 \cdot 10^{+00}$	1
431	scale_pS6K1_CelllineBT20	-4	+4.8429	+6	1	$+6.97 \cdot 10^{+04}$	1
432	scale_pS6K1_CelllineBxPc3	-4	+2.6714	+6	1	$+4.69 \cdot 10^{+02}$	1
433	scale_pS6K1_CelllineH322M	-4	+2.5079	+6	1	$+3.22 \cdot 10^{+02}$	1
434	scale_pS6K1_CelllineIGROV1	-4	+3.7881	+6	1	$+6.14 \cdot 10^{+03}$	1
435	scale_pS6_CelllineA431	-4	+2.7367	+6	1	$+5.45 \cdot 10^{+02}$	1
436	scale_pS6_CelllineACHN_197	-4	+2.4387	+6	1	$+2.75 \cdot 10^{+02}$	1
437	scale_pS6_CelllineACHN_200	-4	+3.6869	+6	1	$+4.86 \cdot 10^{+03}$	1
438	scale_pS6_CelllineACHN_218	-4	+5.3620	+6	1	$+2.30 \cdot 10^{+05}$	1
439	scale_pS6_CelllineACHN_DM	-4	+4.7881	+6	1	$+6.14 \cdot 10^{+04}$	1
440	scale_pS6_CelllineADRr	-4	+2.5410	+6	1	$+3.48 \cdot 10^{+02}$	1
441	scale_pS6_CelllineADRr_B	-4	+4.7575	+6	1	$+5.72 \cdot 10^{+04}$	1
442	scale_pS6_CelllineADRr_B2	-4	+0.2313	+6	1	$+1.70 \cdot 10^{+00}$	1
443	scale_pS6_CelllineBT20	-4	+2.3802	+6	1	$+2.40 \cdot 10^{+02}$	1
444	scale_pS6_CelllineBxPc3	-4	+1.6127	+6	1	$+4.10 \cdot 10^{+01}$	1
445	scale_pS6_CelllineH322M	-4	+1.4234	+6	1	$+2.65 \cdot 10^{+01}$	1
446	scale_pS6_CelllineIGROV1	-4	+2.3676	+6	1	$+2.33 \cdot 10^{+02}$	1
447	scale_tEGFR_CelllineA431	-4	+2.0626	+6	1	$+1.16 \cdot 10^{+02}$	1
448	scale_tEGFR_CelllineACHN_197	-4	-1.7849	+6	1	$+1.64 \cdot 10^{-02}$	1
449	scale_tEGFR_CelllineACHN_200	-4	-1.4308	+6	1	$+3.71 \cdot 10^{-02}$	1

	name	θ_{min}	$\hat{\theta}$	θ_{max}	log	non-log $\hat{\theta}$	fitted
450	scale_tEGFR_CelllineACHN_218	-4	+5.3045	+6	1	$+2.02 \cdot 10^{+05}$	1
451	scale_tEGFR_CelllineACHN_DM	-4	+3.3842	+6	1	$+2.42 \cdot 10^{+03}$	1
452	scale_tEGFR_CelllineADRr	-4	-3.0840	+6	1	$+8.24 \cdot 10^{-04}$	1
453	scale_tEGFR_CelllineADRr_B	-4	+5.3932	+6	1	$+2.47 \cdot 10^{+05}$	1
454	scale_tEGFR_CelllineADRr_B2	-4	+4.3099	+6	1	$+2.04 \cdot 10^{+04}$	1
455	scale_tEGFR_CelllineBT20	-4	+0.9140	+6	1	$+8.20 \cdot 10^{+00}$	1
456	scale_tEGFR_CelllineBxPc3	-4	-0.0644	+6	1	$+8.62 \cdot 10^{-01}$	1
457	scale_tEGFR_CelllineH322M	-4	-0.1018	+6	1	$+7.91 \cdot 10^{-01}$	1
458	scale_tEGFR_CelllineIGROV1	-4	+1.4103	+6	1	$+2.57 \cdot 10^{+01}$	1
459	scale_tErbB2_CelllineA431	-4	+1.2239	+6	1	$+1.67 \cdot 10^{+01}$	1
460	scale_tErbB2_CelllineACHN_197	-4	+5.2307	+6	1	$+1.70 \cdot 10^{+05}$	1
461	scale_tErbB2_CelllineACHN_200	-4	+3.6580	+6	1	$+4.55 \cdot 10^{+03}$	1
462	scale_tErbB2_CelllineACHN_218	-4	-3.5468	+6	1	$+2.84 \cdot 10^{-04}$	1
463	scale_tErbB2_CelllineACHN_DM	-4	+1.5374	+6	1	$+3.45 \cdot 10^{+01}$	1
464	scale_tErbB2_CelllineADRr	-4	+0.3005	+6	1	$+2.00 \cdot 10^{+00}$	1
465	scale_tErbB2_CelllineADRr_B	-4	+3.8882	+6	1	$+7.73 \cdot 10^{+03}$	1
466	scale_tErbB2_CelllineADRr_B2	-4	+4.5314	+6	1	$+3.40 \cdot 10^{+04}$	1
467	scale_tErbB2_CelllineBT20	-4	+0.8763	+6	1	$+7.52 \cdot 10^{+00}$	1
468	scale_tErbB2_CelllineBxPc3	-4	-4.0000	+6	1	$+1.00 \cdot 10^{-04}$	1
469	scale_tErbB2_CelllineH322M	-4	-3.9994	+6	1	$+1.00 \cdot 10^{-04}$	1
470	scale_tErbB2_CelllineIGROV1	-4	-0.0263	+6	1	$+9.41 \cdot 10^{-01}$	1
471	scale_tErbB3_CelllineA431	-4	-3.3397	+6	1	$+4.57 \cdot 10^{-04}$	1
472	scale_tErbB3_CelllineACHN_197	-4	+3.5648	+6	1	$+3.67 \cdot 10^{+03}$	1
473	scale_tErbB3_CelllineACHN_200	-4	+0.6103	+6	1	$+4.08 \cdot 10^{+00}$	1
474	scale_tErbB3_CelllineACHN_218	-4	-1.0389	+6	1	$+9.14 \cdot 10^{-02}$	1
475	scale_tErbB3_CelllineACHN_DM	-4	+1.9669	+6	1	$+9.27 \cdot 10^{+01}$	1
476	scale_tErbB3_CelllineADRr	-4	+0.0982	+6	1	$+1.25 \cdot 10^{+00}$	1
477	scale_tErbB3_CelllineADRr_B	-4	+1.7955	+6	1	$+6.25 \cdot 10^{+01}$	1
478	scale_tErbB3_CelllineADRr_B2	-4	-1.9375	+6	1	$+1.15 \cdot 10^{-02}$	1
479	scale_tErbB3_CelllineBT20	-4	-1.0029	+6	1	$+9.93 \cdot 10^{-02}$	1
480	scale_tErbB3_CelllineBxPc3	-4	-3.2413	+6	1	$+5.74 \cdot 10^{-04}$	1
481	scale_tErbB3_CelllineH322M	-4	-3.4334	+6	1	$+3.69 \cdot 10^{-04}$	1
482	scale_tErbB3_CelllineIGROV1	-4	-0.3792	+6	1	$+4.18 \cdot 10^{-01}$	1
483	scale_tIGF1R_CelllineA431	-5	+1.3537	+6	1	$+2.26 \cdot 10^{+01}$	1
484	scale_tIGF1R_CelllineACHN_197	-4	+5.5678	+6	1	$+3.70 \cdot 10^{+05}$	1
485	scale_tIGF1R_CelllineACHN_200	-4	+0.8280	+6	1	$+6.73 \cdot 10^{+00}$	1
486	scale_tIGF1R_CelllineACHN_218	-4	+3.3713	+6	1	$+2.35 \cdot 10^{+03}$	1
487	scale_tIGF1R_CelllineACHN_DM	-4	+3.3790	+6	1	$+2.39 \cdot 10^{+03}$	1
488	scale_tIGF1R_CelllineADRr	-4	+1.3073	+6	1	$+2.03 \cdot 10^{+01}$	1
489	scale_tIGF1R_CelllineADRr_B	-4	+1.2130	+6	1	$+1.63 \cdot 10^{+01}$	1
490	scale_tIGF1R_CelllineADRr_B2	-4	+0.3996	+6	1	$+2.51 \cdot 10^{+00}$	1
491	scale_tIGF1R_CelllineBT20	-5	+1.0349	+6	1	$+1.08 \cdot 10^{+01}$	1
492	scale_tIGF1R_CelllineBxPc3	-5	-4.7698	+6	1	$+1.70 \cdot 10^{-05}$	1
493	scale_tIGF1R_CelllineH322M	-5	+0.3363	+6	1	$+2.17 \cdot 10^{+00}$	1
494	scale_tIGF1R_CelllineIGROV1	-4	+1.1611	+6	1	$+1.45 \cdot 10^{+01}$	1

	name	θ_{min}	$\hat{\theta}$	θ_{max}	log	non-log $\hat{\theta}$	fitted
495	sd_FACS_EGFR	-5	-1.2000	+3	1	$+6.31 \cdot 10^{-02}$	2
496	sd_FACS_ErbB2	-5	-1.2000	+3	1	$+6.31 \cdot 10^{-02}$	2
497	sd_FACS_ErbB3	-5	-1.2000	+3	1	$+6.31 \cdot 10^{-02}$	2
498	sd_FACS_IGF1R	-5	-1.2000	+3	1	$+6.31 \cdot 10^{-02}$	2
499	sd_FACS_Met	-5	-1.2000	+3	1	$+6.31 \cdot 10^{-02}$	2
500	sd_pAKT_au_CelllineA431	-3	-1.0000	+6	1	$+1.00 \cdot 10^{-01}$	2
501	sd_pAKT_au_CelllineACHN_197	-3	-1.0000	+6	1	$+1.00 \cdot 10^{-01}$	2
502	sd_pAKT_au_CelllineACHN_200	-3	-1.0000	+6	1	$+1.00 \cdot 10^{-01}$	2
503	sd_pAKT_au_CelllineACHN_218	-3	-1.0000	+6	1	$+1.00 \cdot 10^{-01}$	2
504	sd_pAKT_au_CelllineACHN_DM	-3	-1.0000	+6	1	$+1.00 \cdot 10^{-01}$	2
505	sd_pAKT_au_CelllineADRr	-3	-1.0000	+6	1	$+1.00 \cdot 10^{-01}$	2
506	sd_pAKT_au_CelllineADRr_B	-3	-1.0000	+6	1	$+1.00 \cdot 10^{-01}$	2
507	sd_pAKT_au_CelllineADRr_B2	-3	-1.0000	+6	1	$+1.00 \cdot 10^{-01}$	2
508	sd_pAKT_au_CelllineBT20	-3	-1.0000	+6	1	$+1.00 \cdot 10^{-01}$	2
509	sd_pAKT_au_CelllineBxPc3	-3	-1.0000	+6	1	$+1.00 \cdot 10^{-01}$	2
510	sd_pAKT_au_CelllineH322M	-3	-1.0000	+6	1	$+1.00 \cdot 10^{-01}$	2
511	sd_pAKT_au_CelllineIGROV1	-3	-1.0000	+6	1	$+1.00 \cdot 10^{-01}$	2
512	sd_pEGFR_au_CelllineA431	-3	-1.0000	+6	1	$+1.00 \cdot 10^{-01}$	2
513	sd_pEGFR_au_CelllineACHN_197	-3	-1.0000	+6	1	$+1.00 \cdot 10^{-01}$	2
514	sd_pEGFR_au_CelllineACHN_200	-3	-1.0000	+6	1	$+1.00 \cdot 10^{-01}$	2
515	sd_pEGFR_au_CelllineACHN_218	-3	-1.0000	+6	1	$+1.00 \cdot 10^{-01}$	2
516	sd_pEGFR_au_CelllineACHN_DM	-3	-1.0000	+6	1	$+1.00 \cdot 10^{-01}$	2
517	sd_pEGFR_au_CelllineADRr	-3	-1.0000	+6	1	$+1.00 \cdot 10^{-01}$	2
518	sd_pEGFR_au_CelllineADRr_B	-3	-1.0000	+6	1	$+1.00 \cdot 10^{-01}$	2
519	sd_pEGFR_au_CelllineADRr_B2	-3	-1.0000	+6	1	$+1.00 \cdot 10^{-01}$	2
520	sd_pEGFR_au_CelllineBT20	-3	-1.0000	+6	1	$+1.00 \cdot 10^{-01}$	2
521	sd_pEGFR_au_CelllineBxPc3	-3	-1.0000	+6	1	$+1.00 \cdot 10^{-01}$	2
522	sd_pEGFR_au_CelllineH322M	-3	-1.0000	+6	1	$+1.00 \cdot 10^{-01}$	2
523	sd_pEGFR_au_CelllineIGROV1	-3	-1.0000	+6	1	$+1.00 \cdot 10^{-01}$	2
524	sd_pERK_au_CelllineA431	-3	-1.0000	+6	1	$+1.00 \cdot 10^{-01}$	2
525	sd_pERK_au_CelllineACHN_197	-3	-1.0000	+6	1	$+1.00 \cdot 10^{-01}$	2
526	sd_pERK_au_CelllineACHN_200	-3	-1.0000	+6	1	$+1.00 \cdot 10^{-01}$	2
527	sd_pERK_au_CelllineACHN_218	-3	-1.0000	+6	1	$+1.00 \cdot 10^{-01}$	2
528	sd_pERK_au_CelllineACHN_DM	-3	-1.0000	+6	1	$+1.00 \cdot 10^{-01}$	2
529	sd_pERK_au_CelllineADRr	-3	-1.0000	+6	1	$+1.00 \cdot 10^{-01}$	2
530	sd_pERK_au_CelllineADRr_B	-3	-1.0000	+6	1	$+1.00 \cdot 10^{-01}$	2
531	sd_pERK_au_CelllineADRr_B2	-3	-1.0000	+6	1	$+1.00 \cdot 10^{-01}$	2
532	sd_pERK_au_CelllineBT20	-3	-1.0000	+6	1	$+1.00 \cdot 10^{-01}$	2
533	sd_pERK_au_CelllineBxPc3	-3	-1.0000	+6	1	$+1.00 \cdot 10^{-01}$	2
534	sd_pERK_au_CelllineH322M	-3	-1.0000	+6	1	$+1.00 \cdot 10^{-01}$	2
535	sd_pERK_au_CelllineIGROV1	-3	-1.0000	+6	1	$+1.00 \cdot 10^{-01}$	2
536	sd_pErbB2_au_CelllineA431	-3	-1.0000	+6	1	$+1.00 \cdot 10^{-01}$	2
537	sd_pErbB2_au_CelllineACHN_197	-3	-1.0000	+6	1	$+1.00 \cdot 10^{-01}$	2
538	sd_pErbB2_au_CelllineACHN_200	-3	-1.0000	+6	1	$+1.00 \cdot 10^{-01}$	2
539	sd_pErbB2_au_CelllineACHN_218	-3	-1.0000	+6	1	$+1.00 \cdot 10^{-01}$	2

	name	θ_{min}	$\hat{\theta}$	θ_{max}	log	non-log $\hat{\theta}$	fitted
540	sd_pErbB2_au_CelllineACHN_DM	-3	-1.0000	+6	1	$+1.00 \cdot 10^{-01}$	2
541	sd_pErbB2_au_CelllineADRr	-3	-1.0000	+6	1	$+1.00 \cdot 10^{-01}$	2
542	sd_pErbB2_au_CelllineADRr_B	-3	-1.0000	+6	1	$+1.00 \cdot 10^{-01}$	2
543	sd_pErbB2_au_CelllineADRr_B2	-3	-1.0000	+6	1	$+1.00 \cdot 10^{-01}$	2
544	sd_pErbB2_au_CelllineBT20	-3	-1.0000	+6	1	$+1.00 \cdot 10^{-01}$	2
545	sd_pErbB2_au_CelllineBxPc3	-3	-1.0000	+6	1	$+1.00 \cdot 10^{-01}$	2
546	sd_pErbB2_au_CelllineH322M	-3	-1.0000	+6	1	$+1.00 \cdot 10^{-01}$	2
547	sd_pErbB2_au_CelllineIGROV1	-3	-1.0000	+6	1	$+1.00 \cdot 10^{-01}$	2
548	sd_pErbB3_au_CelllineA431	-3	-1.0000	+6	1	$+1.00 \cdot 10^{-01}$	2
549	sd_pErbB3_au_CelllineACHN_197	-3	-1.0000	+6	1	$+1.00 \cdot 10^{-01}$	2
550	sd_pErbB3_au_CelllineACHN_200	-3	-1.0000	+6	1	$+1.00 \cdot 10^{-01}$	2
551	sd_pErbB3_au_CelllineACHN_218	-3	-1.0000	+6	1	$+1.00 \cdot 10^{-01}$	2
552	sd_pErbB3_au_CelllineACHN_DM	-3	-1.0000	+6	1	$+1.00 \cdot 10^{-01}$	2
553	sd_pErbB3_au_CelllineADRr	-3	-1.0000	+6	1	$+1.00 \cdot 10^{-01}$	2
554	sd_pErbB3_au_CelllineADRr_B	-3	-1.0000	+6	1	$+1.00 \cdot 10^{-01}$	2
555	sd_pErbB3_au_CelllineADRr_B2	-3	-1.0000	+6	1	$+1.00 \cdot 10^{-01}$	2
556	sd_pErbB3_au_CelllineBT20	-3	-1.0000	+6	1	$+1.00 \cdot 10^{-01}$	2
557	sd_pErbB3_au_CelllineBxPc3	-3	-1.0000	+6	1	$+1.00 \cdot 10^{-01}$	2
558	sd_pErbB3_au_CelllineH322M	-3	-1.0000	+6	1	$+1.00 \cdot 10^{-01}$	2
559	sd_pErbB3_au_CelllineIGROV1	-3	-1.0000	+6	1	$+1.00 \cdot 10^{-01}$	2
560	sd_pIGF1R_au_CelllineA431	-3	-1.0000	+6	1	$+1.00 \cdot 10^{-01}$	2
561	sd_pIGF1R_au_CelllineACHN_197	-3	-1.0000	+6	1	$+1.00 \cdot 10^{-01}$	2
562	sd_pIGF1R_au_CelllineACHN_200	-3	-1.0000	+6	1	$+1.00 \cdot 10^{-01}$	2
563	sd_pIGF1R_au_CelllineACHN_218	-3	-1.0000	+6	1	$+1.00 \cdot 10^{-01}$	2
564	sd_pIGF1R_au_CelllineACHN_DM	-3	-1.0000	+6	1	$+1.00 \cdot 10^{-01}$	2
565	sd_pIGF1R_au_CelllineADRr	-3	-1.0000	+6	1	$+1.00 \cdot 10^{-01}$	2
566	sd_pIGF1R_au_CelllineADRr_B	-3	-1.0000	+6	1	$+1.00 \cdot 10^{-01}$	2
567	sd_pIGF1R_au_CelllineADRr_B2	-3	-1.0000	+6	1	$+1.00 \cdot 10^{-01}$	2
568	sd_pIGF1R_au_CelllineBT20	-3	-1.0000	+6	1	$+1.00 \cdot 10^{-01}$	2
569	sd_pIGF1R_au_CelllineBxPc3	-3	-1.0000	+6	1	$+1.00 \cdot 10^{-01}$	2
570	sd_pIGF1R_au_CelllineH322M	-3	-1.0000	+6	1	$+1.00 \cdot 10^{-01}$	2
571	sd_pIGF1R_au_CelllineIGROV1	-3	-1.0000	+6	1	$+1.00 \cdot 10^{-01}$	2
572	sd_pMEK_au_CelllineA431	-3	-1.0000	+6	1	$+1.00 \cdot 10^{-01}$	2
573	sd_pMEK_au_CelllineACHN_197	-3	-1.0000	+6	1	$+1.00 \cdot 10^{-01}$	2
574	sd_pMEK_au_CelllineACHN_200	-3	-1.0000	+6	1	$+1.00 \cdot 10^{-01}$	2
575	sd_pMEK_au_CelllineACHN_218	-3	-1.0000	+6	1	$+1.00 \cdot 10^{-01}$	2
576	sd_pMEK_au_CelllineACHN_DM	-3	-1.0000	+6	1	$+1.00 \cdot 10^{-01}$	2
577	sd_pMEK_au_CelllineADRr	-3	-1.0000	+6	1	$+1.00 \cdot 10^{-01}$	2
578	sd_pMEK_au_CelllineADRr_B	-3	-1.0000	+6	1	$+1.00 \cdot 10^{-01}$	2
579	sd_pMEK_au_CelllineADRr_B2	-3	-1.0000	+6	1	$+1.00 \cdot 10^{-01}$	2
580	sd_pMEK_au_CelllineBT20	-3	-1.0000	+6	1	$+1.00 \cdot 10^{-01}$	2
581	sd_pMEK_au_CelllineBxPc3	-3	-1.0000	+6	1	$+1.00 \cdot 10^{-01}$	2
582	sd_pMEK_au_CelllineH322M	-3	-1.0000	+6	1	$+1.00 \cdot 10^{-01}$	2
583	sd_pMEK_au_CelllineIGROV1	-3	-1.0000	+6	1	$+1.00 \cdot 10^{-01}$	2
584	sd_pMet_au_CelllineA431	-3	-1.0000	+6	1	$+1.00 \cdot 10^{-01}$	2

	name	θ_{min}	$\hat{\theta}$	θ_{max}	log	non-log $\hat{\theta}$	fitted
585	sd_pMet_au_CelllineACHN_197	-3	-1.0000	+6	1	$+1.00 \cdot 10^{-01}$	2
586	sd_pMet_au_CelllineACHN_200	-3	-1.0000	+6	1	$+1.00 \cdot 10^{-01}$	2
587	sd_pMet_au_CelllineACHN_218	-3	-1.0000	+6	1	$+1.00 \cdot 10^{-01}$	2
588	sd_pMet_au_CelllineACHN_DM	-3	-1.0000	+6	1	$+1.00 \cdot 10^{-01}$	2
589	sd_pMet_au_CelllineADRr	-3	-1.0000	+6	1	$+1.00 \cdot 10^{-01}$	2
590	sd_pMet_au_CelllineADRr_B	-3	-1.0000	+6	1	$+1.00 \cdot 10^{-01}$	2
591	sd_pMet_au_CelllineADRr_B2	-3	-1.0000	+6	1	$+1.00 \cdot 10^{-01}$	2
592	sd_pMet_au_CelllineBT20	-3	-1.0000	+6	1	$+1.00 \cdot 10^{-01}$	2
593	sd_pMet_au_CelllineBxPc3	-3	-1.0000	+6	1	$+1.00 \cdot 10^{-01}$	2
594	sd_pMet_au_CelllineH322M	-3	-1.0000	+6	1	$+1.00 \cdot 10^{-01}$	2
595	sd_pMet_au_CelllineIGROV1	-3	-1.0000	+6	1	$+1.00 \cdot 10^{-01}$	2
596	sd_pS6K1_au_CelllineA431	-3	-1.0000	+6	1	$+1.00 \cdot 10^{-01}$	2
597	sd_pS6K1_au_CelllineACHN_197	-3	-1.0000	+6	1	$+1.00 \cdot 10^{-01}$	2
598	sd_pS6K1_au_CelllineACHN_200	-3	-1.0000	+6	1	$+1.00 \cdot 10^{-01}$	2
599	sd_pS6K1_au_CelllineACHN_218	-3	-1.0000	+6	1	$+1.00 \cdot 10^{-01}$	2
600	sd_pS6K1_au_CelllineACHN_DM	-3	-1.0000	+6	1	$+1.00 \cdot 10^{-01}$	2
601	sd_pS6K1_au_CelllineADRr	-3	-1.0000	+6	1	$+1.00 \cdot 10^{-01}$	2
602	sd_pS6K1_au_CelllineADRr_B	-3	-1.0000	+6	1	$+1.00 \cdot 10^{-01}$	2
603	sd_pS6K1_au_CelllineADRr_B2	-3	-1.0000	+6	1	$+1.00 \cdot 10^{-01}$	2
604	sd_pS6K1_au_CelllineBT20	-3	-1.0000	+6	1	$+1.00 \cdot 10^{-01}$	2
605	sd_pS6K1_au_CelllineBxPc3	-3	-1.0000	+6	1	$+1.00 \cdot 10^{-01}$	2
606	sd_pS6K1_au_CelllineH322M	-3	-1.0000	+6	1	$+1.00 \cdot 10^{-01}$	2
607	sd_pS6K1_au_CelllineIGROV1	-3	-1.0000	+6	1	$+1.00 \cdot 10^{-01}$	2
608	sd_pS6_au_CelllineA431	-3	-1.0000	+6	1	$+1.00 \cdot 10^{-01}$	2
609	sd_pS6_au_CelllineACHN_197	-3	-1.0000	+6	1	$+1.00 \cdot 10^{-01}$	2
610	sd_pS6_au_CelllineACHN_200	-3	-1.0000	+6	1	$+1.00 \cdot 10^{-01}$	2
611	sd_pS6_au_CelllineACHN_218	-3	-1.0000	+6	1	$+1.00 \cdot 10^{-01}$	2
612	sd_pS6_au_CelllineACHN_DM	-3	-1.0000	+6	1	$+1.00 \cdot 10^{-01}$	2
613	sd_pS6_au_CelllineADRr	-3	-1.0000	+6	1	$+1.00 \cdot 10^{-01}$	2
614	sd_pS6_au_CelllineADRr_B	-3	-1.0000	+6	1	$+1.00 \cdot 10^{-01}$	2
615	sd_pS6_au_CelllineADRr_B2	-3	-1.0000	+6	1	$+1.00 \cdot 10^{-01}$	2
616	sd_pS6_au_CelllineBT20	-3	-1.0000	+6	1	$+1.00 \cdot 10^{-01}$	2
617	sd_pS6_au_CelllineBxPc3	-3	-1.0000	+6	1	$+1.00 \cdot 10^{-01}$	2
618	sd_pS6_au_CelllineH322M	-3	-1.0000	+6	1	$+1.00 \cdot 10^{-01}$	2
619	sd_pS6_au_CelllineIGROV1	-3	-1.0000	+6	1	$+1.00 \cdot 10^{-01}$	2
620	sd_tEGFR_au_CelllineA431	-3	-1.0000	+6	1	$+1.00 \cdot 10^{-01}$	2
621	sd_tEGFR_au_CelllineACHN_197	-3	-1.0000	+6	1	$+1.00 \cdot 10^{-01}$	2
622	sd_tEGFR_au_CelllineACHN_200	-3	-1.0000	+6	1	$+1.00 \cdot 10^{-01}$	2
623	sd_tEGFR_au_CelllineACHN_218	-3	-1.0000	+6	1	$+1.00 \cdot 10^{-01}$	2
624	sd_tEGFR_au_CelllineACHN_DM	-3	-1.0000	+6	1	$+1.00 \cdot 10^{-01}$	2
625	sd_tEGFR_au_CelllineADRr	-3	-1.0000	+6	1	$+1.00 \cdot 10^{-01}$	2
626	sd_tEGFR_au_CelllineADRr_B	-3	-1.0000	+6	1	$+1.00 \cdot 10^{-01}$	2
627	sd_tEGFR_au_CelllineADRr_B2	-3	-1.0000	+6	1	$+1.00 \cdot 10^{-01}$	2
628	sd_tEGFR_au_CelllineBT20	-3	-1.0000	+6	1	$+1.00 \cdot 10^{-01}$	2
629	sd_tEGFR_au_CelllineBxPc3	-3	-1.0000	+6	1	$+1.00 \cdot 10^{-01}$	2

	name	θ_{min}	$\hat{\theta}$	θ_{max}	log	non-log $\hat{\theta}$	fitted
630	sd_tEGFR_au_CelllineH322M	-3	-1.0000	+6	1	$+1.00 \cdot 10^{-01}$	2
631	sd_tEGFR_au_CelllineIGROV1	-3	-1.0000	+6	1	$+1.00 \cdot 10^{-01}$	2
632	sd_tErbB2_au_CelllineA431	-3	-1.0000	+6	1	$+1.00 \cdot 10^{-01}$	2
633	sd_tErbB2_au_CelllineACHN_197	-3	-1.0000	+6	1	$+1.00 \cdot 10^{-01}$	2
634	sd_tErbB2_au_CelllineACHN_200	-3	-1.0000	+6	1	$+1.00 \cdot 10^{-01}$	2
635	sd_tErbB2_au_CelllineACHN_218	-3	-1.0000	+6	1	$+1.00 \cdot 10^{-01}$	2
636	sd_tErbB2_au_CelllineACHN_DM	-3	-1.0000	+6	1	$+1.00 \cdot 10^{-01}$	2
637	sd_tErbB2_au_CelllineADRr	-3	-1.0000	+6	1	$+1.00 \cdot 10^{-01}$	2
638	sd_tErbB2_au_CelllineADRr_B	-3	-1.0000	+6	1	$+1.00 \cdot 10^{-01}$	2
639	sd_tErbB2_au_CelllineADRr_B2	-3	-1.0000	+6	1	$+1.00 \cdot 10^{-01}$	2
640	sd_tErbB2_au_CelllineBT20	-3	-1.0000	+6	1	$+1.00 \cdot 10^{-01}$	2
641	sd_tErbB2_au_CelllineBxPc3	-3	-1.0000	+6	1	$+1.00 \cdot 10^{-01}$	2
642	sd_tErbB2_au_CelllineH322M	-3	-1.0000	+6	1	$+1.00 \cdot 10^{-01}$	2
643	sd_tErbB2_au_CelllineIGROV1	-3	-1.0000	+6	1	$+1.00 \cdot 10^{-01}$	2
644	sd_tErbB3_au_CelllineA431	-3	-1.0000	+6	1	$+1.00 \cdot 10^{-01}$	2
645	sd_tErbB3_au_CelllineACHN_197	-3	-1.0000	+6	1	$+1.00 \cdot 10^{-01}$	2
646	sd_tErbB3_au_CelllineACHN_200	-3	-1.0000	+6	1	$+1.00 \cdot 10^{-01}$	2
647	sd_tErbB3_au_CelllineACHN_218	-3	-1.0000	+6	1	$+1.00 \cdot 10^{-01}$	2
648	sd_tErbB3_au_CelllineACHN_DM	-3	-1.0000	+6	1	$+1.00 \cdot 10^{-01}$	2
649	sd_tErbB3_au_CelllineADRr	-3	-1.0000	+6	1	$+1.00 \cdot 10^{-01}$	2
650	sd_tErbB3_au_CelllineADRr_B	-3	-1.0000	+6	1	$+1.00 \cdot 10^{-01}$	2
651	sd_tErbB3_au_CelllineADRr_B2	-3	-1.0000	+6	1	$+1.00 \cdot 10^{-01}$	2
652	sd_tErbB3_au_CelllineBT20	-3	-1.0000	+6	1	$+1.00 \cdot 10^{-01}$	2
653	sd_tErbB3_au_CelllineBxPc3	-3	-1.0000	+6	1	$+1.00 \cdot 10^{-01}$	2
654	sd_tErbB3_au_CelllineH322M	-3	-1.0000	+6	1	$+1.00 \cdot 10^{-01}$	2
655	sd_tErbB3_au_CelllineIGROV1	-3	-1.0000	+6	1	$+1.00 \cdot 10^{-01}$	2
656	sd_tIGF1R_au_CelllineA431	-3	-1.0000	+6	1	$+1.00 \cdot 10^{-01}$	2
657	sd_tIGF1R_au_CelllineACHN_197	-3	-1.0000	+6	1	$+1.00 \cdot 10^{-01}$	2
658	sd_tIGF1R_au_CelllineACHN_200	-3	-1.0000	+6	1	$+1.00 \cdot 10^{-01}$	2
659	sd_tIGF1R_au_CelllineACHN_218	-3	-1.0000	+6	1	$+1.00 \cdot 10^{-01}$	2
660	sd_tIGF1R_au_CelllineACHN_DM	-3	-1.0000	+6	1	$+1.00 \cdot 10^{-01}$	2
661	sd_tIGF1R_au_CelllineADRr	-3	-1.0000	+6	1	$+1.00 \cdot 10^{-01}$	2
662	sd_tIGF1R_au_CelllineADRr_B	-3	-1.0000	+6	1	$+1.00 \cdot 10^{-01}$	2
663	sd_tIGF1R_au_CelllineADRr_B2	-3	-1.0000	+6	1	$+1.00 \cdot 10^{-01}$	2
664	sd_tIGF1R_au_CelllineBT20	-3	-1.0000	+6	1	$+1.00 \cdot 10^{-01}$	2
665	sd_tIGF1R_au_CelllineBxPc3	-3	-1.0000	+6	1	$+1.00 \cdot 10^{-01}$	2
666	sd_tIGF1R_au_CelllineH322M	-3	-1.0000	+6	1	$+1.00 \cdot 10^{-01}$	2
667	sd_tIGF1R_au_CelllineIGROV1	-3	-1.0000	+6	1	$+1.00 \cdot 10^{-01}$	2

Suppl. Table 4: Estimated parameter values
$\hat{\theta}$ indicates the estimated value of the parameters. θ_{min} and θ_{max} indicate the upper and lower bounds for the parameters. The log-column indicates if the value of a parameter was log-transformed. If log = 1 the non-log-column indicates the non-logarithmic value of the estimate. The fitted-column indicates if the parameter value was estimated (1), was temporarily fixed (0) or if its value was fixed to a constant value (2).

1.17.1 Condition dependent parameter changes

In the following, all model parameters that were changed to simulate the experimental conditions are listed. These changes comprise cell line dependent adaptations of receptor surface levels as well as the analytically derived steady states, i.e. the solution of the ODE equation system without external stimulation. Receptor surface levels to determine the relative parameters of initial receptor concentrations are given in the table in Fig. 41.

Cell-line	EGFR	HER2	ErbB3	IGF-1R	Met
**Cell lines used for model training**					
H322M	305047	69526	17984	52974	41903
BxPc-3	491421	58492	20827	40703	86574
BT-20	1365875	86856	20909	43231	43604
A431	1446255	121004	19918	87181	24296
ADRr	252245	54253	34412	117175	41684
IGROV-1	171058	93007	12897	10825	62346
**Cell lines used for model validation**					
MDA-MB-231	381279	44926	9284	19446	49020
ACHN	656461	49346	11883	33311	107201
BT-474-M3	31423	1325386	28118	35556	19861

Suppl. Figure 41: Receptor surface levels of the various cell lines used for the mechanistic model.

Init_dose_EGF	scale_Ligand * EGF_level
Init_dose_HRG	scale_Ligand * HRG_level
Init_dose_IGF1	scale_Ligand * IGF1_level
Init_dose_HGF	scale_Ligand * HGF_level
Init_dose_BTC	scale_Ligand * BTC_level
Init_EGFR	(init_EGFR *(1+isBxPc3 * (relto_init_EGFR -1) + isBT20 * (relto_BT20_init_EGFR -1) + isA431 * (relto_A431_init_EGFR -1) + isADRr * (relto_ADRr_init_EGFR -1) + isIGROV1 * (relto_IGROV_init_EGFR -1) + isACHN * (relto_ACHN_init_EGFR -1)))
Init_ErbB2	(init_ErbB2 *(1+isBxPc3 * (relto_init_ErbB2 -1) + isBT20 * (relto_BT20_init_ErbB2 -1) + isA431 * (relto_A431_init_ErbB2 -1) + isADRr * (relto_ADRr_init_ErbB2 -1) + isIGROV1 * (relto_IGROV_init_ErbB2 -1) + isACHN * (relto_ACHN_init_ErbB2 -1)))
Init_ErbB3	(init_ErbB3 *(1+isBxPc3 * (relto_init_ErbB3 -1) + isBT20 * (relto_BT20_init_ErbB3 -1) + isA431 * (relto_A431_init_ErbB3 -1) + isADRr * (relto_ADRr_init_ErbB3 -1) + isIGROV1 * (relto_IGROV_init_ErbB3 -1) + isACHN * (relto_ACHN_init_ErbB3 -1)))
Init_Met	(init_Met *(1+isBxPc3 * (relto_init_Met -1) + isBT20 * (relto_BT20_init_Met -1) + isA431 * (relto_A431_init_Met -1) + isADRr * (relto_ADRr_init_Met -1) + isIGROV1 * (relto_IGROV_init_Met -1) + isACHN * (relto_ACHN_init_Met -1)))
Init_IGF1R	(init_IGF1R *(1+isBxPc3 * (relto_init_IGF1R -1) + isBT20 * (relto_BT20_init_IGF1R -1) + isA431 * (relto_A431_init_IGF1R -1) + isADRr * (relto_ADRr_init_IGF1R -1) + isIGROV1 * (relto_IGROV_init_IGF1R -1) + isACHN * (relto_ACHN_init_IGF1R -1)))
init_EGFRi	init_EGFR* init_RTKph* (EGFR_ErbB2_basal_act* init_ErbB2* init_RTKph^3* pEGFR_phosphatase_binding* pErbB12i_phosphatase* pErbB13i_phosphatase* pMetEGFRi_phosphatase + EGFR_ErbB2_basal_act* init_ErbB2* init_RTKph^2* pEGFR_degradation* pErbB12i_phosphatase* pErbB13i_phosphatase* pMetEGFRi_phosphatase + EGFR_ErbB2_basal_act* init_ErbB2* init_RTKph^2* pEGFR_phosphatase_binding* pErbB12i_phosphatase* pErbB13_degradation* pMetEGFRi_phosphatase + EGFR_ErbB2_basal_act* init_ErbB2* init_RTKph^2* pEGFR_phosphatase_binding* pErbB12i_phosphatase* pErbB13i_phosphatase* pMetEGFR_degradation + EGFR_ErbB2_basal_act* init_ErbB2* init_RTKph* pEGFR_degradation* pErbB12i_phosphatase* pErbB13_degradation* pMetEGFRi_phosphatase + EGFR_ErbB2_basal_act* init_ErbB2* init_RTKph* pEGFR_degradation* pErbB12i_phosphatase* pErbB13i_phosphatase* pMetEGFR_degradation + EGFR_ErbB2_basal_act* init_ErbB2* init_RTKph* pEGFR_phosphatase_binding* pErbB12i_phosphatase* pErbB13_degradation* pMetEGFR_degradation + EGFR_ErbB2_basal_act* init_ErbB2* pEGFR_degradation* pErbB12i_phosphatase* pErbB13_degradation* pMetEGFR_degradation + EGFR_ErbB3_basal_act* init_ErbB3* init_RTKph^3* pEGFR_phosphatase_binding* pErbB12i_phosphatase* pErbB13i_phosphatase* pMetEGFRi_phosphatase + EGFR_ErbB3_basal_act* init_ErbB3* init_RTKph^2* pEGFR_degradation* pErbB12i_phosphatase* pErbB13i_phosphatase* pMetEGFRi_phosphatase + EGFR_ErbB3_basal_act* init_ErbB3* init_RTKph^2* pEGFR_phosphatase_binding* pErbB12_degradation* pErbB13i_phosphatase* pMetEGFRi_phosphatase + EGFR_ErbB3_basal_act* init_ErbB3* init_RTKph^2* pEGFR_phosphatase_binding* pErbB12i_phosphatase* pErbB13i_phosphatase* pMetEGFR_degradation + EGFR_ErbB3_basal_act* init_ErbB3* init_RTKph* pEGFR_degradation* pErbB12_degradation* pErbB13i_phosphatase* pMetEGFRi_phosphatase + EGFR_ErbB3_basal_act* init_ErbB3* init_RTKph* pEGFR_degradation* pErbB12i_phosphatase* pErbB13i_phosphatase* pMetEGFR_degradation + EGFR_ErbB3_basal_act* init_ErbB3* init_RTKph* pEGFR_phosphatase_binding* pErbB12_degradation* pErbB13i_phosphatase* pMetEGFR_degradation + EGFR_ErbB3_basal_act* init_ErbB3* pEGFR_degradation* pErbB12_degradation* pErbB13i_phosphatase* pMetEGFR_degradation + 2* EGFR_basal_activation* init_EGFR* init_RTKph^3* pEGFR_phosphatase_binding* pErbB12i_phosphatase* pErbB13i_phosphatase* pMetEGFRi_phosphatase + 2* EGFR_basal_activation* init_EGFR* init_RTKph^2* pEGFR_phosphatase_binding* pErbB12_degradation* pErbB13i_phosphatase* pMetEGFRi_phosphatase + 2* EGFR_basal_activation* init_EGFR* init_RTKph^2* pEGFR_phosphatase_binding* pErbB12i_phosphatase* pErbB13_degradation* pMetEGFRi_phosphatase + 2* EGFR_basal_activation* init_EGFR* init_RTKph^2* pEGFR_phosphatase_binding* pErbB12i_phosphatase* pErbB13i_phosphatase* pMetEGFR_degradation + 2* EGFR_basal_activation* init_EGFR* init_RTKph* pEGFR_phosphatase_binding* pErbB12_degradation* pErbB13_degradation*

	pMetEGFRi_phosphatase + 2* EGFR_basal_activation* init_EGFR* init_RTKph* pEGFR_phosphatase_binding* pErbB12_degradation* pErbB13i_phosphatase* pMetEGFR_degradation + 2* EGFR_basal_activation* init_EGFR* init_RTKph* pEGFR_phosphatase_binding* pErbB12i_phosphatase* pErbB13_degradation* pMetEGFR_degradation + 2* EGFR_basal_activation* init_EGFR* pEGFR_phosphatase_binding* pErbB12_degradation* pErbB13_degradation* pMetEGFR_degradation + Met_EGFR_basal_act* init_Met* init_RTKph^3* pEGFR_phosphatase_binding* pErbB12i_phosphatase* pErbB13i_phosphatase* pMetEGFRi_phosphatase + Met_EGFR_basal_act* init_Met* init_RTKph^2* pEGFR_degradation* pErbB12i_phosphatase* pErbB13i_phosphatase* pMetEGFRi_phosphatase + Met_EGFR_basal_act* init_Met* init_RTKph^2* pEGFR_phosphatase_binding* pErbB12_degradation* pErbB13i_phosphatase* pMetEGFRi_phosphatase + Met_EGFR_basal_act* init_Met* init_RTKph^2* pEGFR_phosphatase_binding* pErbB12i_phosphatase* pErbB13_degradation* pMetEGFRi_phosphatase + Met_EGFR_basal_act* init_Met* init_RTKph* pEGFR_degradation* pErbB12_degradation* pErbB13i_phosphatase* pMetEGFRi_phosphatase + Met_EGFR_basal_act* init_Met* init_RTKph* pEGFR_degradation* pErbB12i_phosphatase* pErbB13_degradation* pMetEGFRi_phosphatase + Met_EGFR_basal_act* init_Met* init_RTKph* pEGFR_phosphatase_binding* pErbB12_degradation* pErbB13_degradation* pMetEGFRi_phosphatase + Met_EGFR_basal_act* init_Met* pEGFR_degradation* pErbB12_degradation* pErbB13_degradation* pMetEGFRi_phosphatase)/(EGFR_basal_recycle* (init_RTKph^4* pEGFR_phosphatase_binding* pErbB12i_phosphatase* pErbB13i_phosphatase* pMetEGFRi_phosphatase + init_RTKph^3* pEGFR_degradation* pErbB12i_phosphatase* pErbB13i_phosphatase* pMetEGFRi_phosphatase + init_RTKph^3* pEGFR_phosphatase_binding* pErbB12_degradation* pErbB13i_phosphatase* pMetEGFRi_phosphatase + init_RTKph^3* pEGFR_phosphatase_binding* pErbB12i_phosphatase* pErbB13_degradation* pMetEGFRi_phosphatase + init_RTKph^3* pEGFR_phosphatase_binding* pErbB12i_phosphatase* pErbB13i_phosphatase* pMetEGFR_degradation + init_RTKph^2* pEGFR_degradation* pErbB12_degradation* pErbB13i_phosphatase* pMetEGFRi_phosphatase + init_RTKph^2* pEGFR_degradation* pErbB12i_phosphatase* pErbB13_degradation* pMetEGFRi_phosphatase + init_RTKph^2* pEGFR_degradation* pErbB12i_phosphatase* pErbB13i_phosphatase* pMetEGFR_degradation + init_RTKph^2* pEGFR_phosphatase_binding* pErbB12_degradation* pErbB13_degradation* pMetEGFRi_phosphatase + init_RTKph^2* pEGFR_phosphatase_binding* pErbB12_degradation* pErbB13i_phosphatase* pMetEGFR_degradation + init_RTKph^2* pEGFR_phosphatase_binding* pErbB12i_phosphatase* pErbB13_degradation* pMetEGFR_degradation + init_RTKph* pEGFR_degradation* pErbB12_degradation* pErbB13_degradation* pMetEGFRi_phosphatase + init_RTKph* pEGFR_degradation* pErbB12_degradation* pErbB13i_phosphatase* pMetEGFR_degradation + init_RTKph* pEGFR_degradation* pErbB12i_phosphatase* pErbB13_degradation* pMetEGFR_degradation + init_RTKph* pEGFR_phosphatase_binding* pErbB12_degradation* pErbB13_degradation* pMetEGFR_degradation + pEGFR_degradation* pErbB12_degradation* pErbB13_degradation* pMetEGFR_degradation))
init_Meti	init_Met* init_RTKph* (Met_EGFR_basal_act* init_EGFR* init_RTKph^2* pMetEGFRi_phosphatase* pMetErbB3i_phosphatase* pMeti_phosphatase + Met_EGFR_basal_act* init_EGFR* init_RTKph* pMetEGFRi_phosphatase* pMetErbB3_degradation* pMeti_phosphatase + Met_EGFR_basal_act* init_EGFR* init_RTKph* pMetEGFRi_phosphatase* pMetErbB3i_phosphatase* pMet_degradation + Met_EGFR_basal_act* init_EGFR* pMetEGFRi_phosphatase* pMetErbB3_degradation* pMet_degradation + Met_ErbB3_basal_act* init_ErbB3* init_RTKph^2* pMetEGFRi_phosphatase* pMetErbB3i_phosphatase* pMeti_phosphatase + Met_ErbB3_basal_act* init_ErbB3* init_RTKph* pMetEGFR_degradation* pMetErbB3i_phosphatase* pMeti_phosphatase + Met_ErbB3_basal_act* init_ErbB3* init_RTKph* pMetEGFRi_phosphatase* pMetErbB3i_phosphatase* pMet_degradation + Met_ErbB3_basal_act* init_ErbB3* pMetEGFR_degradation* pMetErbB3i_phosphatase* pMet_degradation + 2* Met_basal_act* init_Met* init_RTKph^2* pMetEGFRi_phosphatase* pMetErbB3i_phosphatase* pMeti_phosphatase + 2* Met_basal_act* init_Met* init_RTKph* pMetEGFR_degradation* pMetErbB3i_phosphatase* pMeti_phosphatase + 2* Met_basal_act* init_Met* init_RTKph* pMetEGFRi_phosphatase* pMetErbB3_degradation* pMeti_phosphatase + 2* Met_basal_act* init_Met* pMetEGFR_degradation* pMetErbB3_degradation* pMeti_phosphatase)/(Met_recycle* (init_RTKph^3* pMetEGFRi_phosphatase* pMetErbB3i_phosphatase* pMeti_phosphatase + init_RTKph^2* pMetEGFR_degradation*

	pMetErbB3i_phosphatase* pMeti_phosphatase + init_RTKph^2* pMetEGFRi_phosphatase* pMetErbB3_degradation* pMeti_phosphatase + init_RTKph^2* pMetEGFRi_phosphatase* pMetErbB3i_phosphatase* pMet_degradation + init_RTKph* pMetEGFR_degradation* pMetErbB3_degradation* pMeti_phosphatase + init_RTKph* pMetEGFR_degradation* pMetErbB3i_phosphatase* pMet_degradation + init_RTKph* pMetEGFRi_phosphatase* pMetErbB3_degradation* pMet_degradation + pMetEGFR_degradation* pMetErbB3_degradation* pMet_degradation))
t_pMetEGFRih	Met_EGFR_basal_act* init_EGFR* init_Met* init_RTKph* pMetEGFRi_phosphatase /(pMetEGFRi_dephosph* (init_RTKph* pMetEGFRi_phosphatase + pMetEGFR_degradation))
init_pMetEGFRi	Met_EGFR_basal_act* init_EGFR* init_Met /(init_RTKph* pMetEGFRi_phosphatase + pMetEGFR_degradation)
Init_pMET_EGFR	Met_EGFR_basal_act* init_EGFR* init_Met/pMetEGFR_internalize
init_IGF1Ri	2* IGF1R_basal_activation* init_IGF1R^2* init_RTKph* pIGF1Ri_phosphatase/ (IGF1R_basal_recycle* (init_RTKph* pIGF1Ri_phosphatase + pIGF1R_degradation))
Init_ErbB2i	init_ErbB2* init_RTKph* (EGFR_ErbB2_basal_act* init_EGFR* init_RTKph^2* pErbB12i_phosphatase * pErbB2i_phosphatase* pErbB32i_phosphatase + EGFR_ErbB2_basal_act* init_EGFR* init_RTKph* pErbB12i_phosphatase* pErbB2_degradation* pErbB32i_phosphatase + EGFR_ErbB2_basal_act* init_EGFR* init_RTKph* pErbB12i_phosphatase* pErbB2i_phosphatase* pErbB32_degradation + EGFR_ErbB2_basal_act* init_EGFR* pErbB12i_phosphatase* pErbB2_degradation* pErbB32_degradation + 2* ErbB2_dimerize* init_ErbB2* init_RTKph^2* pErbB12i_phosphatase* pErbB2i_phosphatase* pErbB32i_phosphatase + 2* ErbB2_dimerize* init_ErbB2* init_RTKph* pErbB12_degradation* pErbB2i_phosphatase* pErbB32i_phosphatase + 2* ErbB2_dimerize* init_ErbB2* init_RTKph* pErbB12i_phosphatase* pErbB2i_phosphatase* pErbB32_degradation + 2* ErbB2_dimerize* init_ErbB2* pErbB12_degradation* pErbB2i_phosphatase* pErbB32_degradation + ErbB3_ErbB2_basal_act* init_ErbB3* init_RTKph^2* pErbB12i_phosphatase* pErbB2i_phosphatase* pErbB32i_phosphatase + ErbB3_ErbB2_basal_act* init_ErbB3* init_RTKph* pErbB12_degradation* pErbB2i_phosphatase* pErbB32i_phosphatase + ErbB3_ErbB2_basal_act* init_ErbB3* init_RTKph* pErbB12i_phosphatase* pErbB2_degradation* pErbB32i_phosphatase + ErbB3_ErbB2_basal_act* init_ErbB3* pErbB12_degradation* pErbB2_degradation* pErbB32i_phosphatase) /(ErbB2_recycle* (init_RTKph^3* pErbB12i_phosphatase * pErbB2i_phosphatase* pErbB32i_phosphatase + init_RTKph^2* pErbB12_degradation* pErbB2i_phosphatase * pErbB32i_phosphatase + init_RTKph^2* pErbB12i_phosphatase* pErbB2_degradation* pErbB32i_phosphatase + init_RTKph^2* pErbB12i_phosphatase* pErbB2i_phosphatase* pErbB32_degradation + init_RTKph* pErbB12_degradation* pErbB2_degradation* pErbB32i_phosphatase + init_RTKph* pErbB12_degradation* pErbB2i_phosphatase* pErbB32_degradation + init_RTKph* pErbB12i_phosphatase* pErbB2_degradation* pErbB32_degradation + pErbB12_degradation* pErbB2_degradation* pErbB32_degradation))
Init_ErbB2i_ph	ErbB2_dimerize* init_ErbB2^2* init_RTKph* pErbB2i_phosphatase/ (pErbB2i_dephosph* (init_RTKph* pErbB2i_phosphatase + pErbB2_degradation))
Init_pErbB32i_ph	ErbB3_ErbB2_basal_act* init_ErbB2* init_ErbB3* init_RTKph* pErbB32i_phosphatase/ (pErbB32i_dephosph* (init_RTKph* pErbB32i_phosphatase + pErbB32_degradation))
Init_pErbB2i	ErbB2_dimerize* init_ErbB2^2/(init_RTKph* pErbB2i_phosphatase + pErbB2_degradation)
Init_pErbB2	ErbB2_dimerize* init_ErbB2^2/pErbB2_internalize
Init_pMeti_ph	Met_basal_act* init_Met^2* init_RTKph* pMeti_phosphatase/(pMeti_dephosph* (init_RTKph* pMeti_phosphatase + pMet_degradation))
Init_ErbB3d	ErbB3_basal_activation* init_ErbB3^2/ErbB3_deactivation
Init_pEGFRi_ph	EGFR_basal_activation* init_EGFR^2* init_RTKph* pEGFR_phosphatase_binding/ (pEGFRi_dephosph* (init_RTKph* pEGFR_phosphatase_binding + pEGFR_degradation))
Init_pErbB32i	ErbB3_ErbB2_basal_act* init_ErbB2* init_ErbB3/ (init_RTKph* pErbB32i_phosphatase + pErbB32_degradation)
Init_pErbB32	ErbB3_ErbB2_basal_act* init_ErbB2* init_ErbB3/pErbB32_internalize
Init_pErbB12i_ph	EGFR_ErbB2_basal_act* init_EGFR* init_ErbB2* init_RTKph* pErbB12i_phosphatase/ (pErbB12i_dephosph* (init_RTKph* pErbB12i_phosphatase + pErbB12_degradation))
Init_pIGF1Ri_ph	IGF1R_basal_activation* init_IGF1R^2* init_RTKph* pIGF1Ri_phosphatase/ (pIGF1Ri_dephosph* (init_RTKph* pIGF1Ri_phosphatase + pIGF1R_degradation))
Init_pIGF1Ri	IGF1R_basal_activation* init_IGF1R^2/(init_RTKph* pIGF1Ri_phosphatase + pIGF1R_degradation)

Init_pErbB12i	EGFR_ErbB2_basal_act* init_EGFR* init_ErbB2/ (init_RTKph* pErbB12i_phosphatase + pErbB12_degradation)
Init_pIGF1Rd	IGF1R_basal_activation* init_IGF1R^2/pIGF1R_internalize
Init_pErbB12	EGFR_ErbB2_basal_act* init_EGFR* init_ErbB2/pErbB12_internalize
Init_pMeti	Met_basal_act* init_Met^2/(init_RTKph* pMeti_phosphatase + pMet_degradation)
Init_pErbB13i_ph	EGFR_ErbB3_basal_act* init_EGFR* init_ErbB3* init_RTKph* pErbB13i_phosphatase/ (pErbB13i_dephosph* (init_RTKph* pErbB13i_phosphatase + pErbB13_degradation))"
Init_pErbB13i	EGFR_ErbB3_basal_act* init_EGFR* init_ErbB3/ (init_RTKph* pErbB13i_phosphatase + pErbB13_degradation)
Init_pErbB13	EGFR_ErbB3_basal_act* init_EGFR* init_ErbB3/pErbB13_internalize
Init_pMetErbB3i_ph	Met_ErbB3_basal_act* init_ErbB3* init_Met* init_RTKph* pMetErbB3i_phosphatase/ (pMetErbB3i_dephosph* (init_RTKph* pMetErbB3i_phosphatase + pMetErbB3_degradation))
Init_pMetErbB3i	Met_ErbB3_basal_act* init_ErbB3* init_Met/ (init_RTKph* pMetErbB3i_phosphatase + pMetErbB3_degradation)
Init_pMetErbB3	Met_ErbB3_basal_act* init_ErbB3* init_Met/pMetErbB3_internalize
Init_pEGFRi	EGFR_basal_activation* init_EGFR^2/ (init_RTKph* pEGFR_phosphatase_binding + pEGFR_degradation)
Init_pEGFRd	EGFR_basal_activation* init_EGFR^2/pEGFR_internalize
Init_pMetd	Met_basal_act* init_Met^2/pMet_internalize
Met_prod	init_pMetEGFRi* pMetEGFR_degradation + init_pMetErbB3i* pMetErbB3_degradation + 2* init_pMeti* pMet_degradation
IGF1R_prod	2* init_pIGF1Ri* pIGF1R_degradation
ErbB3_basal_recycle	init_pErbB13i_ph* pErbB13i_dephosph/init_ErbB3d + init_pErbB32i_ph* pErbB32i_dephosph/init_ErbB3d + init_pMetErbB3i_ph* pMetErbB3i_dephosph/init_ErbB3d
ErbB3_prod	init_pErbB13i* pErbB13_degradation + init_pErbB32i* pErbB32_degradation + init_pMetErbB3i* pMetErbB3_degradation
ErbB2_prod	init_pErbB12i* pErbB12_degradation + 2* init_pErbB2i* pErbB2_degradation + init_pErbB32i* pErbB32_degradation
EGFR_prod	2* init_pEGFRi* pEGFR_degradation + init_pErbB12i* pErbB12_degradation + init_pErbB13i* pErbB13_degradation + init_pMetEGFRi* pMetEGFR_degradation
Init_ERK	init_pERK* pERK_dephosphorylation* pMEK_dephosphorylation* (AKT_activation_pEGFR* feedback_pAKT* init_AKT* init_pEGFRd + AKT_activation_pErbB12* feedback_pAKT* init_AKT* init_pErbB12 + AKT_activation_pErbB13* feedback_pAKT* init_AKT* init_pErbB13 + AKT_activation_pErbB32* feedback_pAKT* init_AKT* init_pErbB32 + AKT_activation_pIGF1R* AKT_internIGF1R_effect* feedback_pAKT* init_AKT* init_pIGF1Ri + AKT_activation_pIGF1R* feedback_pAKT* init_AKT* init_pIGF1Rd + AKT_activation_pMetEGFR* feedback_pAKT* init_AKT* init_pMetEGFR + AKT_activation_pMetErbB3* feedback_pAKT* init_AKT* init_pMetErbB3 + AKT_activation_pMetd* feedback_pAKT* init_AKT* init_pMetd + feedback_pERK* feedback_pERK_on_AKT* init_pERK^2* pAKT_deactivation + feedback_pERK* feedback_pS6K1* init_pERK* init_pS6K1* pAKT_deactivation + feedback_pERK* init_pERK* pAKT_deactivation + feedback_pERK_on_AKT* init_pERK* pAKT_deactivation + feedback_pS6K1* init_pS6K1* pAKT_deactivation + pAKT_deactivation)/(ERK_phosphorylation_pMEK* init_MEK* pAKT_deactivation* (MEK_internIGF1R_effect* MEK_phosphorylation_pIGF1R* feedback_pERK_on_AKT* init_pERK* init_pIGF1Ri + MEK_internIGF1R_effect* MEK_phosphorylation_pIGF1R* feedback_pS6K1* init_pIGF1Ri* init_pS6K1 + MEK_internIGF1R_effect* MEK_phosphorylation_pIGF1R* init_pIGF1Ri + MEK_phosphorylation_pEGFR* feedback_pERK_on_AKT* init_pEGFRd* init_pERK + MEK_phosphorylation_pEGFR* feedback_pS6K1* init_pEGFRd* init_pS6K1 + MEK_phosphorylation_pEGFR* init_pEGFRd + MEK_phosphorylation_pErbB12* feedback_pERK_on_AKT* init_pERK* init_pErbB12 + MEK_phosphorylation_pErbB12* feedback_pS6K1* init_pErbB12* init_pS6K1 + MEK_phosphorylation_pErbB12* init_pErbB12 + MEK_phosphorylation_pErbB13* feedback_pERK_on_AKT* init_pERK* init_pErbB13 + MEK_phosphorylation_pErbB13* feedback_pS6K1* init_pErbB13* init_pS6K1 + MEK_phosphorylation_pErbB13* init_pErbB13 + MEK_phosphorylation_pErbB32* feedback_pERK_on_AKT* init_pERK* init_pErbB32 + MEK_phosphorylation_pErbB32* feedback_pS6K1* init_pErbB32* init_pS6K1 + MEK_phosphorylation_pErbB32* init_pErbB32 + MEK_phosphorylation_pIGF1R* feedback_pERK_on_AKT* init_pERK* init_pIGF1Rd +

MEK_phosphorylation_pIGF1R* feedback_pS6K1* init_pIGF1Rd* init_pS6K1 + MEK_phosphorylation_pIGF1R* init_pIGF1Rd + MEK_phosphorylation_pMetEGFR* feedback_pERK_on_AKT* init_pERK* init_pMetEGFR + MEK_phosphorylation_pMetEGFR* feedback_pS6K1* init_pMetEGFR* init_pS6K1 + MEK_phosphorylation_pMetEGFR* init_pMetEGFR + MEK_phosphorylation_pMetErbB3* feedback_pERK_on_AKT* init_pERK* init_pMetErbB3 + MEK_phosphorylation_pMetErbB3* feedback_pS6K1* init_pMetErbB3* init_pS6K1 + MEK_phosphorylation_pMetErbB3* init_pMetErbB3 + MEK_phosphorylation_pMetd* feedback_pERK_on_AKT* init_pERK* init_pMetd + MEK_phosphorylation_pMetd* feedback_pS6K1* init_pMetd* init_pS6K1 + MEK_phosphorylation_pMetd* init_pMetd))

Init_pS6	init_S6* (S6_phosphorylation_pERK* init_pERK + S6_phosphorylation_pS6K1* init_pS6K1)/pS6_dephosphorylation
init_S6K1	init_pS6K1* pAKT_deactivation* pS6K1_dephosphorylation* (feedback_pERK_on_AKT * init_pERK + feedback_pS6K1* init_pS6K1 + 1)/(AKT_activation_pEGFR* S6K1_phosphorylation_pAKT* init_AKT* init_pEGFRd + AKT_activation_pErbB12* S6K1_phosphorylation_pAKT* init_AKT* init_pErbB12 + AKT_activation_pErbB13* S6K1_phosphorylation_pAKT* init_AKT* init_pErbB13 + AKT_activation_pErbB32* S6K1_phosphorylation_pAKT* init_AKT* init_pErbB32 + AKT_activation_pIGF1R* AKT_internIGF1R_effect* S6K1_phosphorylation_pAKT* init_AKT* init_pIGF1Ri + AKT_activation_pIGF1R* S6K1_phosphorylation_pAKT* init_AKT* init_pIGF1Rd + AKT_activation_pMetEGFR* S6K1_phosphorylation_pAKT* init_AKT* init_pMetEGFR + AKT_activation_pMetErbB3* S6K1_phosphorylation_pAKT* init_AKT* init_pMetErbB3 + AKT_activation_pMetd* S6K1_phosphorylation_pAKT* init_AKT* init_pMetd + S6K1_phosphorylation_pERK* feedback_pERK_on_AKT* init_pERK^2* pAKT_deactivation + S6K1_phosphorylation_pERK* feedback_pS6K1* init_pERK* init_pS6K1* pAKT_deactivation + S6K1_phosphorylation_pERK* init_pERK* pAKT_deactivation)
Init_pMEK	init_MEK* pAKT_deactivation* (MEK_internIGF1R_effect* MEK_phosphorylation_pIGF1R* feedback_pERK_on_AKT* init_pERK* init_pIGF1Ri + MEK_internIGF1R_effect* MEK_phosphorylation_pIGF1R* feedback_pS6K1* init_pIGF1Ri* init_pS6K1 + MEK_internIGF1R_effect* MEK_phosphorylation_pIGF1R* init_pIGF1Ri + MEK_phosphorylation_pEGFR* feedback_pERK_on_AKT* init_pEGFRd* init_pERK + MEK_phosphorylation_pEGFR* feedback_pS6K1* init_pEGFRd* init_pS6K1 + MEK_phosphorylation_pEGFR* init_pEGFRd + MEK_phosphorylation_pErbB12* feedback_pERK_on_AKT* init_pERK* init_pErbB12 + MEK_phosphorylation_pErbB12* feedback_pS6K1* init_pErbB12* init_pS6K1 + MEK_phosphorylation_pErbB12* init_pErbB12 + MEK_phosphorylation_pErbB13* feedback_pERK_on_AKT* init_pERK* init_pErbB13 + MEK_phosphorylation_pErbB13* feedback_pS6K1* init_pErbB13* init_pS6K1 + MEK_phosphorylation_pErbB13* init_pErbB13 + MEK_phosphorylation_pErbB32* feedback_pERK_on_AKT* init_pERK* init_pErbB32 + MEK_phosphorylation_pErbB32* feedback_pS6K1* init_pErbB32* init_pS6K1 + MEK_phosphorylation_pErbB32* init_pErbB32 + MEK_phosphorylation_pIGF1R* feedback_pERK_on_AKT* init_pERK* init_pIGF1Rd + MEK_phosphorylation_pIGF1R* feedback_pS6K1* init_pIGF1Rd* init_pS6K1 + MEK_phosphorylation_pIGF1R* init_pIGF1Rd + MEK_phosphorylation_pMetEGFR* feedback_pERK_on_AKT* init_pERK* init_pMetEGFR + MEK_phosphorylation_pMetEGFR* feedback_pS6K1* init_pMetEGFR* init_pS6K1 + MEK_phosphorylation_pMetEGFR* init_pMetEGFR + MEK_phosphorylation_pMetErbB3* feedback_pERK_on_AKT* init_pERK* init_pMetErbB3 + MEK_phosphorylation_pMetErbB3* feedback_pS6K1* init_pMetErbB3* init_pS6K1 + MEK_phosphorylation_pMetErbB3* init_pMetErbB3 + MEK_phosphorylation_pMetd* feedback_pERK_on_AKT* init_pERK* init_pMetd + MEK_phosphorylation_pMetd* feedback_pS6K1* init_pMetd* init_pS6K1 + MEK_phosphorylation_pMetd* init_pMetd)/(pMEK_dephosphorylation* (AKT_activation_pEGFR* feedback_pAKT* init_AKT* init_pEGFRd + AKT_activation_pErbB12* feedback_pAKT* init_AKT* init_pErbB12 + AKT_activation_pErbB13* feedback_pAKT* init_AKT* init_pErbB13 + AKT_activation_pErbB32* feedback_pAKT* init_AKT* init_pErbB32 + AKT_activation_pIGF1R* AKT_internIGF1R_effect* feedback_pAKT* init_AKT* init_pIGF1Ri + AKT_activation_pIGF1R* feedback_pAKT* init_AKT* init_pIGF1Rd + AKT_activation_pMetEGFR* feedback_pAKT* init_AKT* init_pMetEGFR + AKT_activation_pMetErbB3* feedback_pAKT* init_AKT* init_pMetErbB3 + AKT_activation_pMetd* feedback_pAKT* init_AKT* init_pMetd + feedback_pERK* feedback_pERK_on_AKT* init_pERK^2* pAKT_deactivation + feedback_pERK* feedback_pS6K1* init_pERK* init_pS6K1* pAKT_deactivation + feedback_pERK* init_pERK* pAKT_deactivation +

	feedback_pERK_on_AKT* init_pERK* pAKT_deactivation + feedback_pS6K1* init_pS6K1* pAKT_deactivation + pAKT_deactivation))
Init_pAKT	init_AKT* (AKT_activation_pEGFR* init_pEGFRd + AKT_activation_pErbB12* init_pErbB12 + AKT_activation_pErbB13* init_pErbB13 + AKT_activation_pErbB32* init_pErbB32 + AKT_activation_pIGF1R* AKT_internIGF1R_effect* init_pIGF1Ri + AKT_activation_pIGF1R* init_pIGF1Rd + AKT_activation_pMetEGFR* init_pMetEGFR + AKT_activation_pMetErbB3* init_pMetErbB3 + AKT_activation_pMetd* init_pMetd)/(pAKT_deactivation* (feedback_pERK_on_AKT* init_pERK + feedback_pS6K1* init_pS6K1 + 1))

References

[1] Niepel, M. *et al.* Profiles of basal and stimulated receptor signaling networks predict drug response in breast cancer lines. *Science signaling* **6** (2013).

[2] ATLAS Collaboration. Evidence for the Higgs-boson Yukawa coupling to tau leptons with the ATLAS detector. *Journal of High Energy Physics* **2015**, 117 (2015).

[3] Dietterich, T. G. Experimental comparison of three methods for constructing ensembles of decision trees: bagging, boosting, and randomization. *Machine Learning* **40**, 139–157 (2000).

[4] Lessmann, S., Baesens, B., Seow, H. V. & Thomas, L. C. Benchmarking state-of-the-art classification algorithms for credit scoring: An update of research. *European Journal of Operational Research* **247**, 124–136 (2015).

[5] MathWorks. Statistics and machine learning toolbox release notes. Tech. Rep., Natick, MA (2015).

[6] Schumacher, R., Mosthaf, L., Schlessinger, J., Brandenburg, D. & Ullrich, A. Insulin and insulin-like growth factor-1 binding specificity is determined by distinct regions of their cognate receptors. *Journal of Biological Chemistry* **266**, 19288–19295 (1991).

[7] Basilico, C., Arnesano, A., Galluzzo, M., Comoglio, P. M. & Michieli, P. A high affinity hepatocyte growth factor-binding site in the immunoglobulin-like region of met. *Journal of Biological Chemistry* **283**, 21267–21277 (2008).

[8] Sliwkowski, M. X. *et al.* Coexpression of erbB2 and er6B3 proteins reconstitutes a high affinity receptor for heregulin. *Journal of Biological Chemistry* **269**, 14661–14665 (1994).

[9] Macdonald-Obermann, J. L. & Pike, L. J. Different epidermal growth factor (EGF) receptor ligands show distinct kinetics and biased or partial agonism for homodimer and heterodimer formation. *Journal of Biological Chemistry* **289**, 26178–26188 (2014).

[10] Engelman, J. a. *et al.* MET amplification leads to gefitinib resistance in lung cancer by activating ERBB3 signaling. *Science (New York, N.Y.)* **316**, 1039–43 (2007). URL http://www.ncbi.nlm.nih.gov/pubmed/17463250.

[11] Oda, K., Matsuoka, Y., Funahashi, A. & Kitano, H. A comprehensive pathway map of epidermal growth factor receptor signaling. *Molecular systems biology* **1**, 2005.0010 (2005). URL http://www.pubmedcentral.nih.gov/articlerender.fcgi?artid=1681468{&}tool=pmcentrez{&}rendertype=abstract.

[12] Lai, A. Z., Abella, J. V. & Park, M. Crosstalk in Met receptor oncogenesis. *Trends in cell biology* **19**, 542–51 (2009). URL http://www.ncbi.nlm.nih.gov/pubmed/19758803.

[13] Jin, Q. & Esteva, F. J. Cross-talk between the ErbB/HER family and the type I insulin-like growth factor receptor signaling pathway in breast cancer. *Journal of mammary gland biology and neoplasia* **13**, 485–98 (2008). URL http://www.ncbi.nlm.nih.gov/pubmed/19034632.

[14] Avraham, R. & Yarden, Y. Feedback regulation of EGFR signalling: decision making by early and delayed loops. *Nature reviews. Molecular cell biology* **12**, 104–17 (2011). URL http://www.ncbi.nlm.nih.gov/pubmed/21252999.

[15] Waterman, H. & Yarden, Y. Molecular mechanisms underlying endocytosis and sorting of ErbB receptor tyrosine kinases (2001).

[16] Mendoza, M. C., Er, E. E. & Blenis, J. The Ras-ERK and PI3K-mTOR pathways: cross-talk and compensation. *Trends in biochemical sciences* **36**, 320–8 (2011). URL http://www.pubmedcentral.nih.gov/articlerender.fcgi?artid=3112285{&}tool=pmcentrez{&}rendertype=abstracthttp://dx.doi.org/10.1016/j.tibs.2011.03.006.

[17] Raue, A. *et al.* Data2Dynamics: a modeling environment tailored to parameter estimation in dynamical systems. *Bioinformatics* **31**, 3558–3560 (2015). URL http://bioinformatics.oxfordjournals.org/content/early/2015/07/03/bioinformatics.btv405.abstract.

[18] Hindmarsh, A. C. *et al.* SUNDIALS: Suite of nonlinear and differential/algebraic equation solvers. *ACM Transactions on Mathematical Software (TOMS)* **31**, 363–396 (2005).
